# Potentiating immunotherapy in “immune-cold” solid tumors through orchestrating T cell immunity via tumor-specific genetic engineering

**DOI:** 10.1016/j.xcrm.2025.102510

**Published:** 2025-12-16

**Authors:** Jiaqi He, Chunguang Zhang, Chao Liang, Wenchi Xue, Yongheng Li, Lili Dai, Chunyuan Liu, Wan-Ru Zhuang, Xianbin Ma, Ran Cheng, Yao Lei, Weidong Nie, Hai-Yan Xie

**Affiliations:** 1 State Key Laboratory of Natural and Biomimetic Drugs, School of Pharmaceutical Sciences, Chemical Biology Center, Peking University, Beijing 100191, China; 2 Peking University Ningbo Institute of Marine Medicine, Ningbo 315832, China; 3 School of Life Science, Beijing Institute of Technology, Beijing 100081, China; 4 State Key Laboratory of Crop Gene Resources and Breeding, Institute of Crop Sciences, Chinese Academy of Agricultural Sciences, Beijing 100081, China; 5 School of Medical Technology, Beijing Institute of Technology, Beijing 100081, China; 6 Key Laboratory of Carcinogenesis and Translational Research (Ministry of Education/Beijing), Department of Radiation Oncology, Peking University Cancer Hospital and Institute, Beijing 100142, China; 7 Department of Respiratory and Digestive, Qian’an Yanshan Hospital, Tangshan 064400, China; 8 Department of Otorhinolaryngology, Qian’an Yanshan Hospital, Tangshan 064400, China

**Keywords:** T cell immunotherapy, tumor-specific genetic engineering, LIGHT, anti-CD3 scFv, immune checkpoint inhibitor, chimeric antigen receptor T cell, “immune-cold” solid tumor

## Abstract

We engineer a tumor-targeted genetic plasmid vector (P_αCD3&LIGHT_) to systematically modulate T cell immunity. The tumor-specific telomerase reverse transcriptase (TERT) promoter drives simultaneous expression of tumor necrosis factor superfamily member 14 (LIGHT) and membrane-anchored anti-CD3 single-chain variable fragment (αCD3), which are important immunomodulators with closely clinical relevance. Secreted LIGHT induces high endothelial venule formation and chemokine secretion to recruit circulating lymphocytes, while remodeling extracellular matrix to facilitate immune cell penetration into tumor parenchyma. αCD3 establishes artificial immunological synapses between tumor cells and T lymphocytes. This dual mechanism synergistically establishes tertiary lymphoid structures *de novo* even within deep tumor regions, harboring stem cell-like CD8^+^ T cells and driving sustained immunity. Concurrently, αCD3-mediated T cell redirection not only amplifies TCR signaling but also reverses exhausted T cells. The orchestrated T cell immunity significantly potentiates checkpoint inhibitor and chimeric antigen receptor (CAR)-T cell therapies in “immune-cold” tumors without obvious side effects and also remarkably enhances the outcome of human CAR-T cells, demonstrating translational potential in solid tumor immunotherapy.

## Highlights

• P_αCD3&LIGHT_ specifically drives the expression of αCD3 and LIGHT in tumors
• P_αCD3&LIGHT_ synergistically enhances T cell infiltration, activation, and proliferation
• P_αCD3&LIGHT_ inhibits progression of melanoma, colon carcinoma, and breast cancer
• P_αCD3&LIGHT_ augments ICIs and CAR-T cell efficacy without obvious systemic toxicity

## Graphical abstract

**Figure:**
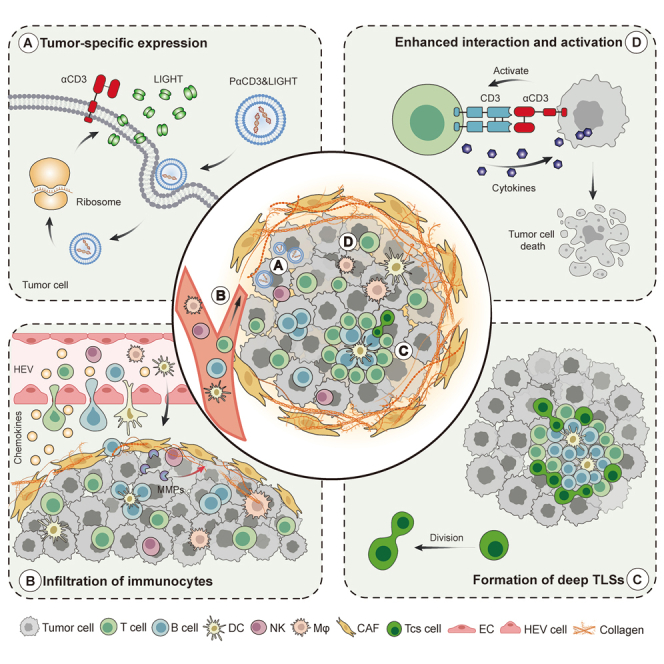

## Teaser

He et al. develop a tumor-targeted genetic plasmid vector (P_αCD3&LIGHT_) that comprehensively modulates T cell anti-tumor immunity. P_αCD3&LIGHT_ effectively suppresses the progression of multiple “immune-cold” solid tumors. Crucially, it also significantly potentiates the efficacy of ICIs and CAR-T cell therapies and reduces the systemic toxicity.

## Introduction

Current clinical immunotherapies, such as immune checkpoint blockade (ICB) and adoptive T cell therapies, aim to augment the potency of T cell immunity by activating or providing CD8-positive T lymphocytes (CD8^+^ T).^1^^,^^2^^,^^3^^,^^4^ However, the effects of CD8^+^ T in solid tumors are significantly challenged.^5^^,^^6^^,^^7^ Typically, the dense tumor stroma impedes their trafficking and infiltration; the immunosuppressive tumor microenvironment (TME) hampers their survival, proliferation, and persistence; and the intrinsic tumor cell resistance compromises their cytolytic capacity.^8^^,^^9^^,^^10^^,^^11^ Therefore, the clinical benefits of T cell-mediated immunotherapy remain limited, especially for “immune-cold” solid tumors.^8^^,^^12^^,^^13^ For instance, fewer than 10% of melanoma patients achieve long-term benefit from ICB therapy.^14^

To improve T cell trafficking toward tumors, strategies such as inhibiting the excessive growth of intratumoral blood vessels and inducing the formation of high endothelial venules (HEVs) have been established to remodel the vasculature.^15^^,^^16^^,^^17^ Establishing the concentration gradients of chemokines (such as CCL-5, CCL-19, and CXCL-9) involved in T cell recruitment can also enhance directional T cell trafficking.^18^^,^^19^ However, the dense extracellular matrix (ECM) surrounding tumors impedes the infiltration of T cells into deep tissues.^20^^,^^21^ Recent studies have revealed that stem cell-like T cells, as a subset of cells with the ability to self-renew and differentiate into effector T cells, exhibit more persistent anti-tumor activity.^22^^,^^23^ Unfortunately, maintaining their proliferation capacity remains challenging.^24^ Antigen-presenting cells (APCs) within tertiary lymphoid structures (TLSs) can initiate and sustain the proliferation of stem cell-like T cells, mediating the long-lasting T cell immune responses.^16^ However, TLS formation is hindered in the immunosuppressive TME. Tumor cells evade T cell attack primarily by downregulating major histocompatibility complex (MHC) class I expression while overexpressing immune checkpoints.^25^^,^^26^ MHC class I downregulation weakens the recognition and interaction of tumor cells with T cells.^27^^,^^28^ To address this, several bispecific T cell engagers (BiTEs) have been constructed to facilitate T cell killing of tumor cells through artificial bridges.^29^^,^^30^^,^^31^ Clinically applied immune checkpoint inhibitors (ICIs), such as anti-PD-L1 antibody and anti-PD1 antibody, prevent tumor cell immune escape by blocking immune checkpoints.^32^^,^^33^ However, these methods require T cell migration and infiltration into the tumor tissues. It is evident that any strategy addressing only one aspect cannot fundamentally improve T cell-mediated immunity. An omnipotent strategy capable of overcoming the diverse immune evasion mechanisms has yet to be established.

**Figure 1:**
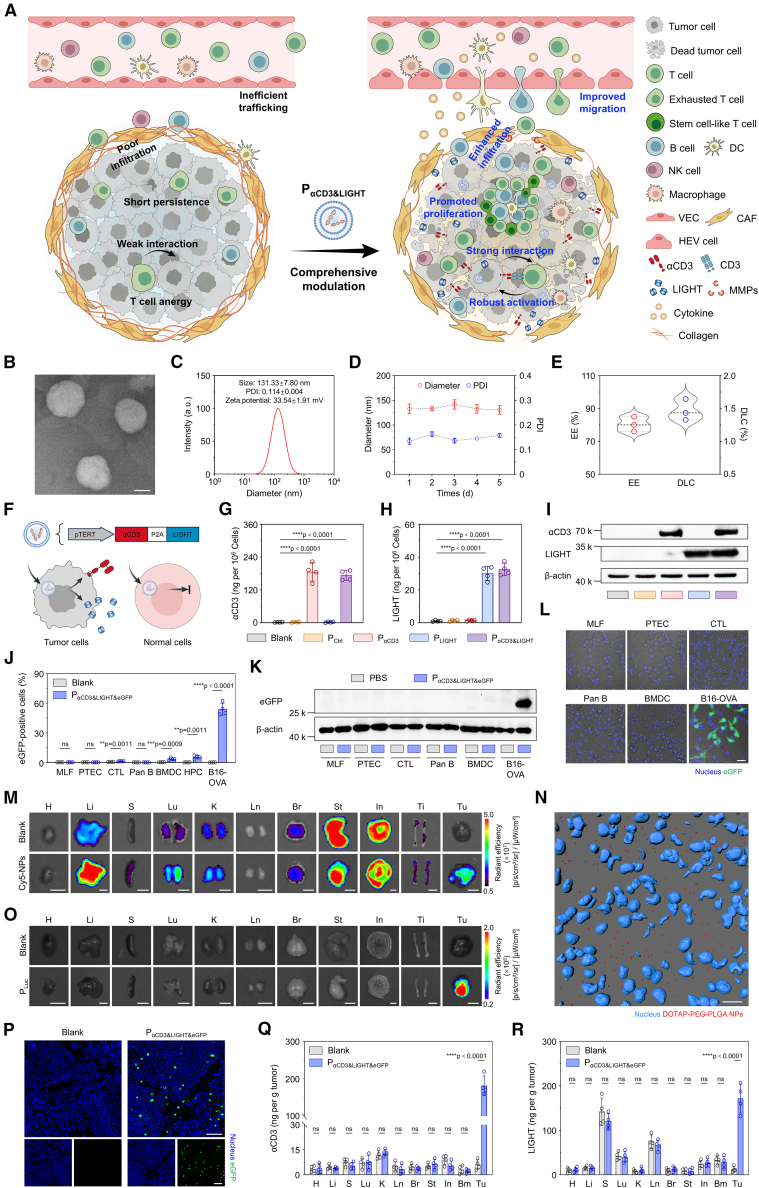
P_αCD3&LIGHT_ mediated co-expression of αCD3 and LIGHT with tumor cell specificity (A) Schematic illustration of P_αCD3&LIGHT_ comprehensively modulating T cell immunity. (B) Representative transmission electron microscopy (TEM) image of DOTAP-PEG-PLGA nanoparticles. Scale bar: 50 nm. (C) Hydrodynamic diameter, polydispersity index (PDI), and zeta potential of DOTAP-PEG-PLGA nanoparticles. *n = 3*. (D) Hydrodynamic diameter and polydispersity index (PDI) of DOTAP-PEG-PLGA nanoparticles. *n = 3*. (E) Encapsulation efficiency (EE) and drug loading capacity (DLC) of DOTAP-PEG-PLGA nanoparticles for P_αCD3&LIGHT_. *n = 3*. (F) Schematic illustration of the specific expression of αCD3 and LIGHT in tumor cells driven by P_αCD3&LIGHT_. (G–I) ELISA (G and H) and western blot analysis (I) of the expression of αCD3 and LIGHT in B16-OVA cells transfected with P_αCD3&LIGHT_ or other controls. *n = 4*. (J–L) Flow cytometry (J), western blot analysis (K), and representative CLSM images (L) of eGFP expression in lung fibroblasts (MLFs), proximal tubular epithelial cells (PTECs), cytotoxic T lymphocytes (CTLs), Pan B cells, bone marrow-derived dendritic cells (BMDCs), hematopoietic progenitor cells (HPCs), and B16-OVA tumor cells transfected with P_αCD3&LIGHT&eGFP_ or PBS. Nucleus (blue) and eGFP (green). Scale bar: 30 μm. *n = 4*. (M) IVIS spectrum images of Cy5 signals in tumors tissues (Tu), heart (H), liver (Li), spleen (S), lung (Lu), kidney (K), lymph node (Ln), brain (Br), stomach (St), intestine (In), and tibia (Ti) of melanoma-bearing mice after intravenous injection of PBS or Cy5-labeled DOTAP-PEG-PLGA nanoparticles. Scale bars: 5 mm. (N) Representative three-dimensional reconstitution images of DOTAP-PEG-PLGA nanoparticles in tumor tissues of melanoma-bearing mice after intravenous injection of Cy5-labeled DOTAP-PEG-PLGA nanoparticles. Nucleus (blue) and DOTAP-PEG-PLGA NPs (red). Scale bar: 15 μm. (O) Bioluminescence images of tumors tissues (Tu), heart (H), liver (Li), spleen (S), lung (Lu), kidney (K), lymph node (Ln), brain (Br), stomach (St), intestine (In), and tibia (Ti) of melanoma-bearing mice after intravenous injection of PBS or P_Luc_. Scale bars: 5 mm. (P) Representative immunofluorescence images of eGFP-positive cells in tumor tissues of melanoma-bearing mice after intravenous injection of P_αCD3&LIGHT&eGFP_ or PBS. Scale bars: 150 μm. (Q and R) ELISA of αCD3 (Q) and LIGHT (R) in tumors tissues (Tu), heart (H), liver (Li), spleen (S), lung (Lu), kidney (K), lymph node (Ln), brain (Br), stomach (St), intestine (In), and bone marrow (Bm) of melanoma-bearing mice after intravenous injection of P_αCD3&LIGHT&eGFP_ or PBS. *n = 4*. Data are represented as mean ± SD (error bars) from biological replicates. *p* values were determined by one-way ANOVA with Tukey’s test for (G) and (H) and unpaired two-tailed Student’s *t* test for (J), (Q), and (R). n.s., not significant; ∗∗*p* < 0.01; ∗∗∗*p* < 0.001; ∗∗∗∗*p* < 0.0001. See also Figures S1 and S2.

Herein, we propose a tumor-specific genetic engineering strategy to comprehensively elicit T cell immunity (Figure 1A). A telomerase reverse transcriptase (TERT)-dependent expression plasmid is constructed and encapsulated into (2,3-dioleoyloxy-propyl)trimethylammonium chloride (DOTAP), polyethylene glycol (PEG), and poly lactic-co-glycolic acid (PLGA) nanoparticles (DOTAP-PEG-PLGA NPs) to fabricate the tumor-specific genetic plasmid vector (P_αCD3&LIGHT_). Following intravenous (i.v.) injection, P_αCD3&LIGHT_ crosses various physiological barriers and specifically expresses in tumor cells but not in normal cells, resulting in the abundant secretion of tumor necrosis factor superfamily member 14 (LIGHT) and the decoration of anti-CD3 scFv-B7 fusion protein (αCD3) on tumor cell surface. LIGHT induces the overexpression of chemokines and vascular adhesion molecules, promoting HEV formation and enhancing T cell trafficking. Additionally, LIGHT facilitates T cell penetration into tumor parenchyma by enhancing matrix metalloproteinase (MMP)-mediated collagenolysis while inhibiting transforming growth factor β (TGF-β)-induced collagen synthesis. Accompanied by T cell trafficking and infiltration, tumor tissues are pervaded with other lymphocytes, including B cells and DC cells, fostering TLS formation, which supports the development and maintenance of stem cell-like CD8^+^ T cells and a pool of effector T cells. On the other hand, αCD3-decorated tumor cells effectively interplay with CD3^+^CD8^+^ T cells, maintaining T cell activity and reinvigorating exhausted T cells. This comprehensive improvement in T cell immunity effectively suppresses the progression of multiple “immune-cold” solid tumors in mice, including B16 melanoma, CT26 colon carcinoma, and 4T1 breast cancer, moreover, significantly promotes the efficacy of ICB and CAR-T cell therapies. Given that elevated LIGHT expression and anti-CD3 antibody administration closely correlate with improved survival in clinical, the human co-expression system (hP_αCD3&LIGHT_) was also constructed, which significantly enhances the efficacy of human CAR-T cells. These findings provide evidence for the potential clinical application of P_αCD3&LIGHT_ as a strategy to expand the immunotherapy in ”immune-cold” solid tumors.

## Results

### P_αCD3&LIGHT_ mediated co-expression of αCD3 and LIGHT with tumor specificity

Specific modification of tumor cells can enhance the targeted efficacy while minimizing potential side effects.^34^^,^^35^ Telomerase reverse transcriptase promoter (pTERT) is exceptionally activated in tumor cells, playing a crucial role in oncogenesis through the maintenance of telomere length and facilitation of cellular evasion from senescence.^36^^,^^37^ Herein, a tumor specific gene plasmid of αCD3 and LIGHT protein was constructed using pTERT (Figure S1A). To overcome the delivery barriers, plasmids were encapsulated into the NPs fabricated with cationic lipid DOTAP and PEG-PLGA. The transmission electron microscopy (TEM) imaging revealed that DOTAP-PEG-PLGA NPs exhibited spherical morphologies with well-defined boundaries (Figure 1B). Dynamic light scattering analysis of DOTAP-PEG-PLGA NPs revealed a hydrodynamic diameter (Z-average) of 131.33 ± 7.80 nm with a low polydispersity index (PDI) and a zeta potential of +33.54 ± 1.91 mV (Figure 1C). Following 5-day incubation in DMEM medium, the size and PDI remained at approximately 130 nm and <0.2, respectively, confirming the robust stability of DOTAP-PEG-PLGA NPs under physiological conditions (Figure 1D). Given that encapsulation of plasmids is a key factor for improving the transfection, the encapsulation efficiency (EE) and drug loading capacity (DLC) of DOTAP-PEG-PLGA NPs were further quantified. As shown in Figure 1E, the EE exceeded 80% and DLC reached about 1.5%, suggesting successful plasmid condensation within NPs. Meanwhile, plasmid-loaded DOTAP-PEG-PLGA NPs showed no significant hemolytic activity. (Figure S1B). These results indicated that DOTAP-PEG-PLGA NPs were suitable for delivering plasmid into tumor cells *in vitro* and *in vivo*.

Then, after transfection with P_αCD3&LIGHT_, both αCD3 and LIGHT were expressed in tumor cells (Figures 1F–1I, S1C, and S1D). The expression levels of αCD3 and LIGHT in the P_αCD3&LIGHT_ group were comparable to those in the P_αCD3_ and P_LIGHT_ groups, quantified as approximately 180 ng per 10^6^ cells for αCD3 and 30 ng per 10^6^ cells for LIGHT, respectively (Figures 1G and 1H). To evaluate tumor cell specificity, B16-ovalbumin (OVA) tumor cells and normal mouse cells, including mouse lung fibroblasts, proximal tubular epithelial cells, cytotoxic T lymphocytes (CTLs), Pan B cells, and bone marrow-derived dendritic cells (BMDCs), were transfected with eGFP gene-fused P_αCD3&LIGHT_ (P_αCD3&LIGHT&eGFP_). The results revealed that eGFP signals were observed exclusively in tumor cells but not in normal cells (Figures 1J–1L and S1E–S1J). Quantitatively, the percentage of eGFP-positive tumor cells reached 54.08% ± 6.08%, while only 3.55% ± 1.07% for BMDCs, 1.32 ± 0.24% for CTLs, and less than 0.30% for other cells (Figure 1J). Even in hematopoietic progenitors with relative proliferative potential, the eGFP-positive rate remains below 6% (Figure 1J). Given the widespread pTERT mutations in cancers,^38^ P_αCD3&LIGHT&eGFP_ was tested in other tumor cell lines, including 4T1, CT26, and MC38 cells. As expected, bright eGFP signals were observed in all these cells, highlighting the broad applicability of P_αCD3&LIGHT_ across different tumors (Figures S1K–S1M).

Subsequently, we further verified the tumor specificity of P_αCD3&LIGHT_ *in vivo*. To determine the *in vivo* biodistribution of DOTAP-PEG-PLGA NPs, melanoma-bearing mice were established and intravenously injected with Cy5-labeled DOTAP-PEG-PLGA NPs. Compared to PBS group, Cy5 fluorescence signals increased in tumors, liver, spleen, and lungs, with tumor tissues exhibiting a 10.86-fold enhancement, demonstrating efficient tumor accumulation of DOTAP-PEG-PLGA NPs (Figures 1M, S2A, S2B, and S2G). Critically, super-resolution confocal microscopy imaging revealed abundant Cy5-labeled NPs deep within the tumor tissues, demonstrating efficiently deep penetration of DOTAP-PEG-PLGA NPs into the tumor parenchyma (Figures 1N, S2C, and S2D). To determine tumor specificity of pTERT *in vivo*, a luciferase reporter plasmid (P_Luc_) was engineered through insertion of the luciferase gene at the C terminus of pTERT and then was intravenously injected into melanoma-bearing mice. Compared with the PBS group, the bioluminescence signals were clearly observed in the tumors of the P_Luc_ group, but almost no detectable in other organs (heart, liver, spleen, lung, kidney, lymph node, brain, stomach, intestine, and tibia) (Figures 1O, S2E, and S2F). Quantitatively, tumor sites exhibited 15- to 35-fold higher mean radiant efficiency than other organs, confirming that pTERT substantially counteracted off-target distribution of NPs (Figure S2H). After i.v. injection of P_αCD3&LIGHT&eGFP_, eGFP signals were exclusively detected in tumor tissues (Figure 1P), with 7.58% ± 1.12% of eGFP-positive tumor cells (Figure S2I). In contrast, almost no eGFP-positive cells were found in major organs (Figure S2J). Meanwhile, the mRNA levels of *αCD3* and *LIGHT* in the P_αCD3&LIGHT&eGFP_ group exhibited approximately 146-fold and 100-fold upregulation, respectively, relative to Blank group (Figures S2K and S2L). Consistently, the concentrations of αCD3 and LIGHT in P_αCD3&LIGHT&eGFP_-treated tumors were individually 29.50 and 13.95 times higher compared to untreated tumors (Blank group), while no significant changes were observed in normal organs (Figures 1Q and 1R). Collectively, these results demonstrated that P_αCD3&LIGHT_ could specifically and efficiently drive the expression of αCD3 and LIGHT in tumor cells, providing the foundation for the comprehensive modulation of T cell immunity.

### P_αCD3&LIGHT_ induced trafficking of immunocytes into tumors

**Figure 2:**
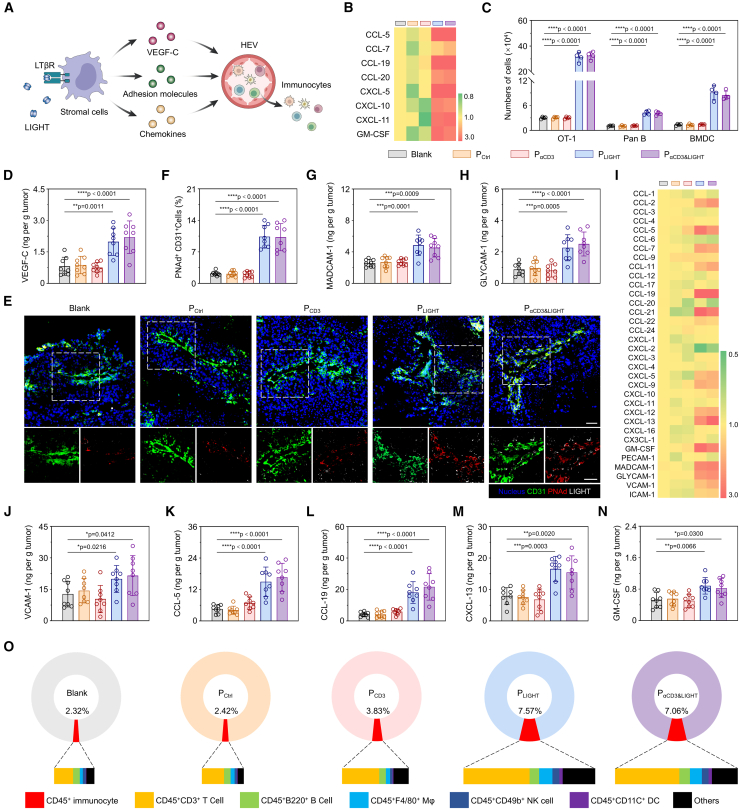
P_αCD3&LIGHT_ induced migration of immunocytes into tumor beds (A) Schematic illustration of P_αCD3&LIGHT_-induced high endothelial venule (HEV) formation and immune cell recruitment. (B) Heatmap of mRNA levels of typical chemokines secreted by cancer-associated fibroblasts (CAFs) following co-incubation with B16-OVA cells transfected with P_αCD3&LIGHT_ or other controls. (C) The numbers of migrated OT-1 cells, Pan B cells, and bone marrow-derived dendritic cells (BMDCs) after co-incubation with CAFs, C166 cells, and B16-OVA cells transfected with P_αCD3&LIGHT_ or other controls. *n =* 4. (D) ELISA of VEGF-C in tumor tissues of melanoma-bearing mice after treatment with P_αCD3&LIGHT_ or other controls. *n =* 8. (E) Representative immunofluorescence images of HEVs in tumor tissues of melanoma-bearing mice after treatment with P_αCD3&LIGHT_ or other controls. Nucleus (Blue), CD31-positive vascular endothelial cells (green), PNAd-positive HEV cells (red), and LIGHT (white). Scale bars: 50 μm. (F) Flow cytometry analysis of PNAd-positive HEV cells in CD31-positive vascular endothelial cells after treatment with P_αCD3&LIGHT_ or other controls. *n =* 8. (G and H) ELISA of MADCAM-1 (G) and GLYCAM-1 (H) in tumor tissues of melanoma-bearing mice after treatment with P_αCD3&LIGHT_ or other controls. *n =* 8. (I) Heatmap of mRNA levels of chemokines and adhesion molecules in tumor tissues of melanoma-bearing mice after treatment with P_αCD3&LIGHT_ or other controls. (J–N) ELISA of VCAM-1 (J), CCL-5 (K), CCL-19 (L), CXCL-13 (M), and GM-CSF (N) in tumor tissues of melanoma-bearing mice after treatment with P_αCD3&LIGHT_ or other controls. *n =* 8. (O) Flow cytometry analysis of various immunocytes migrating into tumor microenvironment (TME) after treatment with P_αCD3&LIGHT_ or other controls. Data are represented as mean ± SD (error bars) from biological replicates. *p* values were determined by one-way ANOVA with Tukey’s test for (C)–(H) and (J)–(N). ∗*p* < 0.05; ∗∗*p* < 0.01; ∗∗∗*p* < 0.001; ∗∗∗∗*p* < 0.0001. See also Figures S3 and S4.

5wThe directional migration of immune cells into tumors depends on chemokine gradients and normalized tumor vasculature (Figure 2A).^15^^,^^33^^,^^39^ The secreted LIGHT is able to bind lymphotoxin-β receptor on stromal cells, such as cancer-associated fibroblasts (CAFs) and vascular endothelial cells, facilitating chemokine gradient formation predominantly through activation of the non-canonical nuclear factor κB (NF-κB) signaling pathway.^40^^,^^41^ Upon co-culture with P_LIGHT_- or P_αCD3&LIGHT_-transfected tumor cells, both CAFs and vascular endothelial cells exhibited elevated level of *p100*, a key component indispensable for the non-canonical NF-κB signaling pathway (Figures S3A and S3B). As a result, the mRNA levels of typical chemokines, including *CCL-5*, *CCL-7*, *CCL-19*, *CCL-20*, *CXCL-5*, *CXCL-10*, *CXCL-11*, and *GM-CSF*, significantly upregulated compared to other groups (Figures 2B, S3C–S3J, and S3Q). Notably, *CCL-5* and *CCL-19* increased by about 8- and 13-fold, respectively (Figure 2B). Similarly, after co-incubation with P_LIGHT_- or P_αCD3&LIGHT_-transfected tumor cells, the mRNA levels of chemokines and vascular adhesion molecules in vascular endothelial cells, such as *CXCL-2*, *CXCL-8*, *CXCL-12*, *CCL-21*, *MADCAM-1*, and *VCAM-1*, rose by about 2- to 5-fold (Figures S3K–S3P and S3R). To investigate whether increased chemokine concentrations promoted immune cell migration, a transwell system was established (Figure S3S). Indeed, abundant OVA-specific CD8^+^ T cells (OT-1 cells), Pan B cells, and BMDCs migrated to the lower chamber in the P_LIGHT_ and P_αCD3&LIGHT_ groups (Figures 2C and S3T–S3Y). Only a few sporadic cells were observed in the other groups.

LIGHT can also induce the stromal cells to secrete vascular endothelial growth factor C (VEGF-C), which facilitates the development of HEVs, specialized postcapillary venules that promote lymphocyte extravasation into TME.^42^ We found that after the melanoma-bearing mice were administrated with different treatments, the VEGF-C content in tumor tissues significantly increased in the P_LIGHT_ and P_αCD3&LIGHT_ groups (Figures 2D and S4A). Consequently, HEVs, identified by peripheral node addressin staining, were prominently observed (Figures 2E and 2F). Additionally, the expression levels of MADCAM-1, GLYCAM-1, and VCAM-1 were significantly increased in HEV cells (Figures 2G, 2H, 2J, and S4B–S4D), facilitating immunocyte adhesion and rolling on HEVs.^43^^,^^44^ On the other hand, consistent with the *in vitro* results, the levels of up to 11 chemokines in tumors (Figures 2I and S4E–S4P), including *CCL-5*, *CCL-19*, *CXCL-13*, and *GM-CSF*, markedly increased in the P_LIGHT_ and P_αCD3&LIGHT_ groups (Figures 2K–2N). The comprehensive enhancement of vascular system, vascular adhesion molecules, and chemokines led to the significant accumulation of immune cells, including T cells, B cells, macrophages, natural kill cells, and dendritic cells, in tumors (Figures 2O and S4Q–S4V). Specifically, the percentage of CD45^+^CD3^+^ T cells increased from 1.09% in the Blank group to 3.70% in the P_αCD3&LIGHT_ group (Figure 2O). In summary, P_αCD3&LIGHT_ significantly promoted the directional migration of immune cells into solid tumors.

### P_αCD3&LIGHT_ facilitated infiltration of immunocytes and formation of TLSs

**Figure 3:**
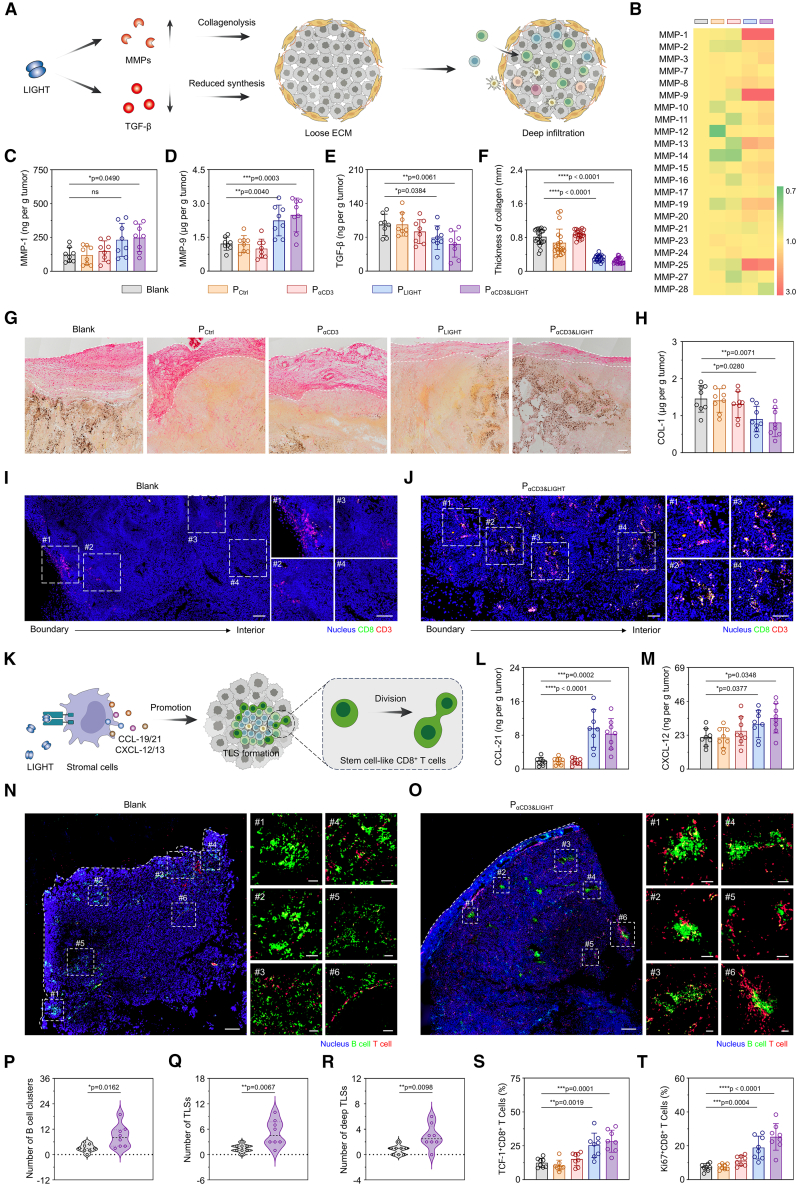
P_αCD3&LIGHT_ facilitated infiltration of immunocytes and formation of tertiary lymphoid structures (A) Schematic illustration of P_αCD3&LIGHT_-promoted immune cell penetration into tumor parenchyma. (B) Heatmap of mRNA levels of matrix metalloproteinases (MMPs) in tumor tissues of melanoma-bearing mice after treatment with P_αCD3&LIGHT_ or other controls. (C–E) ELISA of MMP-1 (C), MMP-9 (D), and TGF-β (E) in tumor tissues of melanoma-bearing mice after treatment with P_αCD3&LIGHT_ or other controls. *n =* 8. (F) Quantification of collagen thickness in tumor boundary in the P_αCD3&LIGHT_ or other control groups. *n =* 20. (G) Representative immunohistochemistry images of Picro-Sirius Red (PS-Red) staining to evaluate collagen fibers in tumor boundary in the P_αCD3&LIGHT_ or other control groups. Scale bar: 200 μm. (H) ELISA of type I collagen in tumor tissues of melanoma-bearing mice after treatment with P_αCD3&LIGHT_ or other controls. *n = 8*. (I and J) Representative immunofluorescence images of CD3^+^CD8^+^ T cells in tumor tissues of the melanoma-bearing mice after treatment with PBS (I) or P_αCD3&LIGHT_ (J). Nucleus (blue), CD8^+^ T cells (green), and CD3^+^ T cells (red). Scale bars: 200 μm. (K) Schematic illustration of TLS formation induced by LIGHT and its function in fostering stem-like T cells. (L and M) ELISA of CCL-21 (L) and CXCL-12 (M) in tumor tissues of melanoma-bearing mice after treatment with P_αCD3&LIGHT_ or other controls. *n =* 8. (N and O) Representative immunofluorescence images of TLSs in tumor tissues of the melanoma-bearing mice after treatment with P_αCD3&LIGHT_ or PBS. Nucleus (blue), B220^+^ B cells (green), and CD3^+^ T cells (red). Scale bars: 300 μm (original images) or 50 μm (enlarged images). (P–R) The numbers of B cell clusters (P), total TLSs (Q), and deep TLSs (R) in tumor tissues after treatment with P_αCD3&LIGHT_ or other controls. *n =* 8. (S and T) Flow cytometry analysis of TCF-1-positive stem cell-like CD8^+^ T cells (S) and Ki67-positive self-renewing CD8^+^ T cells (T) in the P_αCD3&LIGHT_ or other control groups. *n =* 8. Data are represented as mean ± SD (error bars) from biological replicates. *p* values were determined by one-way ANOVA with Tukey’s test for (C)–(F), (H), (L), (M), (S), and (T) and unpaired two-tailed Student’s *t* test for (P)–(R). n.s., not significant; ∗*p* < 0.05; ∗∗*p* < 0.01; ∗∗∗*p* < 0.001; ∗∗∗∗*p* < 0.0001. See also Figure S5.

Despite efficient trafficking, the tumor ECM, characterized by dense collagen and high stiffness, severely impedes deep immunocyte infiltration and thus limits their interaction with tumor cells (Figure 3A).^45^^,^^46^^,^^47^ Fortunately, LIGHT significantly promoted the secretion of various MMPs in CAFs (Figures 3B and S5A–S5E), including MMP-1, MMP-9, and MMP-25, facilitating collagenolysis and elastolysis of pre-existing collagen (Figures 3C, 3D, and S4F). Meanwhile, the content of interleukin (IL)-10 decreased by about 60% (Figure S5G), and regulatory T cells (Tregs) were depleted by approximately 70% (Figure S5H). The alleviated immunosuppressive TME led to a remarkable reduction in intratumoral TGF-β levels (Figure 3E), thereby inhibiting collagen synthesis. Thanks to the synergistic effects of synthesis inhibition and collagenolysis promotion, the dense and rigid collagen layer was effectively disrupted (Figures 3F and 3G), and small collagen fibers inside tumors were also decomposed (Figures S5J and S5K). Quantitatively, the content of type I collagen (COL-1), the most abundant collagen, decreased by approximately 45% (Figure 3H), and hydroxyproline (Hyp), a collagen-specific amino acid, reduced by about 40% (Figure S5L). This comprehensive ECM remodeling paved the way for immune cell penetration into the tumor parenchyma. As an illustration, while T cells in the Blank group were located in peritumoral tissues (Figure 3I), the P_αCD3&LIGHT_-treated tumors exhibited significant T cell infiltration into deep tumor areas (Figure 3J), even 6 mm from the tumor boundary (Figure S5I).

TLSs are organized aggregates of immune cells in and around which the anti-tumor activity of immune cells is usually maintained and enhanced (Figure 3K).^42^^,^^48^^,^^49^ The substantial accumulation of immune cells in tumors facilitated TLS formation. Moreover, the levels of chemokines closely related to TLS formation, including CCL-19, CCL-21, CXCL-12, and CXCL-13, increased in tumors after treatment with P_LIGHT_ or P_αCD3&LIGHT_ (Figures 2L, 2M, 3L, and 3M). In the Blank group, most B cells in tumors were loose and isolated, with only a few T cells sparsely surrounding them (Figures 3N and S5M). Notably, many compact and well-defined B cell clusters, known as germinal centers, were found in tumors of the P_αCD3&LIGHT_ group (Figures 3O and 3P). Moreover, these B cell clusters were surrounded by numerous T cells, indicating robust TLS formation (Figures S5M and S5N). Quantitatively, while the area of each TLS was similar (Figure S5O), the number of TLSs in the P_αCD3&LIGHT_ group was 5.13 ± 3.09 per tumor slice, compared to only 1.50 ± 0.93 in the Blank group (Figure 3Q). More importantly, deep immunocyte infiltration significantly promoted the formation of intratumoral TLS (Figure 3R), which harbored stem cell-like CD8^+^ T cells. Therefore, the ratio of stem cell-like CD8^+^ T cells increased by about 2.28-fold in the P_αCD3&LIGHT_ group compared to the Blank group (Figure 3S), and thus Ki67-positive self-renewing T cells augmented by about 3.45-fold (Figure 3T), driving long-term tumor-specific immune response by maintaining a pool of effector T cells.

### P_αCD3&LIGHT_ promoted intercellular interaction and T cell activation

**Figure 4:**
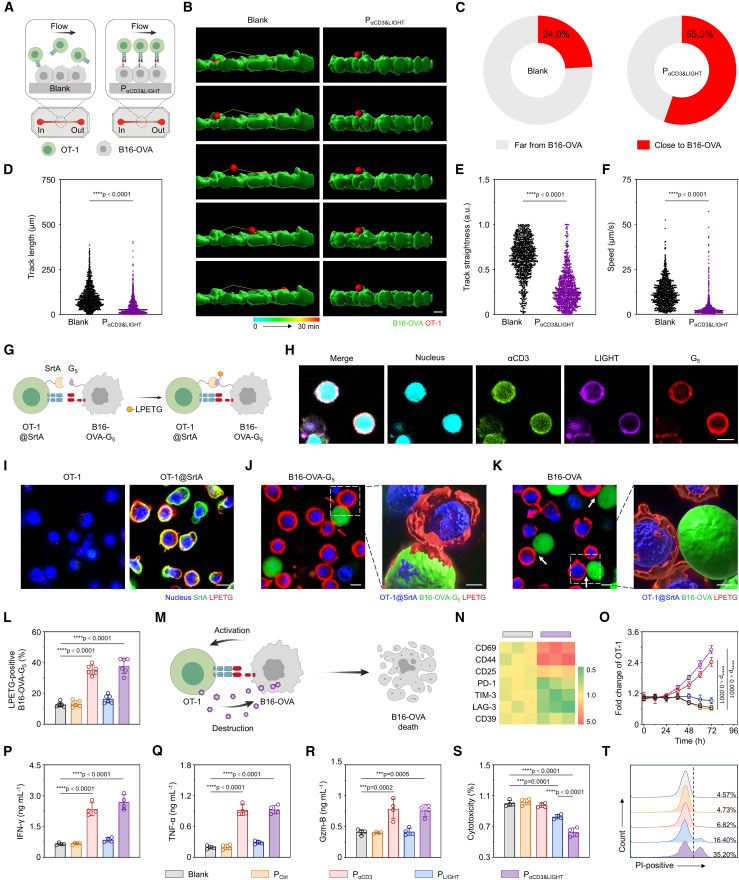
P_αCD3&LIGHT_ mediated intercellular interaction and T cell activation (A) Schematic illustration of microfluidic model for assessing the interaction between OT-1 cells and B16-OVA cells. (B) Representative three-dimensional reconstruction images and surface renderings illustrating the dynamic trajectories of OT-1 cells at various time intervals. B16-OVA cells (green) and OT-1 cells (red). Scale bar: 30 μm. (C) The percentages of OT-1 cells close to B16-OVA cells transfected with P_αCD3&LIGHT_ or PBS. *n =* 1,000. (D–F) Track length (D), track straightness (E), and speed (F) of OT-1 cells co-incubated with P_αCD3&LIGHT_-transfected B16-OVA cells or pristine B16-OVA cells. *n =* 1,000. (G) Schematic illustration of monitoring intercellular interaction via SrtA-mediated proximity labeling. (H) Representative CLMS images of G_5_ peptide on the surface of P_αCD3&LIGHT_-transfected B16-OVA cells. Nucleus (cyan), αCD3 (green), LIGHT (purple), and G_5_ peptide (red). Scale bar: 10 μm. (I) Representative CLMS images of SrtA on the surface of OT-1 cells. Nucleus (blue), SrtA (green), and LPETG peptide (red). Scale bar: 10 μm. (J and K) Representative CLMS and three-dimensional reconstitution images of the interaction between OT-1@SrtA cells and B16-OVA-G_5_ cells (J) or B16-OVA cells (K). Nucleus (blue), B16-OVA cells and B16-OVA-G_5_ cells (green), and LPETG peptide (red). The white and red arrows indicated the interface of OT-1@SrtA cells with B16-OVA cells or B16-OVA-G_5_ cells. Scale bars: 10 μm (CLMS images) or 5 μm (three-dimensional reconstitution images). (L) Flow cytometric analysis of LPETG-positive B16-OVA-G_5_ cells transfected with P_αCD3&LIGHT_ or other controls. *n =* 6. (M) Schematic illustration of T cell activation triggered by the binding of αCD3 to CD3. (N) Heatmap of mRNA levels of activation and exhaustion markers of OT-1 cells following co-incubation with B16-OVA cells transfected with P_αCD3&LIGHT_ or PBS. (O) CCK-8 assay for evaluating the number changes of OT-1 cells in the P_αCD3&LIGHT_ or other control groups. *n =* 4. (P–R) ELISA of IFN-γ (P), TNF-α (Q), and Gzm-B (R) from cell culture supernatant in the P_αCD3&LIGHT_ or other control groups. *n =* 4. (S and T) The CCK-8 assay of B16-OVA cell viability (S) and flow cytometry analysis of PI-positive apoptotic B16-OVA cells (T) in the P_αCD3&LIGHT_ or other control groups. *n =* 4. Data are represented as mean ± SD (error bars) from biological replicates. *p* values were determined by unpaired two-tailed Student’s t test for (D)–(F) and one-way ANOVA with Tukey’s test for (L) and (O)–(T). ∗∗∗*p* < 0.001; ∗∗∗∗*p* < 0.0001. See also Figure S6.

The rapid and specific recognition of tumor cells by T cells is crucial for eliciting an effective anti-tumor immune response.^50^^,^^51^ Unfortunately, most “cold” tumor cells evade T cell recognition and interaction by reducing their immunogenicity (e.g., downregulation of MHC-I).^27^^,^^52^ In view of the fact that CD3 is overexpressed on T cell surface, we hypothesized that fusing an anti-CD3 scFv to the surface of tumor cells could strengthen T cell-tumor cell interaction and overcome immune evasion. Hence, the gene of B7 transmembrane protein was fused at the 3′-terminal of anti-CD3 scFv. After transfection, the CLSM images confirmed surface expression of the anti-CD3 scFv-B7 protein (αCD3) on B16-OVA cells (Figures S6A and S6B). Quantitatively, the percentage of αCD3-positive cells reached 54.66% ± 4.33% (Figure S6C), with minimal shedding and internalization during cell proliferation (Figure S6D). To prove the potential of αCD3 in enhancing intercellular interactions, a microfluidic model was established, in which B16-OVA cells transfected with P_αCD3&LIGHT_ were immobilized on a chip, followed by the addition of OT-1 cells. After a 1-h incubation, a continuous flow was applied (Figure 4A). As could be seen, most OT-1 cells kept contact with B16-OVA cells (Figure 4B and Video S1), with 55.3% remaining in close proximity after 30 min of flow (Figure 4C). In contrast, if the B16-OVA cells were not transfected with P_αCD3&LIGHT_ (Blank group), the majority of OT-1 cells rapidly moved away (Figures 4B and 4C and Video S2). Therefore, OT-1 cells in the P_αCD3&LIGHT_ group exhibited shorter and more curved motion trajectories compared to those in the Blank group (Figures 4D and 4E). Consistently, when 1000 OT-1 cells were counted, only 38 cells exhibited a speed below 2 μm/s in the Blank group, whereas 504 cells met this criterion in the P_αCD3&LIGHT_ group (Figure 4F). Next, the intercellular interaction was visualized using a sortase A (SrtA)-mediated proximity labeling approach (Figures 4G and S6A).^53^ Briefly, B16-OVA cells were modified with five-glycine peptide (G_5_) (B16-OVA-G_5_) (Figures 4H, S6B, and S6E), achieving the decoration of up to 2 × 10^9^ G_5_ per cell (Figure S6F); simultaneously, OT-1 cells were loaded with active SrtA (OT-1@SrtA) (Figures 4I, S6G, and S6F). The substrate of SrtA, a fluorescence labeled-leu-pro-glu-thr-gly motif (LPETG), could be effectively combined to OT-1@SrtA surface and then smoothly transferred to B16-OVA-G_5_ cell surface upon the cellular interaction (Figure 4J and Video S3), but could not be transferred to pristine B16-OVA cells (Figures 4K and S6I; Video S4), as clearly shown through the three-dimensional imaging. When OT-1@SrtA cells were co-incubated with B16-OVA-G_5_ cells transfected with different plasmids, the percentage of LPETG-positive tumor cells in P_LIGHT_ group were almost unchanged but increased about 3-fold in both P_αCD3_ and P_αCD3&LIGHT_ groups relative to the Blank and P_Ctrl_ groups, again demonstrating the αCD3 promoted cellular interaction (Figure 4L).

Video S1. The most OT-1 cells (red) keep contact with B16-OVA cells (green) transfected with the PαCD3&LIGHT in microfluidic model, related to Figure 4B

Video S2. The majority of OT-1 cells (red) rapidly move away pristine B16-OVA cells (green) in microfluidic model, related to Figure 4B

Video S3. The fluorescence labeled-LPETG (Red) is smoothly transferred to the surface of B16-OVA-G5 cells (green) upon the interaction with OT-1@SrtA cells (blue), related to Figure 4J

Video S4. The fluorescence labeled-LPETG (red) is not transferred to the surface of B16-OVA cells (green) upon the interaction with OT-1@SrtA cells (blue), related to Figure 4K

As known, the binding of αCD3 to CD3 can induce T cell activation and reverse T cell exhaustion by activating the calcium-NFAT/NF-κB signaling pathways, ultimately leading to lytic granule release and consequent tumor cell destruction (Figure 4M).^54^^,^^55^^,^^56^ As expected, following a 72-h incubation with P_αCD3&LIGHT_-transfected tumor cells, the mRNA levels of *CD69*, *CD44*, and *CD25* (activation markers) were upregulated, while those of *PD-1*, *TIM-3*, *LAG-3*, and *CD39* (exhaustion markers) were downregulated, indicating effective activation and reduced exhaustion of OT-1 cells (Figure 4N). Meanwhile, flow cytometry analysis also confirmed that OT-1 cells were effectively activated (Figure S6J), leading to a significant increase in proliferation (Figure S6K), with the number increased by approximately 4 times (Figure 4O). Additionally, the levels of effector cytokines, including interferon gamma (IFN-γ), tumor necrosis factor alpha (TNF-α), and granzyme B (Gzm-B), were greatly elevated (Figures 4P–4R). Typically, TNF-α contents in the P_αCD3_ and P_αCD3&LIGHT_ groups increased approximately 4.8-fold compared to the Blank group (Figure 4Q).

The high efficiency of αCD3-promoted T cell activation, combined with LIGHT-enhanced T cell recruitment, is expected to result in robust cytotoxic effects. To verify this hypothesis, a transwell system was designed, where stromal cells and B16-OVA tumor cells transfected with different plasmids were seeded in the lower chamber, while OT-1 cells were added to the upper chamber (Figure S3S). As illustrated in Figures 4S and 4T, negligible apoptosis was observed in the P_αCD3_ group due to insufficient T cell migration. Mildly increased tumor cell death was noted in the P_LIGHT_ group, likely due to weak intercellular interaction and inefficient OT-1 activation despite enhanced recruitment. Notably, the apoptotic rate was markedly elevated in the P_αCD3&LIGHT_ group, highlighting the importance of the synergistic effect between αCD3 and LIGHT in promoting T cell-mediated immunity.

### P_αCD3&LIGHT_ mediated suppression of “immune-cold” solid tumors and enhanced therapeutic efficacy of ICIs against melanoma

**Figure 5:**
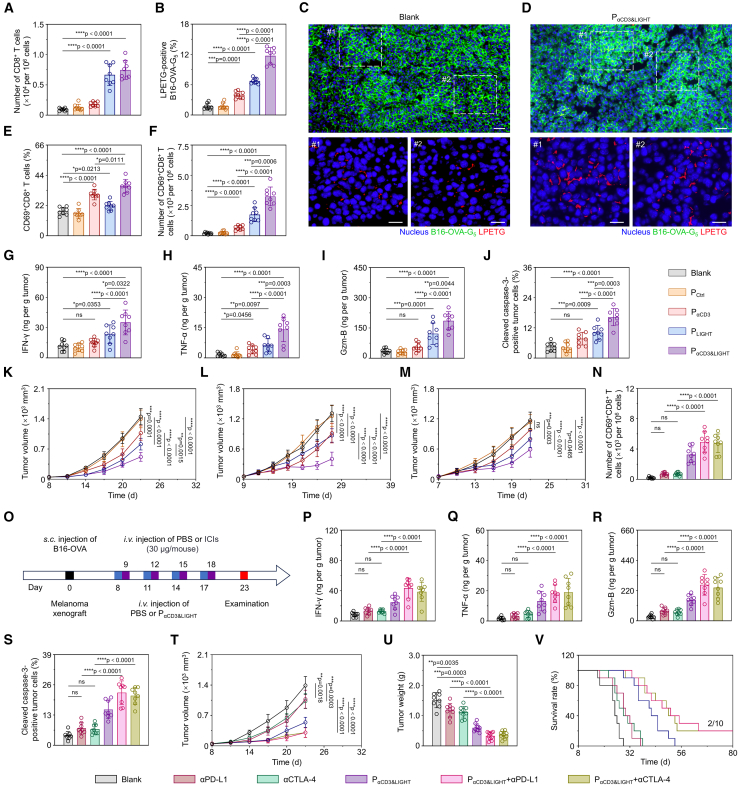
P_αCD3&LIGHT_ mediated suppression of “immune-cold” solid tumors and enhanced therapeutic efficacy of ICIs against melanoma (A and B) Flow cytometry analysis of CD8^+^ T cells (A) and LPETG-positive B16-OVA-G_5_ cells (B) in tumor tissues of melanoma-bearing mice after treatment with P_αCD3&LIGHT_ or other controls. *n =* 8*.* (C and D) Representative immunofluorescence images of LPETG-positive B16-OVA-G_5_ cells in tumor tissues of melanoma-bearing mice after treatment with P_αCD3&LIGHT_ or other controls. Nucleus (blue), B16-OVA-G_5_ cells (green), and LPETG peptide (red). Scale bars: 50 μm (original images) or 20 μm (enlarged images). (E and F) Flow cytometry analysis of the percentage (E) and count (F) of CD69^+^CD8^+^ activated T cells in tumor tissues of melanoma-bearing mice after treatment with P_αCD3&LIGHT_ or other controls. *n =* 8*.* (G–I) ELISA of IFN-γ (G), TNF-α (H), and Gzm-B (I) in tumor tissues of melanoma-bearing mice after treatment with P_αCD3&LIGHT_ or other controls. *n =* 8*.* (J) Flow cytometric analysis of cleaved caspase-3-positive apoptotic tumor cells in tumor tissues of melanoma-bearing mice after treatment with P_αCD3&LIGHT_ or other controls. *n =* 8*.* (K–M) Average tumor growth curves of the melanoma (K)-, colon carcinoma (L)-, and breast cancer (M)-bearing mice treated with P_αCD3&LIGHT_ or other controls. *n =* 8–10. (N) Flow cytometry analysis of CD69^+^CD8^+^ activated T cells in tumor tissues of melanoma-bearing mice after treatment with P_αCD3&LIGHT_ + ICIs or other controls. *n =* 8*.* (O) Therapeutic scheme of P_αCD3&LIGHT_ in combination with immune checkpoint inhibitors (ICIs). Melanoma-bearing mice were treated by four intravenous injections of P_αCD3&LIGHT_ or other controls and four intravenous injections of ICIs or PBS. (P–R) ELISA of IFN-γ (P), TNF-α (Q), and Gzm-B (R) in tumor tissues of melanoma-bearing mice after treatment with P_αCD3&LIGHT_ + ICIs or other controls. *n =* 8*.* (S) Flow cytometry analysis of cleaved caspase-3-positive apoptotic tumor cells in tumor tissues of melanoma-bearing mice after treatment with P_αCD3&LIGHT_ + ICIs or other controls. *n =* 8*.* (T–V) Average tumor growth curves (T), tumor weights (U), and survival curves (V) of melanoma-bearing mice after treatment with P_αCD3&LIGHT_ + ICIs or other controls. *n =* 8–10. Data are represented as mean ± SD (error bars) from biological replicates. *p* values were determined by one-way ANOVA with Tukey’s test for (A), (B), (E)–(N), and (P)–(U). n.s., not significant; ∗*p* < 0.05; ∗∗*p* < 0.01; ∗∗∗*p* < 0.001; ∗∗∗∗*p* < 0.0001. See also Figures S6–S10.

Inspired by the promising *in vitro* results of P_αCD3&LIGHT_, we proceeded to investigate its performance *in vivo*. Specifically, B16-OVA melanoma-bearing mice were established and subjected to treatment with P_αCD3&LIGHT_ or other controls (Figure S7A). The preliminary experiment results demonstrated that the intratumoral levels of αCD3 and LIGHT peaked at 48–72 h after administration of P_αCD3&LIGHT_ and subsequently declined gradually (Figure S7B). Thus, a repeated dosing regimen administered at 3-day intervals was implemented to sustain therapeutic protein concentrations. Moreover, anti-tumor efficacy steadily increased with escalating the dose of P_αCD3&LIGHT_ from 0 to 30 μg, plateauing at > 30 μg (Figures S7C and S7D). Consequently, 30 μg of P_αCD3&LIGHT_ was chosen for subsequent tumor inhibition studies. As expected, LIGHT significantly enhanced the migration and infiltration of CD8^+^ T cells into tumors (Figure 5A). The αCD3-enhanced cellular interaction was confirmed using the SrtA-mediated proximity labeling approach (Figures S6L and S6M). The proportion of LPETG-positive tumor cells increased from 1.69% in the Blank group to 3.99% in the P_αCD3_ group, reaching 6.75% in the P_LIGHT_ group owing to increased T cell infiltration (Figure S6N) and peaking at 11.73% in the P_αCD3&LIGHT_ group (Figures 5B–5D). Consequently, the ratio of activated CD8^+^ T cells within tumors was significantly improved in both the P_αCD3_ and P_αCD3&LIGHT_ groups (Figure 5E). Notably, LIGHT also promoted T cell activation, likely through enhanced TLS formation. The smart synergistic effect of αCD3 and LIGHT resulted in the skyrocketed number of CD69-positive CD8^+^ T in tumors of the P_αCD3&LIGHT_ group, which was 15.82-fold higher that than in untreated tumors (Blank group) (Figure 5F). The concentrations of cytotoxins in tumors, including IFN-γ, TNF-α, and Gzm-B, were markedly elevated (Figures 5G–5I). Especially, TNF-α levels increased by 8.52-fold (Figure 5H). Hence, a significant number of apoptotic tumor cells were observed in the P_αCD3&LIGHT_ group (Figure 5J). As a result, P_αCD3&LIGHT_ induced substantial tumor suppression (Figures 5K, S7E, and S7F) and significantly prolonged survival (Figure S7G). Critically, no obvious metastatic nodules were detected in common metastatic sites, including the lung, liver, and draining lymph node, (Figure S7H) and no fratricide-mediated depletion of peripheral T cells were observed (Figures S7J and S7K). Comprehensive assessment of major organ histopathology (H&E staining) (Figure S7I), serum biochemical profiling (Figures S7L–S7Q), and longitudinal monitoring of murine body temperature and weight (Figures S7R and S7S) demonstrated absence of discernible adverse pathological alterations or systemic inflammatory toxicity. Collectively, these results indicated a favorable safety profile of P_αCD3&LIGHT_. It was reasonable since pTERT selectively drove P_αCD3&LIGHT_ to express LIGHT and αCD3 in tumor cells, but not in normal tissues and normal cells, thereby significantly reducing the systemic toxicity.

To investigate the broad applicability of P_αCD3&LIGHT_, its therapeutic efficacy was further evaluated in CT26 colon carcinoma- and 4T1 breast cancer-bearing mouse models, which are also “immune-cold” solid tumors (Figures S8A and S9A). qPCR and ELISA results proved the successful expression of αCD3 and LIGHT in both CT26 (Figures S8B–S8E) and 4T1 tumors after P_αCD3&LIGHT_ treatment (Figures S9B–S9E). Accordingly, a significant upregulation of cytotoxic effector molecules (IFN-γ, TNF-α, and Gzm-B) in the P_αCD3&LIGHT_ group was observed in both CT26 (Figures S8F–S8H) and 4T1 tumors (Figures S9F–S9H). As a result, P_αCD3&LIGHT_ manifested the most powerful suppression of tumor growth, with the tumor inhibitory rate reaching 67.75% for CT26 tumors (Figures 5L, S8I, and S8J) and 50.76% for 4T1 tumors (Figures 5M, S9I, and S9J). CT26- and 4T1-bearing mice treated with P_αCD3&LIGHT_ demonstrated remarkedly prolonged median survival time (MST) (Figures S8K and S9K). Notably, no abnormalities were detected in the major organs, body weight, or body temperature, again confirming the safety profile of P_αCD3&LIGHT_ (Figures S8L–S8T and S9L–S9T). To sum up, these results indicated the efficient and universal immunomodulatory efficacy of P_αCD3&LIGHT_ in preventing the progression of “immune-cold” solid tumors.

Since melanoma is characterized as an “immune-cold” tumor with limited T cell infiltration and immunosuppressive TME,^57^ ICIs, including anti-PD-L1 antibody (αPD-L1) and anti-CTLA-4 antibody (αCTLA-4), display limited efficacy in increasing active effector T cells and inflammatory cytokines (Figures 5N–5R), resulting in only modest tumor cell death and growth inhibition at a dose of 30 μg per mouse (Figures 5S–5V). However, when ICIs were combined with P_αCD3&LIGHT_, the performance was greatly augmented. For instance, the number of activated CD8^+^ T cells increased more than 22.52- or 21.71-fold when combined with αPD-L1 or αCTLA-4 (P_αCD3&LIGHT_ + αPD-L1 or P_αCD3&LIGHT_ + αCTLA-4 group) (Figure 5N), and intertumoral cytokine levels were elevated by more than 4-fold (Figures 5P–5R), leading to a substantial increase in apoptotic tumor cells (Figure 5S). Consequently, tumor weights were reduced by over 75% (Figures 5T, 5U, and S10A), and MST was extended to 49.5 days (Figure 5V). Additionally, the combination treatments displayed a satisfactory safety profile, with no apparent organ damage and imbalances in weight and temperature (Figures S10B–S10J), providing a promising strategy for enhancing the efficacy of ICIs against “immune-cold” solid tumors.

### P_αCD3&LIGHT_ improved anti-melanoma efficacy of CAR-T cells without obvious systemic toxicity

**Figure 6:**
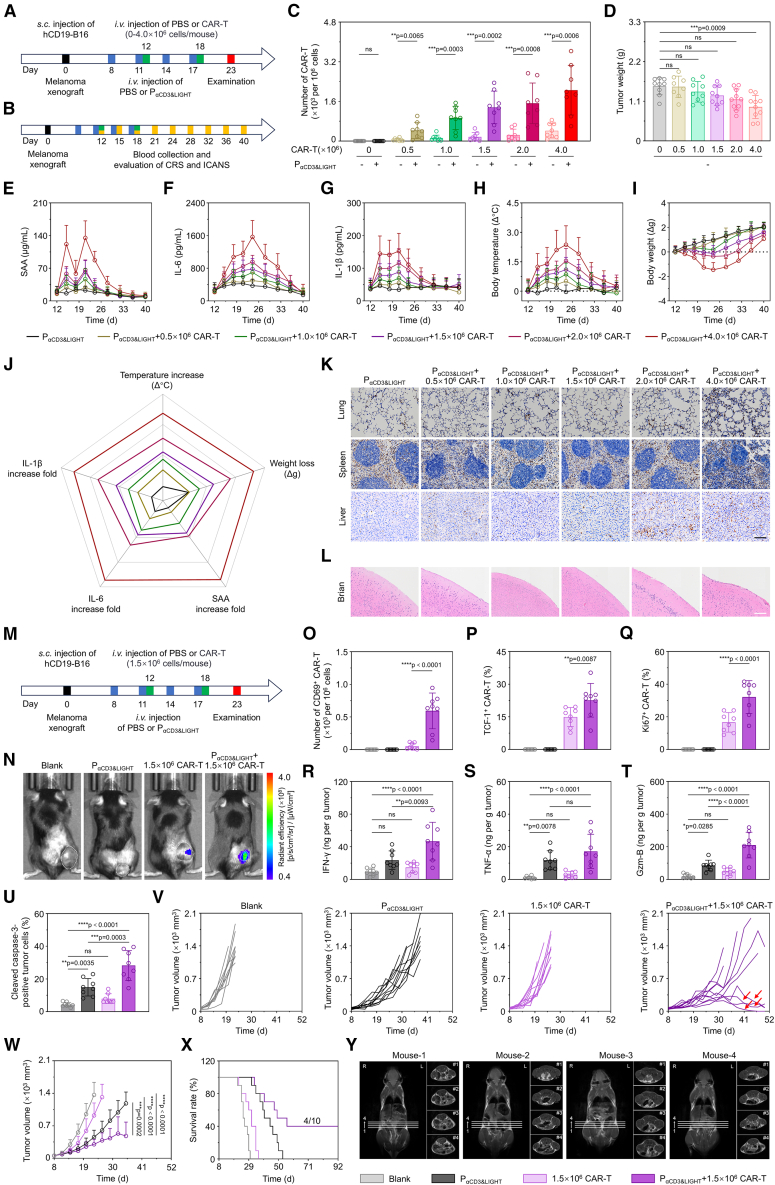
P_αCD3&LIGHT_ improved anti-melanoma efficacy of CAR-T cells without obvious systemic toxicity (A) Therapeutic scheme of P_αCD3&LIGHT_ in combination with CAR-T cells. hCD19-B16 melanoma-bearing mice were treated with four intravenous injections of P_αCD3&LIGHT_ or other controls and two intravenous injections of CAR-T cells at different doses (0–4 × 10^6^ cells per mouse). (B) Experimental timeline of blood serum collection for evaluating severe cytokine release syndrome (CRS) and immune effector cell-associated neurotoxicity syndrome (ICANS). (C) Flow cytometry analysis of CAR-T cells in tumor tissues of hCD19-B16 melanoma-bearing mice after treatment with P_αCD3&LIGHT_ + CAR-T cells or other controls. *n =* 8. (D) Tumor weights of hCD19-B16 melanoma-bearing mice treated with 0–4 × 10^6^ CAR-T cells. *n =* 9 or 10. (E–G) ELISA of SAA (E), IL-6 (F), and IL-1β (G) in blood serum of hCD19-B16 melanoma-bearing mice after treatment with P_αCD3&LIGHT_ + CAR-T cells or other controls. *n =* 8. (H and I) Body temperature (H) and body weight (I) of hCD19-B16 melanoma-bearing mice after treatment with P_αCD3&LIGHT_ + CAR-T cells or other controls. *n =* 10. (J) Radar map of fold changes of five CRS-related markers. The larger the area enclosed by the five markers, the more severe the CRS-related symptoms. (K) Representative immunohistochemistry images of CD11b-positive staining to indicate vascular leakage in lung, spleen, and liver tissues from hCD19-B16 melanoma-bearing mice after treatment with P_αCD3&LIGHT_ + CAR-T cells or other controls. Scale bar: 100 μm. (L) Representative immunohistochemistry images of H&E staining to show the thickness of meninges in brain tissues from hCD19-B16 melanoma-bearing mice after treatment with P_αCD3&LIGHT_ + CAR-T cells or other controls. Scale bar: 100 μm. (M) Therapeutic scheme of P_αCD3&LIGHT_ in combination with 1.5 × 10^6^ CAR-T cells. hCD19-B16 melanoma-bearing mice were treated with four intravenous injections of P_αCD3&LIGHT_ or other controls and two intravenous injections of PBS or total 1.5 × 10^6^ CAR-T cells. (N) Representative IVIS spectrum images of DiR-labeled CAR-T cells at tumor sites in hCD19-B16 melanoma-bearing mice after treatment with P_αCD3&LIGHT_ + 1.5 × 10^6^ CAR-T cells or other controls. (O–Q) Flow cytometry analysis of CD69-positive activated CAR-T cells (O), TCF-1-positive stem cell-like (P), and Ki67-positive self-renewing CAR-T cells (Q) in tumor tissues from hCD19-B16 melanoma-bearing mice after treatment with P_αCD3&LIGHT_ + 1.5 × 10^6^ CAR-T cells or other controls. *n =* 8. (R–T) ELISA of IFN-γ (R), TNF-α (S), and Gzm-B (T) in tumor tissues from hCD19-B16 melanoma-bearing mice after treatment with P_αCD3&LIGHT_ + 1.5 × 10^6^ CAR-T cells or other controls. *n =* 8. (U) Flow cytometry analysis of cleaved caspase-3-positive apoptotic hCD19-B16 tumor cells in tumor tissues from hCD19-B16 melanoma-bearing mice after treatment with P_αCD3&LIGHT_ + 1.5 × 10^6^ CAR-T cells or other controls. *n =* 8. (V–X) Individual (V) and average (W) tumor growth curves and survival curves (X) of hCD19-B16 melanoma-bearing mice after treatment with P_αCD3&LIGHT_ + 1.5 × 10^6^ CAR-T cells or other controls. *n =* 8–10. (Y) Magnetic resonance imaging (MRI) images of complete cured melanoma-bearing mice treated with P_αCD3&LIGHT_ + 1.5 × 10^6^ CAR-T cells. Data are represented as mean ± SD (error bars) from biological replicates. *p* values were determined by unpaired two-tailed Student’s t test for (C) and one-way ANOVA with Tukey’s test for (D), (O)–(U), and (W). n.s., not significant; ∗*p* < 0.05; ∗∗*p* < 0.01; ∗∗∗*p* < 0.001; ∗∗∗∗*p* < 0.0001. See also Figure S11.

The limited efficacy of CAR-T cells in solid tumors is primarily attributed to poor trafficking and loss of effector function, and increasing dosage is the common practice to improve treatment outcome so far.^8^^,^^58^^,^^59^^,^^60^ To closely simulate the clinical CAR-T cell therapeutic mechanisms, we engineered B16 tumor cells to express the clinically relevant human CD19 antigen (hCD19-B16), and then hCD19-B16 cells were utilized to establish solid tumor models with the ability to generate functional tumor ECM (Figure S11A). Meanwhile, we isolated anti-human CD19 scFv-expressing CAR-T cells (hαCD19-mCAR-T) from 8-week-old transgenic C57BL/6 mice, which were intravenously administered to hCD19-B16 tumor-bearing mice (Figure S11B). The hαCD19-mCAR-T cells could specifically bind hCD19-B16 cells and subsequently elicited potent cytolytic activity against these targets. It was found that the accumulation of CAR-T cells in tumors gradually increased with higher doses (Figures 6A–6C), but the infiltration rate remained about 1% (Figure S11I). Hence, tumor growth was only marginally inhibited even at dose of 4 × 10^6^ cells per mouse (Figures 6D, S11C, and S11D). However, severe cytokine release syndrome (CRS) was observed in this condition, characterized by robust inflammatory cytokine secretion and dysregulation of body weight/temperature (Figures 6E–6J and S11E–S11H). Meanwhile, administration of 4 × 10^6^ CAR-T cells induced substantial myeloid cell infiltration in the lungs, spleen, and liver compared to non-CAR-T cell-treated group, indicating serious vascular leakage and organ damage (Figure 6K). Concurrently, obvious meningeal thickening, one hallmark feature of immune effector cell-associated neurotoxicity syndrome (ICANS), was also discovered (Figure 6L). Mild hepatic injury persisted even when the CAR-T cell dose was reduced to 2.0 × 10^6^ cells per mouse (Figure 6K). Interestingly, when P_αCD3&LIGHT_ was co-administered with CAR-T cell therapy, the migration of CAR-T cells into tumors increased about 5- to 8-fold (Figures 6C and S11I). In particular, the number of CAR-T cells in tumors of P_αCD3&LIGHT_ + 1.5 × 10^6^ CAR-T cell group was more than 3.2 times that of the 4 × 10^6^ CAR-T cell group (Figure 6C), while no significant CRS was observed (Figures 6E–6J). Levels of CRS-related factors, including serum amyloid A, IL-6, and IL-1β, were remained relatively low and recovered within 6 days post-administration through self-regulation (Figures 6E–6G). Average body temperature variation was <1.5°C, and weight loss was <10% over 40 days of observation (Figures 6H and 6I). Additionally, at the reduced dose of 1.5 × 10^6^ CAR-T cells, neither myeloid cell infiltration nor meningeal thickening was observed relative to non-CAR-T cell-treated controls, further indicating avoidance of treatment-associated CRS and ICANS (Figures 6K and 6L).

Next, we comparatively evaluated the therapeutic efficacy of P_αCD3&LIGHT_ co-administrated with 1.5 × 10^6^ CAR-T cells on melanoma (Figure 6M). As expected, P_αCD3&LIGHT_ markedly enhanced intratumoral infiltration of CAR-T cells (Figures 6N, S11J, and S11K). Moreover, P_αCD3&LIGHT_ comprehensively improved the anti-tumor effects of CAR-T cells. Particularly, the number of activated CAR-T cells in tumors increased about 12-fold (Figure 6O), and the percentage of stem cell-like CAR-T cells rose by 1.52 times (Figure 6P), significantly enhancing CAR-T cell proliferation within tumors (Figure 6Q). Correspondingly, intratumoral levels of IFN-γ, TNF-α, and Gzm-B were significantly elevated (Figures 6R–6T), resulting in robust cytotoxicity against tumor cells (Figure 6U) and substantial inhibition of tumor growth (Figures 6V and 6W). Consequently, the survival rate of mice in the P_αCD3&LIGHT_ + 1.5 × 10^6^ CAR-T cell group was significantly prolonged (Figure 6X), with 4 out of 10 mice achieving complete cure and no recurrence observed over a follow-up period exceeding three months (Figure 6Y; Videos S5, S6, S7, and S8). In contrast, all mice in the 1.5 × 10^6^ CAR-T cell groups died within 36 days. To further elevate the long-term biosafety of P_αCD3&LIGHT_, we assessed the serum biochemical profiling of the 4 complete cured mice on 90-, 120-, and 150-day post-treatments with P_αCD3&LIGHT_ and 1.5 × 10^6^ CAR-T cells. As shown in Figures S11L–S11Q, the levels of alkaline phosphatase, alanine aminotransferase, aspartate transaminase, creatinine, blood urea nitrogen, and lactate dehydrogenase in mice were maintained within physiologically normal parameters, indicating a negligible treatment-related chronic toxicity. To summarize, P_αCD3&LIGHT_ significantly potentiated the performance of CAR-T cells in melanoma by orchestrating T immunity, meanwhile, reduced the systemic toxicity of CAR-T cell therapy.

Video S5. No obvious micro-tumors were observed in the first cured mouse using the MRI, related to Figure 6Y

Video S6. No obvious micro-tumors were observed in the second cured mouse using the MRI, related to Figure 6Y

Video S7. No obvious micro-tumors were observed in the third cured mouse using the MRI, related to Figure 6Y

Video S8. No obvious micro-tumors were observed in the fourth cured mouse using the MRI, related to Figure 6Y

### LIGHT and αCD3 antibody exhibited clinical therapeutic prospects, and hP_αCD3&LIGHT_ enhanced performance of human CAR-T cells

**Figure 7:**
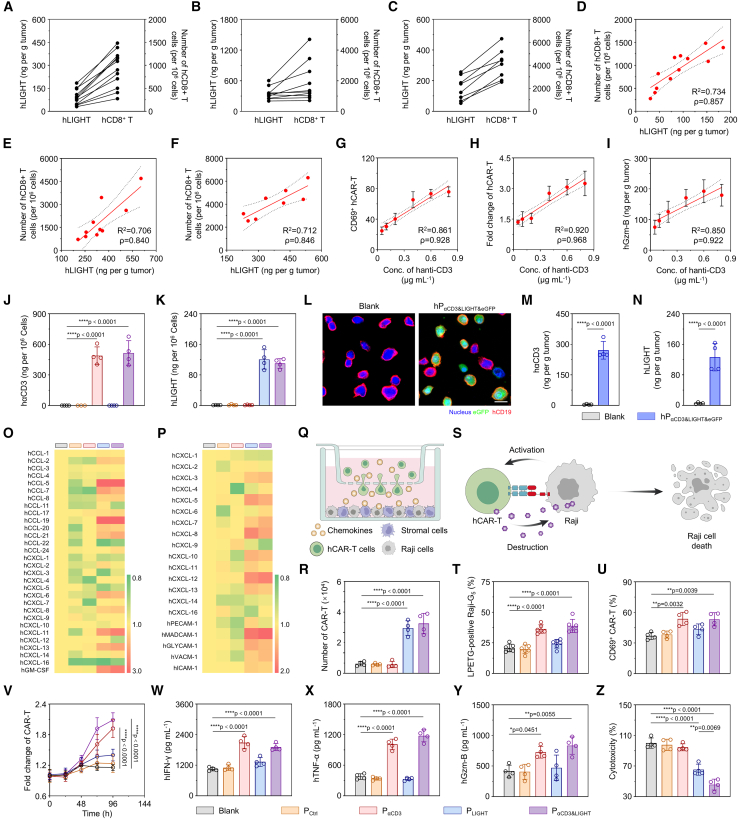
LIGHT and αCD3 antibody exhibited clinical therapeutic prospects, and hP_αCD3&LIGHT_ enhanced performance of human CAR-T cells (A–C) The levels of hLIGHT and the numbers of hCD8^+^ T cells in colorectal cancer (CRC) (A), nasopharyngeal carcinoma (NPC) (B), and cervical cancer (C). *n =* 12, 10, or 8. (D–F) Regression analysis of hLIGHT expression and the number of hCD8^+^ T cells in colorectal cancer (CRC) (D), nasopharyngeal carcinoma (NPC) (E), and cervical cancer (F). *n =* 12, 10, or 8. (G–I) Regression analysis of anti-αCD3 concentration and CD69-positive activated hCAR-T cells (G), proliferative capability (H), and hGzm-B secretion (I). *n =* 4*.* (J and K) ELISA of hαCD3 (J) and hLIGHT (K) in Raji cells transfected with hP_αCD3&LIGHT_ or other controls. *n =* 4. (L) Representative CLSM images of eGFP expression in Raji cells transfected with hP_αCD3&LIGHT&eGFP_ or PBS. Scale bar: 20 μm. (M and N) ELISA of hαCD3 (M) and hLIGHT (N) in tumor tissues of lymphoma-bearing mice after intravenous injection of hP_αCD3&LIGHT&eGFP_ or PBS. *n =* 4. (O and P) Heatmaps of the mRNA levels of chemokines and adhesion molecules secreted by hCAFs (O) and HUVECs (P) following co-incubation with Raji cells transfected with hP_αCD3&LIGHT_ or other controls. (Q) Schematic illustration of hP_αCD3&LIGHT_-mediated recruitment of hCAR-T cells. (R) The number of migrated hCAR-T cells after co-incubating hCAFs, HUVECs, and Raji cells transfected with hP_αCD3&LIGHT_ or other controls. *n =* 4. (S) Schematic illustration of hP_αCD3&LIGHT_-mediated activation of hCAR-T cells. (T) Flow cytometric analysis of LPETG-positive Raji-G_5_ cells transfected with hP_αCD3&LIGHT_ or other controls after co-incubating with hCAR-T@SrtA cells. *n =* 6. (U) Flow cytometry analysis of CD69-positive activated hCAR-T cells in the hP_αCD3&LIGHT_ or other control groups. *n =* 4. (V) CCK-8 assay for evaluating the number changes of hCAR-T cells in the hP_αCD3&LIGHT_ or other control groups. *n =* 4. (W–Y) ELISA of hIFN-γ (W), hTNF-α (X), and hGzm-B (Y) from cell culture supernatant in the hP_αCD3&LIGHT_ or other control groups. *n =* 4. (Z) The CCK-8 assay of Raji cell viability in the hP_αCD3&LIGHT_ or other control groups. *n =* 4. Data are represented as mean ± SD (error bars) from biological replicates. *p* values were determined by one-way ANOVA with Tukey’s test for (J), (K), (R), and (T)–(Z) and unpaired two-tailed Student’s *t* test for (M) and (N). ∗*p* < 0.05; ∗∗*p* < 0.01; ∗∗∗∗*p* < 0.0001. See also Figures S12 and S13.

It is reported that LIGHT level closely correlates with survival outcome in various cancers, mediated by the enhanced cytotoxic lymphocyte infiltration.^61^^,^^62^ For verification, we profiled the LIGHT expression and the CD8^+^ T cell infiltration in surgically excised specimens from colorectal cancer, nasopharyngeal carcinoma, and cervical cancer patients, followed by regression analysis to decipher their clinical and immunological interdependency. Notably, LIGHT level was significantly associated with the number of CD8^+^ T cells in all the three malignancies (R^2^ > 0.7 and ρ > 0.84) (Figures 7A–7F). The immunofluorescence analysis also revealed a significant consistency between upregulated LIGHT expression and enriched CD8^+^ T cell infiltration in tumors (Figure S13A). These results suggested that LIGHT was one of pan-cancer determinants of T cell-inflamed TMEs. To further evaluate the clinical relevance of LIGHT, its expression level and the patient survival outcome were analyzed in pan-cancer by retrieving The Cancer Genome Atlas (TCGA) datasets. As shown in Figures S12A–S12R, in 14 of 18 malignancies, elevated LIGHT expression conferred a survival advantage, with superior 3- or 5-year survival in the high-expression cohorts compared to low-expression groups, particularly in breast invasive carcinoma, rectum adenocarcinoma, liver hepatocellular carcinoma, skin cutaneous melanoma, and ovary serous cystadenocarcinoma. On the other side, the anti-CD3 antibody (anti-CD3)-targeted therapeutic pipeline has demonstrated substantial clinical advancement, with 8 approved biologics (6 monoclonal antibodies and 2 bispecific agents) achieving regulatory milestones.^63^^,^^64^ Besides, several CD3-targeted BiTE antibodies are currently undergoing clinical trials, such as blinatumomab, glofitamab, tebentafusp, talquetamab, and teclistamab, all of which could result in T cell activation (Figure S12S). Our results also confirmed that human hαCD19-hCAR-T cells (hCAR-Ts) displayed anti-CD3 dose-dependent activation (R^2^ = 0.861 and ρ = 0.928), proliferation (R^2^ = 0.920 and ρ = 0.968), and granzyme B secretion (R^2^ = 0.850 and ρ = 0.922) (Figures 7G–7I). All these demonstrated that therapeutic strategies leveraging LIGHT and anti-CD3 showed promise in clinical cancer therapy.

To explore the clinical therapeutic potential of P_αCD3&LIGHT_, we systematically investigated its effects in improving the performance of human CAR-T cells. The human co-expression system (hP_αCD3&LIGHT_) was constructed to ensure the expression of human anti-CD3 scFv-B7 fusion protein (hαCD3) and human LIGHT (hLIGHT) in human Burkitt’s lymphoma cells (Raji cells) (Figures 7J–7L and S13B–S13F). Upon i.v. injection of hP_αCD3&LIGHT_ into Raji lymphoma-bearing nude mice, the contents of hαCD3 and hLIGHT in tumor tissues were as high as 271.10 ± 43.32 ng per g tumor and 126.28 ± 35.91 ng per g tumor, respectively (Figures 7M and 7N). In view of the fact that hLIGHT is highly dependent on human stromal cells, human CAFs (hCAFs) were isolated from surgically resected tumor tissues of gastric cancer patients and co-cultured with different plasmid-transfected Raji cells. We observed significant upregulation of various chemokines, including *CCL-5*, *CCL-7*, *CCL-19*, *CXCL-6*, *CXCL-11*, *CXCL-13*, and *GM-CSF*, in hCAFs exposed to hP_LIGHT_ or hP_αCD3&LIGHT_ compared to other groups (Figures 7O and S13G–S13P). Similarly, co-incubation with hP_LIGHT_- or hP_αCD3&LIGHT_-transfected Raji cells significantly elevated chemokine levels in HUVECs (Figures 7P and S13Q–S13Y). The raised chemokines greatly promoted the migration of hCAR-Ts, as verified by a transwell assay (Figure 7Q), with an about 5-fold increase of hCAR-T cells in the lower chamber (Figure 7R). On the other hand, hαCD3 decoration significantly expanded the interaction between Raji cells and hCAR-T cells (Figures 7S and 7T), sustained hCAR-T cell activity and enhanced their proliferation (Figures 7U and 7V). These effects led to a significant increase in cytotoxic cytokine secretion (Figures 7W–7Y), particularly, hTNF-α increased about 3-fold (Figure 7X). The cooperation of hαCD3 and hLIGHT resulted in the death of most tumor cells in the lower chamber of transwell in the hP_αCD3&LIGHT_ group. In contrast, limited tumor cell destruction was observed in the hP_αCD3_ and hP_LIGHT_ groups, due to poor infiltration or ineffective activation of hCAR-T cells (Figure 7Z). Collectively, hP_αCD3&LIGHT_ drives the expression of hαCD3 and hLIGHT in humanized tumors, leading to comprehensive enhancement of T cell immunity and broadening the implications of CAR-T cell therapy against human solid tumors.

## Discussion

The immunosuppressive TME in solid tumors is a network consisting various cell types, stroma, blood vessels, secreted factors and the ECM, which seriously limits the anti-tumor efficacy of immune cells—particularly T cells.^65^^,^^66^ Consequently, numerous strategies have been developed to remodel TME, so as to enhance the infiltration and function of T cells.^65^^,^^67^ For example, physically opening the tumor barrier using photothermal agents or radiolabeled liposomes markedly increased the abundance of T cells within tumors.^68^^,^^69^ However, the limited tissue penetration depth, off-target accumulation in healthy organs, and indiscriminate killing of such strategies remain critical challenges. Anti-angiogenic agents can be used to remodel pathologically tortuous and hyperpermeable tumor vasculature, alleviating mechanical stress to potentiate T cell infiltration.^70^ Typically, Bevacizumab and Ramucirumab, as the most effective VEGF-A inhibitors, have gained Food and Drug Administration approval for treating diverse malignancies in clinic. However, these drugs usually induce significant cardiovascular toxicities, including hypertension, thromboembolic events, and cardiac dysfunction, because VEGF-A pathway is integral to vascular homeostasis under normal physiological conditions.^71^ Alternatively, antitumorigenic chemokines, the pivotal immunomodulators, are used to reprogram the immune landscape of TME,^72^ but the performance of elevating single or several types of chemokines often proves suboptimal for achieving robust T cell recruitment.^73^ On the other hand, modulating key physicochemical features of TME, such as improving hypoxia, neutralizing acidity, and eliminating reactive oxygen species (ROS), is a promising strategy to enhance the anti-tumor activity of T cells.^74^^,^^75^ Meanwhile, depleting immunosuppressive cytokines (e.g., TGF-β and IL-10) and regulatory cells (e.g., Tregs and TAMs) are fundamental to unleash functional T cell effector programs, thereby reversing T cell exhaustion and potentiating therapeutic efficacy.^76^^,^^77^ The premise of such strategies is a high-density T cell infiltrate in TME. While combinatorial regimens of T cell recruitment and activation show great potential, the antitumor efficacy may be significantly compromised owing to the failure of progress synchronization. In this study, we construct a dual-expression plasmid vector P_αCD3&LIGHT_. TERT promoter is used to control the highly tumor-specific expression of αCD3 and LIGHT, significantly reducing the systemic toxicity by minimizing off-target effects in healthy tissues. LIGHT induces stromal cells to secrete up to 11 chemokines and various adhesion molecules, collectively establishing a robust chemotactic milieu for immune cell trafficking. It also triggers HEVs formation, providing specialized conduits for lymphocyte extravasation. Importantly, LIGHT effectively disrupts the rigid collagen layer in the tumor ECM via synergism of inhibiting TGF-β-induced synthesis and promoting MMP-mediated collagenolysis, facilitating immune cell to penetrate into the tumor parenchyma. Moreover, the TLSs induced by LIGHT maintain the proliferative competence of stem-like T cells. Concurrently, αCD3 also enhances the interaction between T cells and tumor cells, driving MHC-I-unrestricted cytolysis and blocking immune evasion. The all-round modulation significantly improves the T cell immunity, effectively inhibiting “immune-cold” solid tumors.

Additionally, ICB and CAR-T cell therapy are the most important immunotherapy approaches in current, but the performances are far from satisfactory yet. Although several ICIs have been clinically applied, about 80% of patients respond poorly or do not benefit from ICB therapy.^78^ CAR-T cell therapy is still difficult to apply to solid tumors, with the extremely low infiltration efficiency and its rapid inactivation and exhaustion in TME being the main factors. Increasing the injection dose is a possible solution, but high-dose CAR-T cell therapy leads to severe side effects such as CRS and ICANS. Our P_αCD3&LIGHT_ can address the series of issues of T cell immunity including recruitment, infiltration, proliferation, and activity maintenance, thus not only significantly improves the efficacy of ICIs but also enables low-dose CAR-T cells to exhibit outstanding effects, curing 40% of melanoma-bearing mice without obvious side effects. Notably, melanoma, colon carcinoma, and breast cancer are all typical “cold” tumors that are difficult to mobilize an immune response. Therefore, it can be speculated that our P_αCD3&LIGHT_ should have better efficacy for those tumors with strong immune responsivity. Moreover, the highly efficient immune modulation can be combined with chemotherapy or radiotherapy, underscoring the broad prospects of our P_αCD3&LIGHT_.

Clinical studies identified the significant correlations between LIGHT level and T cell infiltration, as well as between anti-CD3 concentration and T cell activation. Our primary results indicate that P_αCD3&LIGHT_ holds great promise in enhancing the efficacy of human immunotherapy. Future improvements can focus on the following aspects. (1) Microfluidics has emerged as a pivotal platform for the preparation of NPs in the nanomedicine field.^79^^,^^80^ This technology enables precise NP size control by adjusting the flow rate ratio and total flow rate, achieving ultralow polydispersity (PDI < 0.1).^81^^,^^82^ Especially, the helical-blade-strengthened co-flow focusing device achieves high-throughput synthesis of monodisperse ultrasmall nanoliposomes (<40 nm).^83^^,^^84^ Furthermore, microfluidics significantly enhances the EE of nucleic acids (e.g., mRNA, small interfering RN, and plasmids) through optimizing lipid composition and flow conditions.^85^ By leveraging parallelizable chip architectures, microfluidic systems facilitate seamless volumetric scale-up from microliter-to-liter scales, supporting large-scale production.^86^ Thus, microfluidics demonstrates compelling potential for industrial-scale clinical translation of plasmid-loaded NPs. In addition, the immunogenicity of NPs limits the levels and durability of expression of the encoded protein. Currently, phenol-containing ionizable liposomes effectively mitigate intracellular ROS, reducing cellular inflammatory responses, which enhances therapeutic efficacy while mitigating side effects.^87^ Consequently, the biosafety profiles of nanocarriers can be further optimized to advance clinical translation. (2) The optimal “therapeutic window” for the combination of P_αCD3&LIGHT_ with ICIs, CAR-T cells, and other treatments can be investigated to achieve the best therapeutic effect in clinical practice. (3) P_αCD3&LIGHT_ preferentially normalizes, rather than merely expands, the tumor blood and lymphatic vessels, which is critical for efficient immunocyte trafficking into tumors to create a localized immune-favorable niche.^88^^,^^89^ Moreover, while angiogenesis and lymphangiogenesis may facilitate metastasis, the synergistic effect of LIGHT and αCD3 rapidly establishes a potent, tumor-specific T cell response that likely eliminates circulating tumor cells before distant seeding occurs. It was worth mentioned that, for therapy-refractory advanced malignancies and metastasis-susceptible carcinomas (e.g., pancreatic ductal adenocarcinoma and small cell lung cancer), more rigorous monitoring and systematic characterization are needed to determine the potential pro-metastatic effect of P_αCD3&LIGHT_ in the future study. And synergistic integration of anti-angiogenic agents may counteract this detrimental sequela. (4) Clinical trials have shown that TLS is closely related to the prognosis of many tumors. Given that P_αCD3&LIGHT_ can effectively promote TLS formation in murine tumors, its role in inducing TLS neogenesis in human tumors deserves further investigation.

### Limitations of the study

Although our study demonstrated the comprehensive and effective modulation of T cell immunity by using P_αCD3&LIGHT_, some issues need to be taken into consideration in the future. Currently, viral vectors and synthetic nanocarriers serve as the predominant delivery approaches for *in vivo* gene therapy.^90^^,^^91^^,^^92^ While viral vectors achieve prolonged transgene expression, concomitant risks include elevated immunogenicity and off-target accumulation predominantly in the liver and spleen. The NP platform demonstrates superior delivery efficiency and attenuated immunogenicity compared to viral vectors. Notably, it enables precise tumor targeting through promoter optimization. However, long-term protein expression remains constrained with nanocarriers. Thus, therapeutic efficacy maintenance typically relies on periodic re-administration. In our study, intratumoral levels of functional proteins peaked at 48–72 h post-administration and subsequently declined gradually. To sustain therapeutic protein abundance, we implemented a repeated dosing regimen at 3-day intervals. Future studies should include detailed pharmacokinetic analyses to determine half-lives of αCD3 and LIGHT, enabling more accurate prediction of effective redosing schedules. On the other hand, αCD3, as an exogenous protein, may elicit the production of neutralizing antibodies, potentially preventing its binding to CD3 on the T cells. In general, frequent re-dosing regimens can accelerate the secretion of neutralizing antibodies by memory B cells. To resolve this issue, we can strategically reduce the dosing frequency without sacrificing efficacy. This can be achieved by enhancing intratumoral transfection efficiency—through improved delivery systems and engineered hybrid promoters—and by prolonging the protein retention via protease inhibition. We speculate that a weekly re-dosing interval may significantly mitigate the neutralization of αCD3, thereby restoring its efficacy and potentially expanding the clinical applicability of P_αCD3&LIGHT_. Secondly, pTERT has been extensively utilized in gene therapy in diverse tumor types, particularly in melanoma, gastrointestinal cancer, breast cancer, lung cancer, liver cancer, and pancreatic cancer.^93^^,^^94^^,^^95^ Nevertheless, the heterogeneity of pTERT activity across tumor types is undeniable, potentially yielding variable expression levels of functional proteins and compromising the efficacy of P_αCD3&LIGHT_. This problem can be addressed by using other tumor-specific promoters (TSPs) in certain tumors, such as kinase domain insert containing receptor promoter and survivin (Sur) promoter (high activity in gastrointestinal cancer, lung cancer, liver cancer, and breast cancer), human epidermal growth factor receptor promoter and rad51 recombinase (Rad51) promoter (high activity in breast cancer and pancreatic cancer), cyclooxygenase-2 promoter and urokinase-type plasminogen activator receptor promoter (high activity in colorectal cancer and breast cancer), and prostate-specific antigen promoter and probasin promoter (high activity in prostate cancer).^96^^,^^97^ Moreover, the heterogeneity of promoter in different tumors may be partially alleviated by promoter remold, such as optimization and modification, so as to enhance the efficiency of TSPs across tumor types.^97^ Thirdly, we conducted clinical relevance study of LIGHT and αCD3, and employed B16 tumor cells with human CD19 antigen (hCD19-B16) and murine T cells with anti-human CD19 scFv (hαCD19-mCAR-T cells) to simulate clinical practice as far as possible. However, since the function of LIGHT relies heavily on stromal cells, the reconstruction of the human tumor ECM in murine models should be taken into accounts when this technical challenge is overcome.

## Resource availability

### Lead contact

Further information and requests for resources and reagents should be directed to and will be fulfilled by the lead contact, Hai-Yan Xie (hyanxie@bjmu.edu.cn).

### Materials availability

Plasmid requests can be directed to the lead contact.

### Data and code availability

• All other data are available in the main text or in the supplemental information.
• The published article does not report custom computer code.
• Any additional information required to reanalyze the data reported in this paper is available from the lead contact upon request.

## Acknowledgments

We thank Prof. Zhen Gu and Prof. Jie Sun (Zhejiang University) for providing anti-human CD19 scFv CAR-T cell transgenic C57BL/6 mice and hCD19-B16 cells, Prof. Peng Jiang (Wuhan University) for providing human CAR-T cells, and Xin-He Yu (Central China Normal University) for helpful discussion. We thank the State Key Laboratory of Natural and Biomimetic Drugs (Peking University) and the Biological & Medical Engineering Core Facilities (Beijing Institute of Technology) for providing advanced types of equipment. This work was supported by the 10.13039/501100014219National Science Fund for Distinguished Young Scholars (22025401), the 10.13039/501100001809National Natural Science Foundation of China (22293034, 22293030, and 32101140), the Beijing Natural Science Foundation (L242109), the 10.13039/501100007129Natural Science Foundation of Shandong Province (ZR2024QC324), and the 10.13039/501100005085Beijing Institute of Technology Research Fund Program for Young Scholars (XSQD-202212002).

## Author contributions

Conceptualization, J.H., W.N., and H.-Y.X.; methodology, J.H., C.Z., C. Liang, and W.X.; investigation, J.H., Y. Li., L.D., C. Liu, W.-R.Z., X.M., R.C., and Y. Lei; analysis and visualization, all authors; writing – original draft, J.H., C.Z., W.N., and H.-Y.X.; writing – review and editing, J.H., W.N., and H.-Y.X.; funding acquisition, W.N. and H.-Y.X.; supervision, H.-Y.X.

## Declaration of interests

The authors declare no competing interests.

## STAR★Methods

### Key resources table

**Table:** 

REAGENT or RESOURCE	SOURCE	IDENTIFIER
**Antibodies**		

Mouse Anti-HA-Tag Monoclonal Antibody	Proteintech	Cat# 66006-2-Ig; RRID: AB_2881490
Mouse Anti-His-Tag Monoclonal Antibody	Proteintech	Cat# 66005-1-Ig; RRID: AB_11232599
Rabbit Anti-eGFP Polyclonal Antibody	Abcam	Cat# ab6556
Mouse Anti-Beta Actin Monoclonal antibody	Proteintech	Cat# 66009-1-Ig; RRID: AB_2687938
HRP-conjugated Goat Anti-Mouse IgG(H + L)	Proteintech	Cat# SA00001-1; RRID: AB_2722565
HRP-conjugated Goat Anti-Rabbit IgG(H + L)	Abcam	Cat# ab6721
APC anti-HA.11 Epitope Tag Antibody	BioLegend	Cat# 901523; RRID: AB_2734657
Alexa Fluor® 488 anti-His Tag Antibody	BioLegend	Cat# 652509; RRID: AB_2716151
Alexa Fluor® 488 anti-mouse CD31 (PECAM-1) Antibody	Biolegend	Cat# 160207; RRID: AB_2904304
Biotin Anti-mouse/human PNAd Antibody	Biolegend	Cat# 120803; RRID: AB_493556
Rabbit Anti-TNFSF14 (LIGHT) antibody	Bioss	Cat# bs-2462R; RRID: AB_10854746
Goat Anti-Rabbit IgG H&L (Alexa Fluor® 647)	Abcam	Cat# ab150079; RRID: AB_2722623
FITC anti-mouse CD45 Antibody	Biolegend	Cat# 157214; RRID: AB_2894427
APC anti-mouse CD3 Antibody	Biolegend	Cat# 100235; RRID: AB_2561455
APC anti-mouse/human CD45R/B220 Antibody	Biolegend	Cat# 103211; RRID: AB_312996
APC anti-mouse F4/80 Antibody	Biolegend	Cat# 123116; RRID: AB_893481
APC anti-mouse CD49b (pan-NK cells) Antibody	Biolegend	Cat# 108910; RRID: AB_313416
PE anti-mouse CD11c Antibody	Biolegend	Cat# 117308; RRID: AB_313776
APC anti-mouse CD45 Antibody	Biolegend	Cat# 147707; RRID: AB_2563540
FITC anti-mouse CD4 Antibody	Biolegend	Cat# 100405; RRID: AB_312690
PE anti-mouse/rat/human FOXP3 Antibody	Biolegend	Cat# 320007; RRID: AB_492980
PE anti-mouse CD3 Antibody	Biolegend	Cat# 100205; RRID: AB_312662
FITC anti-mouse CD8a Antibody	Biolegend	Cat# 162313; RRID: AB_3097398
FITC anti-mouse/human CD45R/B220 Antibody	Biolegend	Cat# 103205; RRID: AB_312990
FITC anti-HA.11 Epitope Tag Antibody	Biolegend	Cat# 901507; RRID: AB_2565058
APC anti-His Tag Antibody	Biolegend	Cat# 362605; RRID: AB_2715818
Rabbit Anti-Melanoma gp100 Monoclonal Antibody	Abcam	Cat# ab137078; RRID: AB_2732921
Goat Anti-Rabbit IgG H&L (Alexa Fluor® 488)	Abcam	Cat# ab150077; RRID: AB_2630356
Rabbit Anti-TCF1/TCF7 Monoclonal Antibody	Abcam	Cat# ab314082
Goat Anti-Rabbit IgG H&L (Alexa Fluor® 594)	Abcam	Cat# ab150080; RRID: AB_2650602
Rabbit Anti-CXCL13 Recombinant Multiclonal Antibody	Proteintech	Cat# 86564-1-RR
APC anti-mouse Ki-67 Antibody	Biolegend	Cat# 652406; RRID: AB_2561929
PE anti-mouse CD69 Antibody	Biolegend	Cat# 104507; RRID: AB_313110
Goat Anti-Rabbit IgG H&L (Alexa Fluor® 405)	Abcam	Cat# ab175652; RRID: AB_2687498
PE anti-human CD19	Biolegend	Cat# 982402; RRID: AB_2616905
FITC-labeled Human CD19 (20–291) Protein	ACRO Biosystems	Cat# CD9-HF2H2
PE anti-human CD69	Biolegend	Cat# 985202; RRID: AB_2924641
Rabbit Anti-mouse CD11b Polyclonal Antibody	Servicebio	Cat# GB115689
HRP-conjugated Goat Anti-Rabbit IgG	Servicebio	Cat# GB23303; RRID: AB_2811189
InVivoMAb Anti-mouse PD-L1 (B7-H1)	Bio X Cell	Cat# BE0101; RRID: AB_10949073
InVivoPlus Anti-mouse CTLA-4 (CD152)	Bio X Cell	Cat# BP0164; RRID: AB_10949609
FITC anti-human CD3 Antibody	Biolegend	Cat# 317305; RRID: AB_571906
PE anti-human CD8a Antibody	Biolegend	Cat# 388703; RRID: AB_3068248
FITC anti-human CD8a Antibody	Biolegend	Cat# 300905; RRID: AB_314109
PE anti-human CD258 (LIGHT) Antibody	Biolegend	Cat# 318706; RRID: AB_830864

**Biological samples**		

Heart	C57BL/6 mice in this study	N/A
Liver	C57BL/6 mice in this study	N/A
Spleen	C57BL/6 mice in this study	N/A
Lung	C57BL/6 mice in this study	N/A
Kidney	C57BL/6 mice in this study	N/A
Lymph node	C57BL/6 mice in this study	N/A
Serum	C57BL/6 mice in this study	N/A
B16 melanoma	C57BL/6 mice in this study	N/A
CT26 colon carcinoma	BALB/c mice in this study	N/A
4T1 breast cancer	BALB/c mice in this study	N/A
Burkitt’s lymphoma	Nude mice in this study	N/A
Human colorectal cancer	Qian’an Yanshan Hospital	N/A
Human nasopharyngeal carcinoma	Qian’an Yanshan Hospital	N/A
Human cervical cancer	Qian’an Yanshan Hospital	N/A
Mouse lung fibroblasts	C57BL/6 mice in this study	N/A
Proximal tubular epithelial cells	C57BL/6 mice in this study	N/A
CD8^+^ T lymphocytes	C57BL/6 mice in this study	N/A
Bone marrow-derived dendritic cells	C57BL/6 mice in this study	N/A
Hematopoietic progenitor cells	C57BL/6 mice in this study	N/A
Mouse cancer-associated fibroblasts	C57BL/6 mice in this study	N/A
Human cancer-associated fibroblasts	Human gastric cancer tissues	N/A
OT-1 T lymphocytes	OT-1 transgenic C57BL/6 mice	N/A
Murine CAR-T cells expressing anti-human CD19 scFv	Transgenic C57BL/6 mice	N/A
Human CAR-T cells expressing anti-human CD19 scFv	This study	N/A

**Chemicals, peptides, and recombinant proteins**		

D-Luciferin	APExBIO	B6040
Triton X-100	Solarbio	T8200
Hoechst 33342	Thermo	62249
CFDA-SE	Invitrogen	V12883
Fluoroshield™ with DAPI	Sigma-Aldrich	F6057
PE Streptavidin	Biolegend	405203
Propidium Iodide (PI)	HARVEYBIO	SR3543
Picro-Sirius Red (PS-Red) solution	Servicebio	G1018
CytoTrace Orange CMTMR	AAT Bioquest	22014
5-Tamra-LPETG	Guoping Pharmaceutical	Customized
5-Tamra-G_5_C-DBCO	Guoping Pharmaceutical	Customized
Biotin-Ahx-LPETGS-NH_2_	Guoping Pharmaceutical	Customized
DiR	Invitrogen	D12731
DOTAP	Xi’an Qiyue Biotechnology	132172-61-3
PEG5k-PLGA11k	Xi’an Ruixi Biotechnology	Customized
Mal-(PEG)_4_-NHS Ester	MedChemExpress	1325208-25-0
Traut’s Reagent	Thermo	4781-83-3
CB-AC_3_ManNAz	Xi’an Qiyue Biotechnology	Customized
DBCO-G_5_	Guoping Pharmaceutical	Customized
Purified anti-mouse CD3 Antibody	BioLegend	Cat# 100202; RRID: AB_312658
Purified anti-mouse CD28 Antibody	BioLegend	Cat# 102102; RRID: AB_312866
Animal-Free Recombinant Murine IL-2 Protein	PeproTech	Cat# AF-212-12
Animal-Free Recombinant Murine IL-4 Protein	PeproTech	Cat# AF-214-14
Animal-Free Recombinant Murine IL-21 Protein	PeproTech	Cat# AF-210-21
Animal-Free Recombinant Murine GM-CSF	PeproTech	Cat# AF-315-03
Human IL-2 Recombinant Protein	PeproTech	Cat# 200-02
Purified anti-human CD3 Antibody	BioLegend	Cat# 317301; RRID: AB_571926
Purified anti-human CD28 Antibody	BioLegend	Cat# 302901; RRID: AB_314303

**Critical commercial assays**		

Quant-iT™ PicoGreen® dsDNA Reagent Kit	Invitrogen	P11496
MojoSort™ Mouse CD8^+^ T cell Isolation Kit	BioLegend	480129
MojoSort™ Mouse Pan B Cell Isolation Kit	BioLegend	480052
EasySep™ Mouse Hematopoietic Progenitor Cell Isolation Kit	STEMCELL	19856
Mouse Tumor-Associated Fibroblast Isolation Kit	Miltenyi Biotech	130-116-474
Mouse Tumor Dissociation Kit	Miltenyi Biotech	130-096-730
Mouse Anti-CD3 Antibody ELISA Kit	Shanghai Yanjin	F08515
Mouse LIGHT ELISA Kit	Shanghai Yanjin Biotechnology	F16227
Mouse Vascular endothelial growth factor C (VEGF-C) ELISA Kit	FineTest	EM0217
Mouse Mucosal Vascular Addressin Cell Adhesion Molecule-1 (MADCAM-1) ELISA Kit	RayBiotech	ELM-MAdCAM1
Mouse Glycosylation-Dependent Cell Adhesion Molecule 1 (GLYCAM-1) ELISA Kit	MyBioSource	MBS288240
Mouse soluble vasccular cell adhesion molecule 1 (sVCAM-1) ELISA Kit	FineTest	EM1382
Mouse Rantes (CCL-5) ELISA Kit	FineTest	EM0164
Mouse MIP-3 Beta (CCL-19) ELISA Kit	FineTest	EM1211
Mouse SLC (CCL-21)ELISA Kit	FineTest	EM1365
Mouse SDF-1 (CXCL-12) ELISA Kit	FineTest	EM0174
Mouse BLC (CXCL-13) ELISA Kit	FineTest	EM0873
Mouse Granulocyte-Macrophage Colony Stimulating Factor (GM-CSF) ELISA Kit	FineTest	EM0089
Mouse Transforming Growth Factor β 1 (TGF-β1) ELISA Kit	FineTest	EM0176
Mouse Matrix Metalloproteinase 1 (MMP-1) ELISA Kit	FineTest	EM1213
Mouse Matrix Metalloproteinase 9 (MMP-9) ELISA Kit	FineTest	EM0144
Mouse Matrix Metalloproteinase 25 (MMP-25) ELISA Kit	MyBioSource	MBS2905135
Mouse Hydroxyproline (Hyp) ELISA Kit	CUSABIO	CSB-E08839m
Mouse Collagen Type I (COL1) ELISA Kit	FineTest	EM0939
Mouse Interferon-gamma (IFN-γ) ELISA Kit	BioLegend	430807
Mouse Tumor Necrosis Factor-alpha (TNF-α) ELISA Kit	BioLegend	430907
Mouse Granzyme B (Gzm-B) ELISA Kit	Jonln	JL11913
Human Anti-CD3 Antibody Elisa Kit	ACRO Biosystems	CRS-A015
Human LIGHT Elisa Kit	FineTest	EH0316
Human Interferon-gamma (IFN-γ) ELISA Kit	Bio-Techne	QK285
Human Tumor Necrosis Factor-alpha (TNF-α) ELISA Kit	Jonln	JL10208
Human Granzyme B (Gzm-B) ELISA Kit	Jonln	JL10402
Mouse Serum Amyloid A (SAA) ELISA Kit	FineTest	EM0169
Mouse Interleukin-6 (IL-6) ELISA Kit	BioLegend	431307
Mouse Interleukin-1 Beta (IL-1β) ELISA Kit	Cell Applications	CL0394
CellEvent™ Caspase-3/7 Green Flow Cytometry Kit	Invitrogen	C10427
Hematoxylin-Eosin (H&E) HD Constant Dye Kit	Servicebio	G1076
ProteoPrep® Total Extraction Sample Kit	Millipore	12352200
TransZol Up Plus RNA Kit	Transgen	ER501-01-V2
TransScript® Uni All-in-One First-Strand cDNA Synthesis SuperMix for qPCR	Transgen	AU341-02
PerfectStart® Green qPCR SuperMix	Transgen	AQ602-01
Tumor-Infiltrating Tissue Lymphocyte Separation Solution Kit	Solarbio	P9000
Mouse Peripheral Blood Lymphocyte Isolation Solution Kit	Solarbio	P8620
Mouse Spleen Lymphocyte Isolation Solution Kit	Solarbio	P8860
HisPur™ Ni-NTA Spin Purification Kit	Thermo	88229
BCA Assay Kit	Thermo	23225
Cell Counting Kit-8 (CCK-8)	Dojindo	LQ683
True-Nuclear™ Transcription Factor Buffer Set	BioLegend	424401

**Experimental models: Cell lines**		

B16-OVA	Shanghai Aoyin	SAc0040OG
4T1	Cell Bank of the Chinese Academy of Science	TCM32
CT26	Cell Bank of the Chinese Academy of Science	TCM37
MC38	Cell Bank of the Chinese Academy of Science	SCSP-5431
C166	Shanghai Yubo	AE-1177
Raji	Cell Bank of the Chinese Academy of Science	TCHu 44
Human umbilical vein endothelial cells	Shanghai Qida	CD0290

**Experimental models: Organisms/strains**		

C57BL/6 mice	Peking University Health Science Center	N/A
BALB/c mice	Peking University Health Science Center	N/A
Nude mice	Peking University Health Science Center	N/A

**Oligonucleotides**		

Primers for qPCR, see Table S1	Tsingke	N/A

**Software and algorithms**		

ImageJ	National Institutes of Health	https://imagej.nih.gov/ij/
Microsoft office	Microsoft	https://www.microsoft.com/zh
GraphPad Prism 10.0	GraphPad	http://www.graphpad-prism.cn/
Imaris 9.0	Bitplane	https://imaris.oxinst.com

### Experimental model and study participant details

#### Experimental mouse models

All mice were purchased from Peking University Health Science Center (PKUHSC) Department of Laboratory Animal Science (Beijing, China). All mice were housed at a temperature of 20°C–25°C, with humidity of 30–70% and under a 12 h light/12 h dark cycle. Feed and water were available. All mice received human care according to the animal care regulations of the Beijing Animal Ethics Association and the Ethics Committee of Beijing Institute of Technology (Approval ID: 2019-0010-*M*-2020019). The melanoma-bearing mouse model was established by subcutaneously inoculating 1 × 10^6^ B16-OVA cells into the right flank of 4-week-old C57BL/6 mice (female). The colon carcinoma- or breast cancer-bearing mouse model was established by subcutaneously inoculating 1 × 10^6^ CT26 cells or 1 × 10^6^ 4T1 cells into the right flank of 4-week-old BALB/c mice (female). When the volume of tumors reached 50 mm^3^, P_αCD3&LIGHT_ and other control plasmids were intravenously injected into mice every three days for four consecutive administrations (30 μg each time). The ICIs, αPD-L1 and αCTLA-4, were administered intravenously on the day following the plasmid injections (30 μg each time). The B-cell lymphoma-bearing mouse model was established by subcutaneously inoculating 1 × 10^6^ Raji cells into right flank of 4-week-old nude mice (female). When the volume of tumors reached 50 mm^3^, the mice were intravenously injected with either PBS or hP_αCD3&LIGHT_ every three days for four consecutive administrations (30 μg each time). 48 h after the last injection, the mice were sacrificed. The tumor model was established by subcutaneously inoculating 1 × 10^6^ hCD19-B16 cells into the right flank of 4-week-old C57BL/6 mice (female). Then, the mice were intravenously injected with either PBS or P_αCD3&LIGHT_ every three days for four consecutive administrations (30 μg each time). On the 12th and 18th day, mice were intravenously injected with hαCD19-mCAR-T cells. The mice were euthanized on the day 23. Serum, tumor tissues, and normal organs were collected for further analysis.

#### Cell lines

B16-OVA, 4T1, CT26, MC38 tumor cells and C166 mouse vascular endothelial cells were cultured in DMEM supplemented with 10% FBS and 1% penicillin-streptomycin (DMEM complete medium). Raji cells were cultured in RPMI 1640 medium supplemented with 10% FBS and 1% penicillin-streptomycin (1640 complete medium). Human umbilical vein endothelial cells (HUVECs) were cultured in ECM. All cells were maintained in a 37°C humidified chamber with 5% CO_2_. All cell lines employed in this study were authenticated using short tandem repeat (STR) profiling at initiation of the study. All cell lines used in this study routinely tested negative for mycoplasma contamination in PCR-based assays.

#### Mouse primary cells

Mouse lung fibroblasts (MLFs) were derived from explants of finely minced lung tissues from 8-week-old C57BL/6 mice and then cultured in DMEM/F-12 medium supplemented with 15% FBS, 1.0% penicillin-streptomycin and 1.0% L-glutaMAX-1.^98^ Proximal tubular epithelial cells (PTECs) were isolated from the kidneys of 4-week-old C57BL/6 mice and subsequently cultured in DMEM/F-12 medium supplemented with 10% FBS and 1% penicillin-streptomycin.^99^ CD8^+^ T lymphocytes (CD8^+^ T cells) were sorted from the spleens of 6-week-old C57BL/6 mice by using a mojoSort mouse CD8^+^ T cell isolation kit in accordance with the manufacturer’s instructions. The cells were cultured in RPMI 1640 (ATCC modification) complete medium containing 1% L-glutaMAX-1, 1% NEAA, 0.1% β-mercaptoethanol, 20 ng/mL murine IL-2, 0.25 μg/mL anti-mouse CD3 antibody and 0.25 μg/mL anti-mouse CD28 antibody. Pan B cells were sorted from the spleens of 6-week-old C57BL/6 mice by using a mojoSort mouse Pan B cell isolation kit in accordance with the manufacturer’s instructions. The obtained cells were cultured in RPMI 1640 (ATCC modification) complete medium containing 0.1% β-mercaptoethanol, 10 mM HEPES, 1 mM sodium pyruvate, 1% NEAA, 2 ng/mL murine IL-4 and 10 ng/mL murine IL-21. Bone marrow-derived dendritic cells (BMDCs) were extracted from the hindlimb bones of 4-week-old C57BL/6 mice and induced using RPMI 1640 (ATCC modification) complete medium containing 20 ng/mL murine GM-CSF and 20 ng/mL murine IL-4. Hematopoietic progenitor cells (HPCs) were isolated from the bone marrow of 6-week-old C57BL/6 mice by using a EasySep Mouse Hematopoietic Progenitor Cell Isolation Kit, and subsequently maintained in StemSpan SFEM II medium. Mouse cancer-associated fibroblasts (CAFs) were isolated from B16-OVA subcutaneous tumors by using a mouse tumor-associated fibroblast isolation kit and mouse tumor dissociation kit in accordance with the manufacturer’s instructions. The isolated CAFs were cultured in DMEM supplemented with 30% FBS and 1% penicillin-streptomycin.

#### Human primary cells

Human cancer-associated fibroblasts (hCAFs) were isolated from gastric cancer tissues obtained from Beijing Cancer Hospital (Beijing, China). The tissues were minced into 1–3 mm pieces and placed into a T75 tissue culture flask containing DMEM medium supplemented with 30% FBS and 1% penicillin-streptomycin. The passage number of all primary cells was limited to between 3 and 8. All cells were maintained in a 37°C humidified chamber with 5% CO_2_. The tumor specimens were obtained from resected clinical tumors. This study was approved by the medical ethics committee of Peking University Cancer Hospital (No. 2019YZJ38). The patients gave informed consent for collection of clinical information, tissue collection and research testing.

#### Genetically engineered cells

OT-1 T lymphocytes (OT-1) and murine CAR-T cells expressing anti-human CD19 scFv (hαCD19-mCAR-T) were sorted from the spleens of 6-week-old OT-1 transgenic C57BL/6 mice purchased from Cyagen Biosciences and 8-week-old transgenic C57BL/6 mice gifted from Prof. Zhen Gu of Zhejiang University, respectively. The obtained cells were cultured in RPMI 1640 (ATCC modification) complete medium containing 1% L-glutaMAX-1, 1% NEAA, 0.1% β-mercaptoethanol, 20 ng/mL murine IL-2, 0.25 μg/mL anti-mouse CD3 antibody and 0.25 μg/mL anti-mouse CD28 antibody. Human CAR-T cells expressing anti-human CD19 scFv (hαCD19-hCAR-T) were gifted from Prof. Peng Jiang of Wuhan University and cultured in RPMI 1640 (ATCC modification) complete medium containing 1% L-glutaMAX-1, 1% NEAA, 0.1% β-mercaptoethanol, 20 ng/mL human IL-2, 0.25 μg/mL anti-human CD3 antibody and 0.25 μg/mL anti-human CD28 antibody. The passage number of above cells was limited to between 3 and 8. B16 cells expressing human CD19 antigen (hCD19-B16) were gifted from Prof. Jie Sun of Zhejiang University. All cells were maintained in a 37°C humidified chamber with 5% CO_2_.

#### Human data and samples

*LIGHT* RNA-seq expression data (log_2_-transformed TPM values) and clinical survival metadata were retrieved from TCGA pan-cancer cohorts. The data were stratified into two groups based on the median expression level of LIGHT, and Kaplan-Meier curves with log rank tests were generated to compare survival outcomes between high- and low-expression groups. The sample sizes were 50 (breast invasive carcinoma), 283 (cervical squamous cell carcinoma and endocervical adenocarcinoma), 486 (colon adenocarcinoma), 88 (rectum adenocarcinoma), 175 (uterine corpus endometrial carcinoma), 141 (glioblastoma multiforme), 492 (head and neck squamous cell carcinoma), 231 (liver hepatocellular carcinoma), 105 (lung adenocarcinoma), 68 (lung squamous cell carcinoma), 98 (skin melanoma), 81 (ovary serous cystadenocarcinoma), 480 (prostate adenocarcinoma), 64 (kidney chromophobe), 346 (stomach adenocarcinoma), 133 (testicular germ cell tumor), 495 (thyroid carcinoma), and 169 (bladder urothelial carcinoma).

All tumor specimens were obtained from resected clinical tumors (*n* = 30). This study was approved by the medical ethics committee of Peking University Cancer Hospital (No. 2019YZJ38). All patients gave informed consent for collection of clinical information, tissue collection and research testing. Detailed information of tumor samples was summarized in Table S2. And no statistically significant gender-based effects were observed.

### Method details

#### Plasmid construction and physicochemical characterization of nanoparticles

The DNA sequences of telomerase reverse transcriptase promoter (pTERT), and mouse and human anti-CD3 scFv (αCD3) were obtained from previous studies.^100^^,^^101^^,^^102^ The DNA sequences of mouse and human LIGHT were obtained from the NCBI GenBank database (NM_019418.4 and NM_001376887). In all plasmids, the pTERT region was immediately upstream of the coding sequence. The P_Ctrl_ or hP_Ctrl_ plasmid only contained pTERT. Within the P_αCD3_ or hP_αCD3_ plasmid, the IgK signal peptide and HA-tag sequences were fused at the 5′-terminal of mouse or human αCD3, while B7 transmembrane peptide sequence was strategically positioned at the 3′-terminal (IgK-HA-αCD3-B7). For the P_LIGHT_ or hP_LIGHT_ plasmid, the His-Tag sequence was appended to the 3′-terminal of mouse or human LIGHT (LIGHT-His). In P_αCD3&LIGHT_ or hP_αCD3&LIGHT_ plasmid, a P2A self-cleaving peptide gene was applied to bridge IgK-HA-αCD3-B7 (5′-terminal) and LIGHT-His (3′-terminal). Furthermore, the P_αCD3&LIGHT&eGFP_ or hP_αCD3&LIGHT&eGFP_ plasmid was constructed by inserting the T2A self-cleaving peptide and eGFP reporter sequences at the 3′-terminal of P_LIGHT_ or hP_LIGHT_ plasmid. The P_Luc_ plasmid was constructed by inserting the luciferase gene into the C-terminus of pTERT.

All plasmids were encapsulated with DOTAP and PEG5k-PLGA11k nanoparticles using a double emulsion method.^103^ The nanoparticles (NPs) were negatively stained with phosphotungstic acid (PTA) and subsequently observed by a transmission electron microscopy (TF20, FEI) to characterize their shape and surface morphology. The diameter, polydispersity index (PDI), and zeta potential of NPs were characterized by a nanoparticle size and zeta potential analyzer (NanoBrook 90plus PALS, Brookhaven). To assess stability, NPs were incubated in DMEM medium, and their hydrodynamic diameter and PDI were analyzed within 5 days. The encapsulation efficiency (EE) and drug loading capacity (DLC) of plasmid were assessed by dissolving 5 mg of NPs in 1 mL of DMSO at room temperature and quantifying released plasmid via PicoGreen assay using a fluorescence microplate reader (Varioskan LUX, Thermo). Samples and standards (same plasmid batch) were diluted in Tris-EDTA (pH 8.0) within the kit’s range (250 pg/mL-2 μg/mL), with blank NPs for background control. Next, the toxicity of NPs on red blood cells (RBCs) was assessed. Fresh blood was collected from C57BL/6 mice, and RBCs were isolated by centrifugation (300 × g, 5 min). Then, RBCs were diluted to a final concentration of 2% (v/v) with PBS and incubated with 2 μg of P_αCD3&LIGHT_ or other controls. And the 0.1% Triton X-100 and PBS were set as positive control and negative control, respectively. A quantitative measurement of hemolysis was performed by spectrophotometric analysis at 415 nm. The hemolysis rate was calculated according to the following formula: Hemolysis rate (%) = (A_Sample_-A_Negative_)/(A_Positive_-A_Negative_) × 100%.

#### Tumor-specific analysis of plasmid

B16-OVA cells were seeded in 12-well plates at a density of 3 × 10^5^ cells per well and then incubated with Opti-MEM medium containing 2 μg of PBS, P_Ctrl_, P_αCD3_, P_LIGHT_, or P_αCD3&LIGHT_ for 4 h. To determine whether P_αCD3&LIGHT_ could drive the co-expression of αCD3 and LIGHT specifically in tumor cells *in vitro*, MLFs, PTECs, CTLs, Pan B cells, BMDCs, HPCs, and B16-OVA cells were seeded in 12-well plates and left to incubate with Opti-MEM medium containing either PBS or 2 μg of P_αCD3&LIGHT&eGFP_ for 4 h. At 72 h post-transfection, the eGFP-positive cells were detected using a fluorescence microscope (Eclipse-Ti2, Nikon) and quantified by a flow cytometer (Bioscience FACSAria, BD). Meanwhile, RNA was extracted using a TransZol Up Plus RNA Kit and then reverse-transcribed to cDNA using a cDNA synthesis kit. The relative expression levels of αCD3 and LIGHT were analyzed by qPCR using a SuperMix (CFX96, Bio-Rad). The 2^−ΔΔCT^ method was used to calculate the fold change in gene expression. Proteins were extracted with a protein extraction kit. Following this, the expression levels of αCD3, LIGHT and eGFP were analyzed by western blotting (Primary antibody: Mouse Anti-HA-Tag Monoclonal Antibody (1:10000, 4°C, overnight), Mouse Anti-His-Tag Monoclonal Antibody (1:5000, 4°C, overnight), Rabbit Anti-eGFP polyclonal Antibody (1:4000, 4°C, overnight) or Mouse Anti-Beta Actin Monoclonal antibody (1:10000, 4°C, overnight); secondary antibody: HRP-conjugated Goat Anti-Mouse IgG(H + L) (1:10000, RT, 120 min) or HRP-conjugated Goat Anti-Rabbit IgG(H + L) (1:10000, RT, 120 min)). The proteins of αCD3 and LIGHT were quantified by ELISA in accordance with the manufacturer’s instructions. To evaluate the *in vivo* biodistribution of NPs, a melanoma-bearing mouse model was established by subcutaneously inoculating 1 × 10^6^ B16-OVA cells into the right flank of 4-week-old C57BL/6 mice. The Cy5-labeled NPs were then administered intravenously into mice at a dose of 2 mg/kg. 48 h later, the melanoma and normal organs (heart, liver, spleen, lung, kidney, lymph node, brain, stomach, intestine, and bone marrow) were collected and analyzed using an IVIS spectrum imaging system (IVIS SPECTRUM, PerkinElmer). To determine whether DOTAP-PEG-PLGA NPs penetrate deeply into the tumor parenchyma, the frozen sections of tumor tissues were observed using super-resolution laser confocal microscopy, and then three-dimensional images were reconstructed using Imaris 9.0 software. To evaluate the tumor specificity of P_αCD3&LIGHT_ *in vivo*, melanoma-bearing mice were intravenously injected with 30 μg of P_Luc_. At 48 h post injection, the mice received an intraperitoneal administration of D-luciferin (150 mg/kg), followed by bioluminescence imaging using an IVIS spectrum imaging system (IVIS SPECTRUM, PerkinElmer) 15 min later. In addition, the mice were intravenously injected with either PBS or P_αCD3&LIGHT&eGFP_ every three days for four consecutive administrations (30 μg each time). At 48 h post final injection, the melanoma and normal organs were isolated to detect the eGFP-positive cells by immunofluorescence assay (VS200, Olympus) and flow cytometry, or were lysed to analyze the expression levels of αCD3 and LIGHT in different tissues by qPCR and ELISA.

In order to better cater to the clinical application of CAR-T, the human co-expression plasmid (hP_αCD3&LIGHT_) and other controls were constructed. After 3 × 10^5^ Raji cells were transfected with 2 μg of PBS, hP_Ctrl_, hP_αCD3_, hP_LIGHT_ or hP_αCD3&LIGHT_, the expression levels of hαCD3 and hLIGHT were assessed using qPCR, ELISA and western blotting (Primary antibody: Mouse Anti-HA-Tag Monoclonal Antibody (1:10000, 4°C, overnight), Mouse Anti-His-Tag Monoclonal Antibody (1:5000, 4°C, overnight) or Mouse Anti-Beta Actin Monoclonal antibody (1:10000, 4°C, overnight); secondary antibody: HRP-conjugated Goat Anti-Mouse IgG(H + L) (1:10000, RT, 120 min)). Raji cells were transfected with either PBS or 2 μg of hP_αCD3&LIGHT&eGFP_. The eGFP-positive Raji cells were observed using a confocal microscopy and counted by flow cytometry. For CLSM imaging, cell membrane and nucleus were labeled with PE anti-human CD19 (1:100, 4°C, 60 min) and Hoechst 33342 (1:1000, RT, 15 min), respectively. Then, for analysis of the tumor specificity of hP_αCD3&LIGHT_ *in vivo*, a B-cell lymphoma-bearing mouse model was established by subcutaneously inoculating 1 × 10^6^ Raji cells into right flank of 4-week-old nude mice. When the volume of tumors reached 50 mm^3^, the mice were intravenously injected with either PBS or hP_αCD3&LIGHT_ every three days for four consecutive administrations (30 μg each time). 48 h after the last injection, the B-cell lymphoma were isolated and lysed to determine the concentrations of hαCD3 and hLIGHT in different tissues by ELISA.

#### Function analysis of LIGHT

##### Chemokines, adhesion molecules and high endothelial venules

B16-OVA cells were seeded in 24-well plates at a density of 1 × 10^5^ cells per well and then incubated with Opti-MEM medium containing 1 μg of PBS, P_Ctrl_, P_αCD3_, P_LIGHT_, or P_αCD3&LIGHT_ for 4 h. After that, transfected cells were incubated with 1 × 10^5^ CAFs or 1 × 10^5^ C166 cells, respectively. 60 h later, the cells were collected to analyze mRNA expression levels of *p100*, chemokines, and adhesion molecules by qPCR. The functions of chemokines and adhesion molecules were analyzed by tracking chemotaxis of immune cells in a transwell migration assay, in which 2 × 10^5^ B16-OVA cells transfected with different plasmids were incubated with 1 × 10^5^ CAFs and 1 × 10^5^ C166 cells in the lower chamber of transwell plates. 60 h later, 2 × 10^6^ CFDA-SE-labeled (5 μM, 37°C, 15 min) OT-1 cells were cultured in the upper chamber with a pore size of 5 μm, and 2 × 10^6^ CFDA-SE-labeled Pan B cells and 2 × 10^6^ CFDA-SE-labeled BMDCs were cultured in the upper chamber with a pore size of 8 μm, respectively. The migration of three cells was observed by a confocal microscopy. 6 h later, the cells recruited to the lower chamber were carefully suspended and then counted using an automated cell counter (Scepter 3.0, Millipore). For human CAR-T assay, Raji cells were seeded in 24-well plates at a density of 1 × 10^5^ cells per well and then incubated with Opti-MEM medium containing 1 μg of PBS, hP_Ctrl_, hP_αCD3_, hP_LIGHT_ or hP_αCD3&LIGHT_ for 4 h. After that, transfected cells were incubated with 1 × 10^5^ hCAFs or HUVECs. After 60 h, the cells were collected to analyze mRNA levels of chemokines and adhesion molecules by qPCR. To test the recruitment of human CAR-T, 2 × 10^5^ Raji cells transfected with different plasmids were incubated with 1 × 10^5^ hCAFs and 1 × 10^5^ HUVECs in the lower chamber of transwell plates. After 60 h, 2 × 10^6^ CFDA-SE-labeled hCAR-T cells were cultured in the upper chamber with a pore size of 8 μm. 6 h later, hCAR-T cells in the lower chamber were counted.

Next, the secretions of chemokines and adhesion molecules in tumors mediated by different plasmids were assessed. The melanoma-bearing mouse model and administration route were the same as in Chapter “Tumor-specific analysis of plasmid”. The RNA in tumor tissues was extracted using a TransZol Up Plus RNA Kit and then reverse-transcribed into cDNA using a cDNA synthesis kit. The mRNA levels of chemokines and adhesion molecules were detected using qPCR. The tumor tissues were thoroughly ground to obtain the homogenate using a high-speed tissue grinding machine (KZ-II, Shanghai Jingxin). The concentrations of VEGF-C, MADCAM-1, GLYCAM-1, CCL-5, CCL-19, CXCL-13, GM-CSF, VCAM-1, CCL-21, and CXCL-12 in tumor tissues were quantified using ELISA. Subsequently, immunofluorescence staining and flow cytometry were used to investigate the HEV formation. For immunofluorescence staining, the frozen sections of tumor tissues were stained with the Alexa Fluor 488 anti-mouse CD31 (PECAM-1) Antibody (1:500, 4°C, overnight), Biotin Anti-mouse/human PNAd Antibody (1:100, 4°C, overnight) and PE Streptavidin (1:500, RT, 60 min), and Rabbit Anti-TNFSF14 (LIGHT) antibody (1:500, 4°C, overnight) and Goat Anti-Rabbit IgG H&L (Alexa Fluor 647) (1:1000, RT, 60 min). For flow cytometry, tumor tissues were dissociated into single-cell suspensions by passing through a 40-μm cell strainer and then were stained with the Alexa Fluor 488 anti-mouse CD31 (PECAM-1) Antibody (1:100, 4°C, 60 min), Biotin Anti-mouse/human PNAd Antibody (1:50, 4°C, overnight) and PE Streptavidin (1:100, RT, 60 min).

##### Collagen degradation

The mRNA levels of various matrix metalloproteinases (MMPs) were measured using qPCR. The contents of TGF-β, IL-10, MMP-1, MMP-9, MMP-25, hydroxyproline, and type I collagen were quantified by ELISA. The paraffin sections of tumor tissues were dewaxed and then stained with PS-Red solution (RT, 10 min). Bright-field and polarized light microscopy were employed to observe collagen density at the boundary and inside of tumor tissues, respectively. To detect the penetration depth of T cells, the frozen sections were stained with PE anti-mouse CD3 Antibody (1:500, 4°C, overnight) and FITC anti-mouse CD8a Antibody (1:500, 4°C, overnight). The thickness of collagen at the boundary of tumor tissues, percentage of collagen within tumor tissues and CD3^+^ relative gray value were processed and analyzed using ImageJ software.

##### Immunocyte infiltration

Various immunocytes were counted using flow cytometry. Tumor tissues were dissociated into single-cell suspensions. Immune cells were labeled with FITC anti-mouse CD45 Antibody (1:100, 4°C, 60 min); T cells were labeled with FITC anti-mouse CD45 Antibody (1:100, 4°C, 60 min) and APC anti-mouse CD3 Antibody (1:100, 4°C, 60 min); B cells were labeled with FITC anti-mouse CD45 Antibody (1:100, 4°C, 60 min) and APC anti-mouse/human CD45R/B220 Antibody (1:100, 4°C, 60 min); macrophages were labeled with FITC anti-mouse CD45 Antibody (1:100, 4°C, 60 min) and APC anti-mouse F4/80 Antibody (1:100, 4°C, 60 min); NK cells were labeled with FITC anti-mouse CD45 Antibody (1:100, 4°C, 60 min) and APC anti-mouse CD49b (pan-NK cells) Antibody (1:100, 4°C, 60 min); DCs were labeled with FITC anti-mouse CD45 Antibody (1:100, 4°C, 60 min) and PE anti-mouse CD11c Antibody (1:100, 4°C, 60 min). Treg cells were treated with a True-Nuclear Transcription Factor Buffer Set and then labeled with APC anti-mouse CD45 Antibody (1:100, 4°C, 60 min), FITC anti-mouse CD4 Antibody (1:100, 4°C, 60 min) and PE anti-mouse/rat/human FOXP3 Antibody (1:100, 4°C, 60 min).

##### Tertiary lymphoid structure

The frozen sections of tumor tissues were stained with FITC anti-mouse/human CD45R/B220 Antibody (1:500, 4°C, overnight), APC anti-mouse CD3 Antibody (1:500, 4°C, overnight), and Rabbit Anti-CXCL13 Recombinant Multiclonal Antibody. The numbers of B cell clusters, T cells around each B cell cluster, TLSs and deep TLSs (Depth ≥500 μm), along with area of each TLS, were counted using ImageJ software. Next, the stem-like CD8^+^ T cells and proliferating CD8^+^ T cells were analyzed by flow cytometry. Single-cell suspensions were fixed and permeabilized using a Transcription Factor Buffer Set. Then, the cells were stained with FITC anti-mouse CD8a Antibody (1:100, 4°C, 60 min), Rabbit Anti-TCF1/TCF7 Monoclonal Antibody (1:100, 4°C, overnight) and Goat Anti-Rabbit IgG H&L (Alexa Fluor 594) (1:1000, RT, 60 min) or FITC anti-mouse CD8a Antibody (1:100, 4°C, 60 min) and APC anti-mouse Ki-67 Antibody (1:100, 4°C, 60 min).

#### Function analysis of αCD3

##### Microfluidics

The microchannel, measuring 0.5 mm in height, 1 mm in width, and 20 mm in length, was fabricated with a polydimethylsiloxane (PDMS) layer on the top and sealed to a glass coverslip on the bottom. The left and right holes were connected to the injection and waste conduits (inner diameter of 0.4 mm), respectively. Firstly, the inner bottom surface of the microchannel was coated with fibronectin (50 μg/mL, 37°C, 24 h). Meanwhile, B16-OVA cells were transfected with either PBS or P_αCD3&LIGHT_ for 4 h. After 24 h, the transfected B16-OVA cells (2 × 10^5^ cells/mL) were detached using trypsin, labeled with CFDA-SE (5 μM, 37°C, 15 min) and then injected into the microchannel. After 24 h, the OT-1 cells (2 × 10^5^ cells/mL) were stained with CMTMR (5 μM, 37°C, 30 min) and then injected into the microchannel via a peristaltic pump (Pump 11 Elite, Harvard Apparatus). After allowing the OT-1 cells to settle for 60 min, PBS was injected into the channel at a flow rate of 5 μL/min for 30 min. During PBS infusion, a confocal laser scanning microscopy was used to characterize the movement of OT-1 cells in real time. Finally, the captured XYZ-stacking images were processed using Imaris 9.0 software. The percentage of OT-1 cells in proximity to B16-OVA cells was calculated based on the criterion that the shortest distance to the surface was less than or equal to 2. The track length, track straightness, and speed of OT-1 cells were analyzed by Imaris 9.0 software.

##### Enzymatic labeling

Firstly, the SrtA protein was expressed and purified. The SrtA gene sequence (deleted amino acids 1–59) from *Staphylococcus aureus* ATCC 10832 was cloned into the pET-28a vector and then transformed into *E. coli* BL21 (DE3) competent cells. The cells were cultured in LB medium (220 rpm, 37°C, 4–5 h), followed by induction with IPTG (500 μM, 220 rpm, 16°C, 16–18 h). The protein in the supernatant were purified using a Ni-NTA Spin Purification Kit and quantified by a BCA assay kit.

Next, SrtA was conjugated into OT-1 surface to construct donor cells. 10 μM SrtA was incubated with NHS-(PEG)_4_-Mal in phosphate buffer solution (pH = 8.5, 37°C, 60 min). The mixtures were desalted using a desalting column packed with G-25 resin and concentrated using a 10 kDa ultrafiltration centrifugal tube (SrtA-Mal). 1 × 10^7^ OT-1 cells were resuspended in 400 μL PBS containing the 3 mM EDTA and 0.1 mg/mL traut’s reagent (pH = 8.0, RT, 60 min) to increase free thiol group (-SH) on their surface (OT-1@Traut). Then, 1 × 10^7^ OT-1@Traut cells were mixed with 300 μg SrtA-Mal (pH = 6.5, 37°C, 2 h), designated as OT-1@SrtA. To verify whether SrtA coupled to the OT-1 surface retains its catalytic activity, OT-1@SrtA cells were stained with the Alexa Fluor 488 anti-His Tag Antibody (1:100, 4°C, 60 min) and 5-Tamra-LPETG (10 μM, 37°C, 60 min) for confocal laser scanning microscopy imaging and flow cytometry analysis.

B16-OVA cells, as the receptor cells, were specifically decorated with N-terminal glycine residues (G_5_ peptide) through glycometabolism and click chemistry. 1 × 10^7^ B16-OVA cells transfected with different plasmids were cultured in RPMI 1640 complete medium with CB-Ac_3_ManNAz (CB-Az, 200 μM, 37°C, 48 h). Following this, the cells were incubated with DBCO-G_5_ (10 μM, 37°C, 60 min), designated as B16-OVA-G_5_ cells. The different plasmid-transfected B16-OVA-G_5_ cells were stained with the FITC anti-HA.11 Epitope Tag Antibody (1:100, 4°C, 60 min), APC anti-His Tag Antibody (1:100, 4°C, 60 min) and 5-Tamra-G_5_C-DBCO (10 μM, 37°C, 60 min) for confocal laser scanning microscopy imaging and flow cytometry assay. The number of G_5_ peptide on B16-OVA-G_5_ cells was quantified using a standard curve method. A calibration curve was fitted by measuring the fluorescence values of 0, 0.2, 0.5, 1.0, 2.0, and 5.0 nM of 5-Tamra-G_5_C-DBCO. 1 × 10^7^ B16-OVA-G_5_ cells were stained with 5-Tamra-G_5_C-DBCO (1 μM, 37°C, 60 min) and then washed with PBS three times. Unstained B16-OVA-G_5_ cells served as the blank control. Fluorescence intensities were measured using a fluorescence microplate reader. The number of G_5_ was calculated according to the regression curve. In order to verify the specificity of this enzymatic labeling approach, 3 × 10^5^ Hoechst 33342-stained OT-1@SrtA cells and 1 × 10^5^ CFDA-SE-labeled B16-OVA or B16-OVA-G_5_ cells were co-cultured in a 1:1 mixture of DMEM complete medium and mouse T cell complete medium containing 5-Tamra-LPETG (10 μM, 37°C, 2 h). The transfer of 5-Tamra-LPETG was observed using a confocal microscopy and three-dimensional images were reconstructed using Imaris 9.0 software. Subsequently, 1 × 10^5^ B16-OVA-G_5_ cells transfected with different plasmids were left to incubate with 3 × 10^5^ OT-1@SrtA cells in a medium containing 5-Tamra-LPETG (10 μM, 37°C, 2 h). 5-Tamra-LPETG-positive B16-OVA-G_5_ cells were analyzed by flow cytometry.

Next, the interaction between T cells and tumor cells was detected *in vivo*. The melanoma-bearing mouse model and administration route were the same as in Chapter “Tumor-specific analysis of plasmid”. The CB-Az (50 mM) was injected at the tumor sites. After 48 h, DBCO-G_5_ (0.5 mM) was administrated *in situ*. After 24 h, 5 × 10^6^ OT-1@SrtA cells were intravenously injected into tumor-bearing mice. After 24 h, the Biotin-Ahx-LPETGS-NH_2_ (0.2 mM) was injected at the tumor sites. After 24 h, the mice were sacrificed. For flow cytometry, leukomonocytes were isolated using a tumor-infiltrating tissue lymphocyte separation solution kit and then CFDA-SE-labeled OT-1 cells were detected to evaluate the infiltration of OT-1 cells. Single-cell suspensions of tumor tissues were stained with Rabbit Anti-Melanoma gp100 Antibody (1:40, 4°C, overnight), Goat Anti-Rabbit IgG H&L (Alexa Fluor 488) (1:500, RT, 60 min) and 5-Tamra-G_5_C-DBCO (10 μM, 37°C, 60 min) to prove that tumor cells were more prone to azide derivatization compared to non-tumor cells following the subcutaneous injection of CB-Ac_3_ManNAz. Single-cell suspensions of tumor tissues were stained with Rabbit Anti-Melanoma gp100 Antibody (1:40, 4°C, overnight), Goat Anti-Rabbit IgG H&L (Alexa Fluor 488) (1:500, RT, 60 min) and PE Streptavidin (1:100, RT, 60 min) to determine the interaction between T cells and tumor cells. For immunofluorescence, the sections of tumor tissues were stained with Rabbit Anti-Melanoma gp100 Antibody (1:100, 4°C, overnight), Goat Anti-Rabbit IgG H&L (Alexa Fluor 488) (1:1000, RT, 60 min) and PE Streptavidin (1:500, RT, 60 min).

##### T cell activation and proliferation

The OT-1 cells or hCAR-T cells were co-cultured with B16-OVA or Raji cells transfected with different plasmids in T cell complete medium without the anti-CD3 antibody. OT-1 cells were collected to analyze mRNA expression levels of activation markers (CD69, CD44, and CD25) and exhaustion markers (PD-1, TIM-3, LAG-3, and CD39) by qPCR. And these cells stained with PE anti-mouse CD69 Antibody (1:100, 4°C, 60 min) or PE anti-human CD69 Antibody (1:100, 4°C, 60 min) and then determined by flow cytometry. The cell proliferation was evaluated by CCK-8 assay. Three inflammatory cytokines secreted by T cells, including IFN-γ, TNF-α, and Gzm-B, were quantified using ELISA. The cytotoxicity mediated by OT-1 or hCAR-T cells toward B16-OVA or Raji cells was evaluated using the CCK-8 assay in accordance with the manufacturer’s instructions and flow cytometric analysis of PI-stained (5 μM, RT, 15 min) apoptotic B16-OVA cells.

#### P_αCD3&LIGHT_-mediated suppression of “immune-cold” solid tumors

The melanoma-, colon carcinoma- or breast cancer-bearing mouse model was established. When the volume of tumors reached 50 mm^3^, P_αCD3&LIGHT_ and other control plasmids were intravenously injected into mice every three days for four consecutive administrations (30 μg each time). When the volume of tumors reached 1500 mm^3^, the mice were sacrificed. For flow cytometry, single-cell suspensions were stained with FITC anti-mouse CD8a Antibody (1:100, 4°C, 60 min) and PE anti-mouse CD69 Antibody (1:100, 4°C, 60 min) to analyze the activation of CD8^+^ T cells. For ELISA, the tumor tissues were thoroughly ground to obtain the homogenate and then three inflammatory cytokines in tumor tissues were quantified. Next, the length (L) and width (W) of the subcutaneous tumors were measured every three days. The tumor volumes were calculated by the formula of (L × W^2^)/2. The final tumor weights were measured. The survival time of each mouse was recorded every day. For safety evaluation, lymphocytes were isolated from peripheral blood and spleen using a Mouse Peripheral Blood Lymphocyte Isolation Kit and a Mouse Spleen Lymphocyte Isolation Kit, respectively, and then were stained with PE anti-mouse CD3 antibody for flow cytometric analysis of T cell frequency. The paraffin sections of major organs were stained with a Hematoxylin-Eosin (H&E) HD Constant Dye Kit. The body temperature and body weight of mice were monitored every three days. The serum levels of alkaline phosphatase (ALP), alanine aminotransferase (ALT), aspartate transaminase (AST), creatinine (CRE), blood urea nitrogen (BUN) and lactate dehydrogenase (LDH) were analyzed.

#### Evaluating anti-melanoma effects of P_αCD3&LIGHT_ in combination with ICIs

Next, the melanoma-bearing mouse model was the same as in the Chapter “Tumor-specific analysis of plasmid”. The anti-tumor effects were evaluated using two administration routes, including the different plasmids treatments and the combination therapy of P_αCD3&LIGHT_ and immune checkpoint inhibitors (ICIs). The plasmids were intravenously injected into mice every three days for four consecutive administrations (30 μg each time) and the ICIs, αPD-L1 and αCTLA-4, were administered intravenously on the day following the plasmid injections (30 μg each time). On the day 23, the mice were sacrificed. For flow cytometry, single-cell suspensions were stained with the FITC anti-mouse CD8a Antibody (1:100, 4°C, 60 min) and PE anti-mouse CD69 Antibody (1:100, 4°C, 60 min) to analyze the activation of CD8^+^ T cells. Single-cell suspensions were stained with Rabbit Anti-Melanoma gp100 Monoclonal Antibody (1:40, 4°C, overnight), Goat Anti-Rabbit IgG H&L (Alexa Fluor 405) (1:1000, RT, 60 min) and CellEvent Caspase-3/7 Green (500 nM, RT, 60 min) to indicate apoptosis of tumor cells. For immunofluorescence, the paraffin sections of main organs were stained with a Hematoxylin-Eosin (H&E) HD Constant Dye Kit. For ELISA, the tumor tissues were thoroughly ground to obtain the homogenate and then three inflammatory cytokines in tumor tissues were quantified. Next, the length (L) and width (W) of the subcutaneous tumors were measured every three days. The tumor volumes were calculated by the formula of (L × W^2^)/2. The tumors were weighed on the day 23. The survival time of each mouse was recorded every day. For safety evaluation, the body temperature and body weight of mice were monitored every three days. The serum levels of alkaline phosphatase (ALP), alanine aminotransferase (ALT), aspartate transaminase (AST), creatinine (CRE), blood urea nitrogen (BUN) and lactate dehydrogenase (LDH) were analyzed.

#### Assessing anti-tumor efficacy and systemic toxicity of combination therapy of P_αCD3&LIGHT_ and CAR-T

The infusion dose of CAR-T cells was optimized by balancing anti-tumor efficacy with potential side effects. The tumor model was established by subcutaneously inoculating 1 × 10^6^ hCD19-B16 cells into the right flank of 4-week-old C57BL/6 mice. Then, the mice were intravenously injected with either PBS or P_αCD3&LIGHT_ every three days for four consecutive administrations (30 μg each time). On the 12th and 18th day, mice were *i.v.* injected with hαCD19-mCAR-T cells at the total doses of 0, 0.5, 1.0, 1.5, 2.0 and 4.0 × 10^6^ cells per mouse. The mice were euthanized on the day 23. The number of CAR-T cells in TME was stained with the FITC-labeled Human CD19 (20–291) (10 μg/mL, 4°C, 60 min) and then was quantified by flow cytometry. The relative ratio of CAR-T infiltration (%) = (number of CAR-T cells present in TME)/(total number of injected CAR-T cells) × 100%. And the next, the length (L) and width (W) of the subcutaneous tumors were measured every three days. The tumor volumes were calculated by the formula of (L × W^2^)/2. Tumor weights were measured on the day 23. For evaluating the side effects of CAR-T cells, the body temperature and weight of mice were monitored. The serum was collected via orbital blood sampling and then SAA, IL-6, and IL-1β were qualified by ELISA. The paraffin sections of lung, spleen and liver tissues were stained with Rabbit Anti-mouse CD11b Polyclonal Antibody (1:500, 4°C, overnight) and HRP-conjugated Goat Anti-Rabbit IgG (1:200, RT, 60 min). The paraffin sections of brain tissues were stained using Hematoxylin-Eosin (H&E) HD Constant Dye Kit.

To evaluate the therapeutic efficacy of P_αCD3&LIGHT_ in combination with 1.5 × 10^6^ CAR-T cells, the hCD19-B16 melanoma-bearing mouse model was established. A total of 1.5 × 10^6^ DiR-labeled (5 μM, 37°C, 20 min) CAR-T cells were injected into the mice via tail vein on the 12th and 18th day. After 24 h, the IVIS spectrum imaging system was utilized to visualize the infiltration of CAR-T cells. The percentages of CD69^+^, TCF1^+^ and Ki67^+^ in CAR-T and the cleaved-caspase-3-positive hCD19-B16 cells were analyzed by flow cytometry. Three inflammatory factors in tumors were determined by ELISA. The tumor volumes were calculated by the formula of (L × W^2^)/2. The survival time of each mouse was recorded every day. Four cured mice were tested for the presence of tiny tumors through MRI imaging (3.0T MAGNETOM Trio TimSystem, Siemens).

#### Clinical relevance study of LIGHT and αCD3

The surgically excised specimens from colorectal cancer, nasopharyngeal carcinoma, and cervical cancer patients were collected. For flow cytometry, single-cell suspensions were stained with FITC anti-human CD3 Antibody (1:100, 4°C, 60 min) and PE anti-human CD8a Antibody (1:100, 4°C, 60 min) to analyze the count of human CD8^+^ T cells in tumors. For ELISA, the tumor tissues were thoroughly ground to obtain the homogenate and then the level of human LIGHT in tumors was quantified. For immunofluorescence, the paraffin sections of human cervical cancer tissues were stained with FITC anti-human CD8a Antibody (1:500, 4°C, overnight), PE anti-human CD258 (LIGHT) Antibody (1:500, 4°C, overnight). On the other side, human hαCD19-hCAR-T cells (hCAR-T) were cultured in medium supplemented with gradient concentrations of αCD3 (0.001–2 μg/mL). After 24 h, these cells were stained with PE anti-human CD69 Antibody (1:100, 4°C, 60 min) and then analyzed by flow cytometry. After 72 h, the cells were counted using an automated cell counter and the human Gzm-B level was quantified by ELISA.

### Quantification and statistical analysis

Data were reported as mean ± s.d. Statistical analysis was performed with GraphPad Prism 10 (GraphPad Software, San Diego, CA, USA). The one-way ANOVA with tukey test. (more than 2 groups) or unpaired two-tailed Student’s t test (two groups) was used to analyze the statistically significant differences and data were considered statistically significant when the values of *p* < 0.05. Statistical analyses, n.s., not significant; ∗*p* < 0.05; ∗∗*p* < 0.01; ∗∗∗*p* < 0.001; ∗∗∗∗*p* < 0.0001.

## Supplemental information

Document S1. Figures S1–S13 and Tables S1 and S2

Document S2. Article plus supplemental information

## Footnotes

Supplemental information can be found online at https://doi.org/10.1016/j.xcrm.2025.102510.
