# Potentiating immunotherapy in “immune-cold” solid tumors through orchestrating T cell immunity via tumor-specific genetic engineering

**DOI:** 10.1016/j.xcrm.2026.103110

**Published:** 2026-09-26

**Authors:** Jiaqi He, Chunguang Zhang, Chao Liang, Wenchi Xue, Yongheng Li, Lili Dai, Chunyuan Liu, Wan-Ru Zhuang, Xianbin Ma, Ran Cheng, Yao Lei, Weidong Nie, Hai-Yan Xie

## Main text

(Cell Reports Medicine *6*, 102510; December 16, 2025)

In the originally published version of this article, due to an oversight during figure preparation, the CLSM image for the MLF group in Figure 1L and the H&E-stained lung section from the Blank group in Figure S9L were inadvertently presented using duplicates of the CLSM image from the PTEC group and the H&E-stained lung section from the PαCD3&LIGHT group, respectively. The data, statistical analyses, and conclusions of the study remain unaffected. The authors sincerely regret this error, which has been corrected online.

**Figure 1L:**
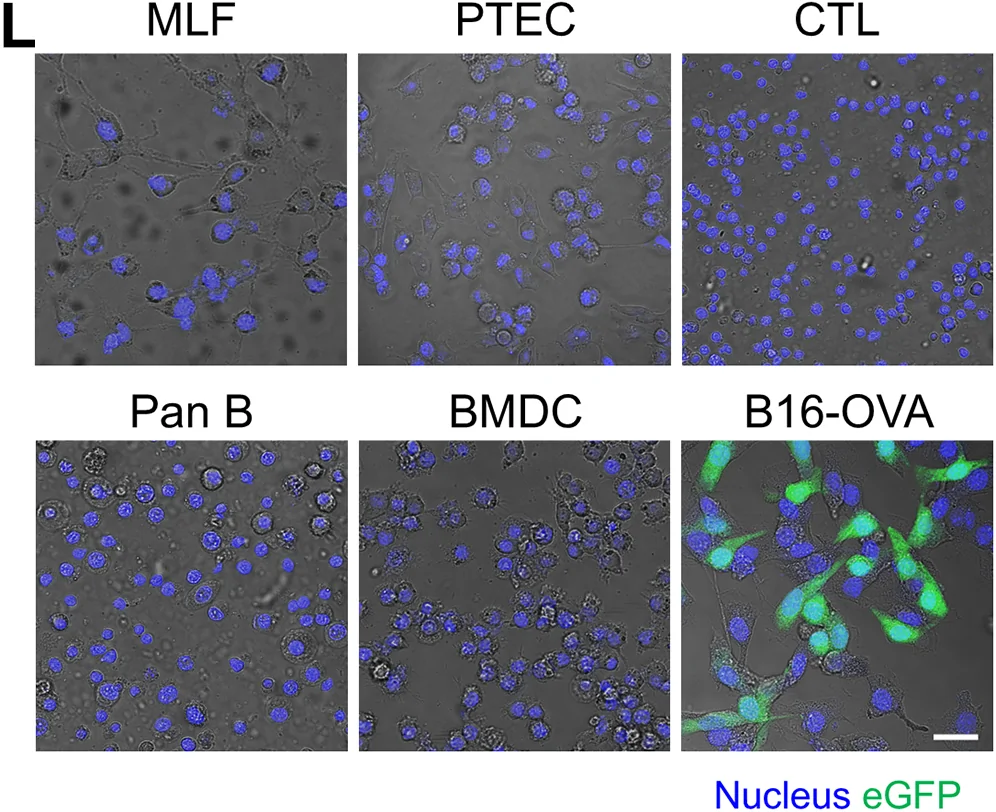
(corrected)

**Figure 1L:**
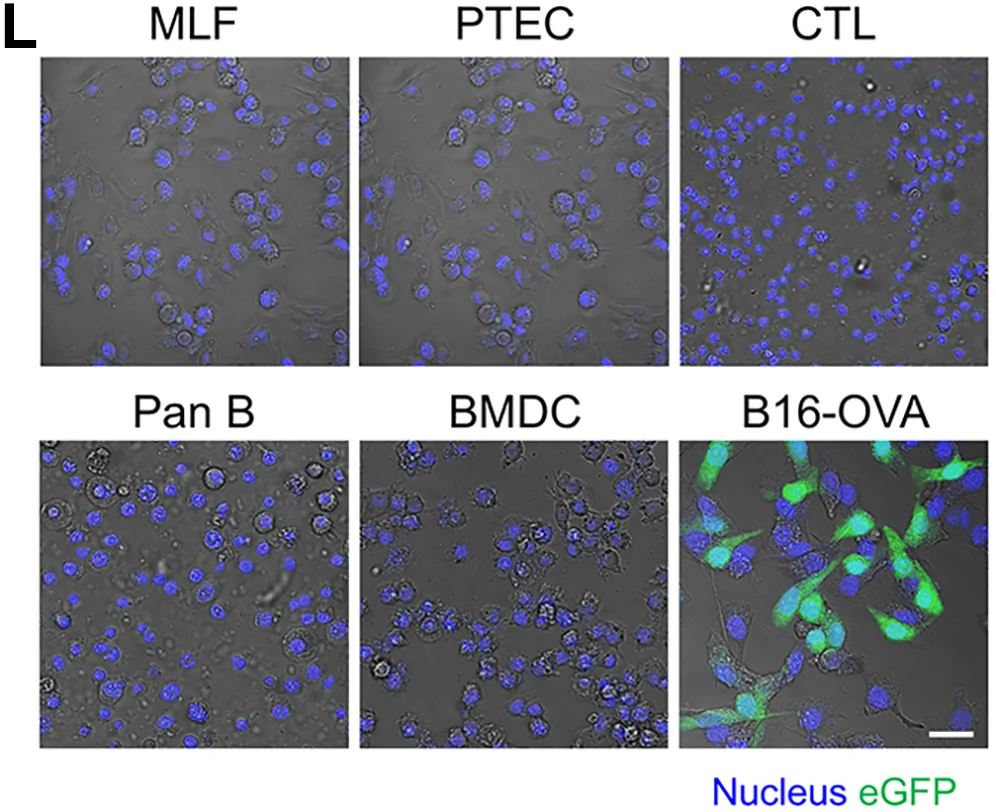
(original)

**Figure S9L:**
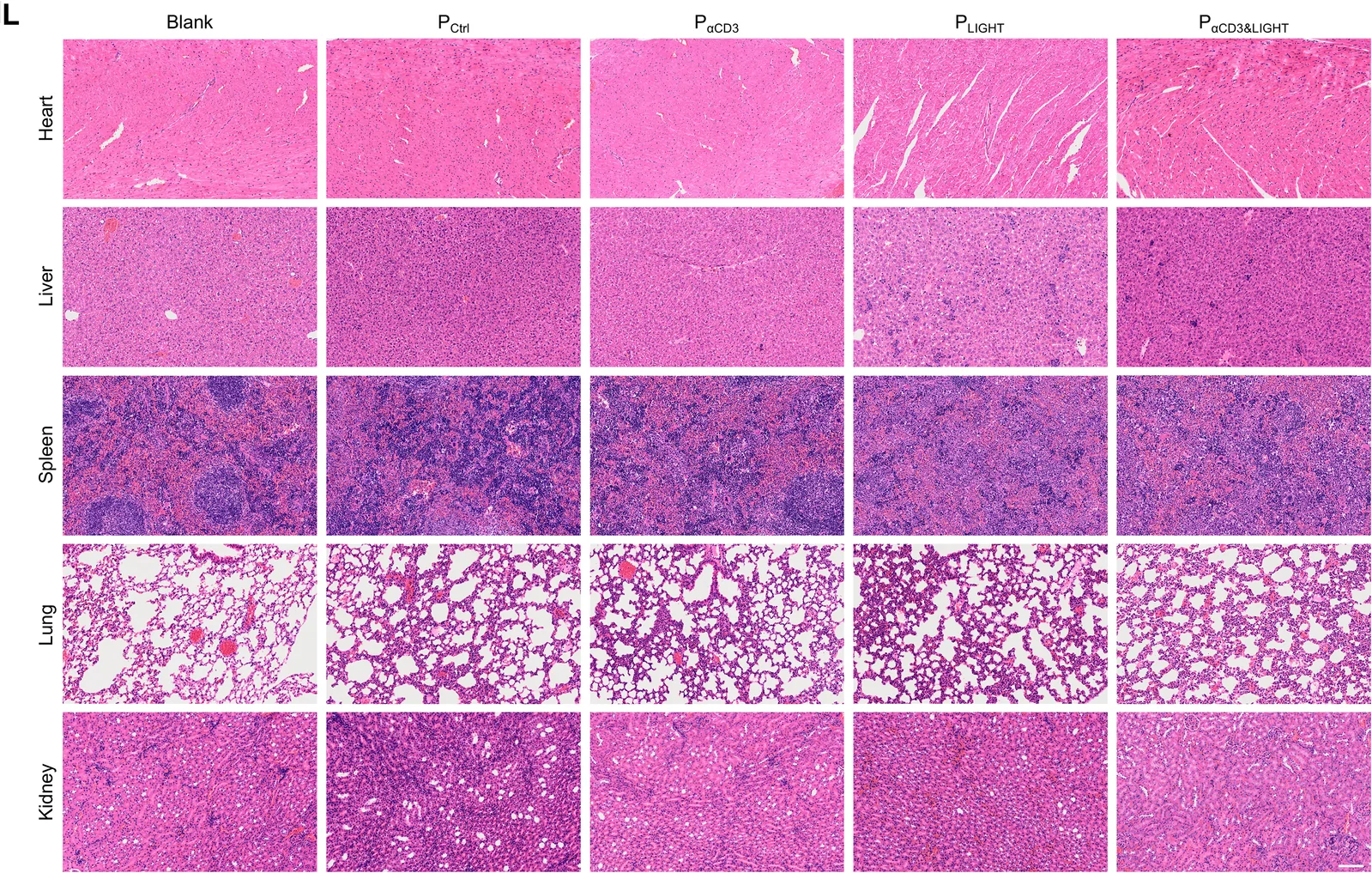
(corrected)

**Figure S9L:**
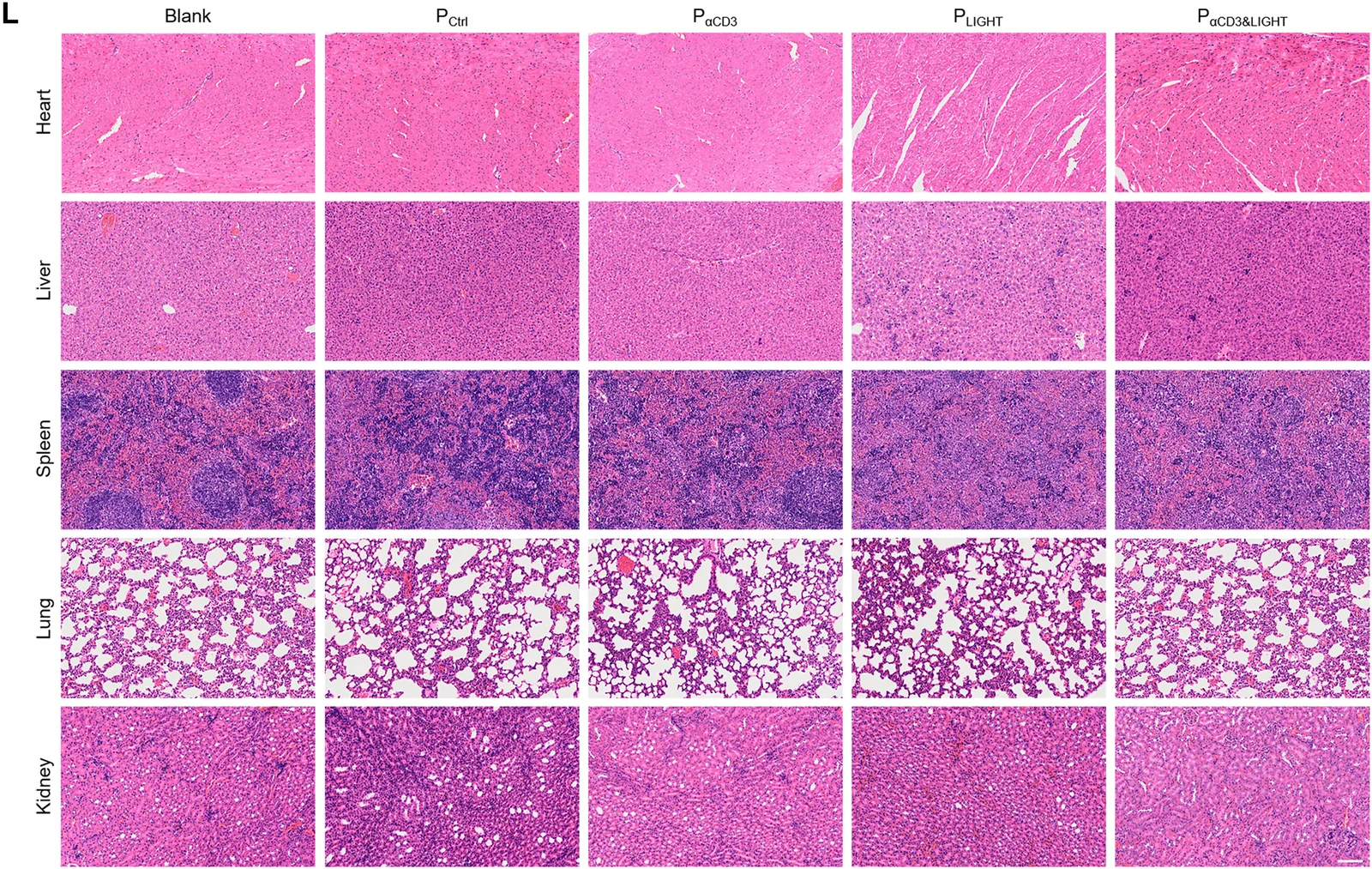
(original)

