# Supplementary material for: Potentiating immunotherapy in “immune-cold” solid tumors through orchestrating T cell immunity via tumor-specific genetic engineering
Source: Cell Rep Med. 2025 Dec 16;6(12):102510. doi: 10.1016/j.xcrm.2025.102510 (PMC12765953; doi:10.1016/j.xcrm.2025.102510)
Supplement: Document S1. Figures S1–S13 and Tables S1 and S2 [file mmc1.pdf]

Cell Reports Medicine, Volume *6*

Supplemental information

Potentiating immunotherapy in "immune-cold" solid tumors through orchestrating T cell immunity via tumor-specific genetic engineering

Jiaqi He, Chunguang Zhang, Chao Liang, Wenchi Xue, Yongheng Li, Lili Dai, Chunyuan Liu, Wan-Ru Zhuang, Xianbin Ma, Ran Cheng, Yao Lei, Weidong Nie, and Hai-Yan Xie

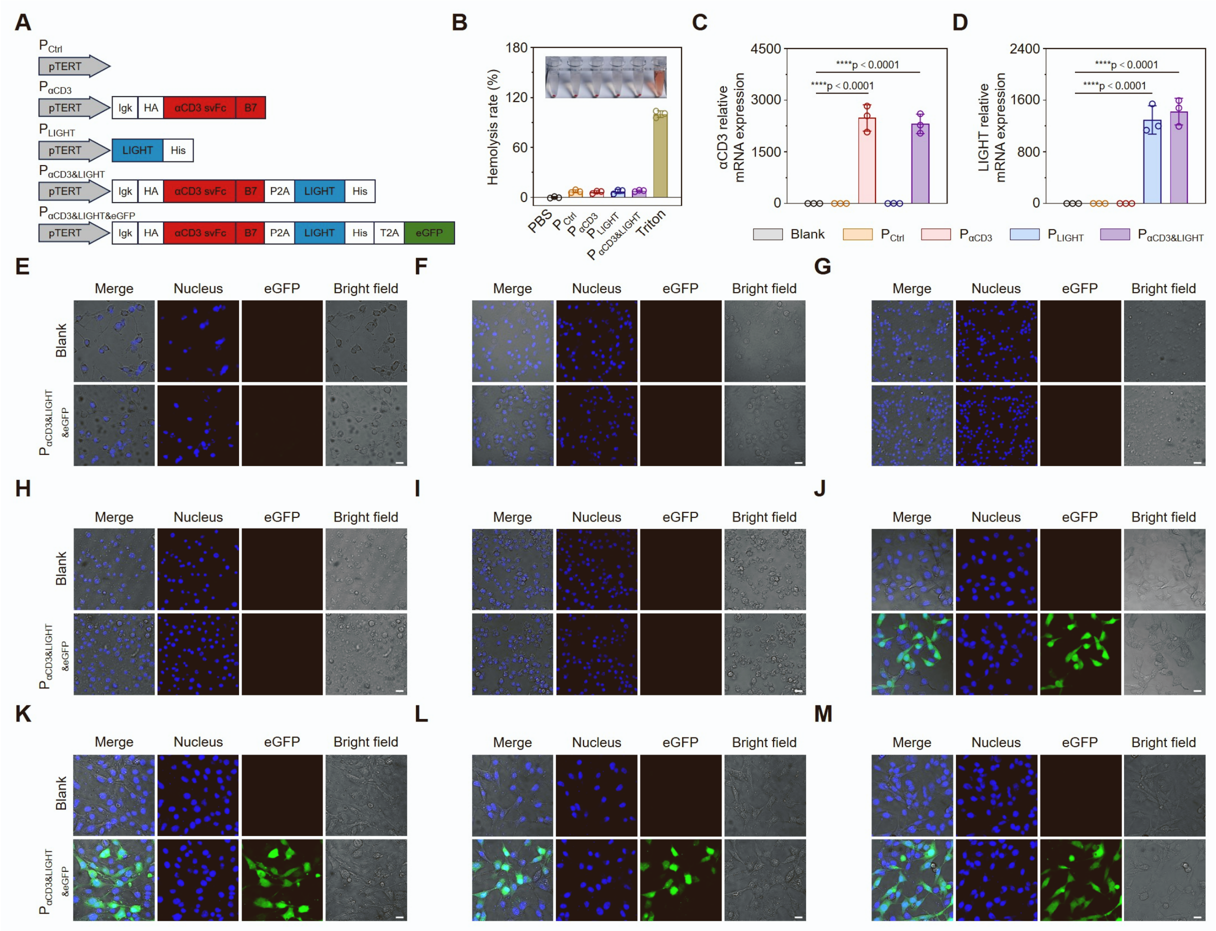

Figure S1. Tumor-specificity evaluation of $P_{\alpha CD3\&LIGHT}$ *in vitro*

(A) Schematic illustration of the structures of $P_{\alpha CD3\&LIGHT}$, $P_{\alpha CD3\&LIGHT\&eGFP}$, and other control plasmids.

(B) The hemolysis of red blood cells (RBCs) after incubation with $P_{\alpha CD3\&LIGHT}$ or other controls. *n=3*.

(C and D) Relative mRNA expression levels of *αCD3* (C) and *LIGHT* (D) in B16-OVA cells transfected with $P_{\alpha CD3\&LIGHT}$ or other controls. *n = 3*.

(E-M) Representative CLSM images of eGFP expression in lung fibroblasts (MLFs) (E), proximal tubular epithelial cells (PTECs) (F), cytotoxic T lymphocytes (CTLs) (G), Pan B cells (H), bone marrow-derived dendritic cells (BMDCs) (I), B16-OVA cells (J), 4T1 cells (K), CT26 cells (L), and MC38 cells (M) transfected with $P_{\alpha CD3\&LIGHT\&eGFP}$ or PBS. Scale bars: 15 μm.

Data are represented as mean ± SD (error bars) from biological replicates. p values were determined by one-way ANOVA with tukey test for (C) and (D). ****$p < 0.0001$. Related to Figure 1.

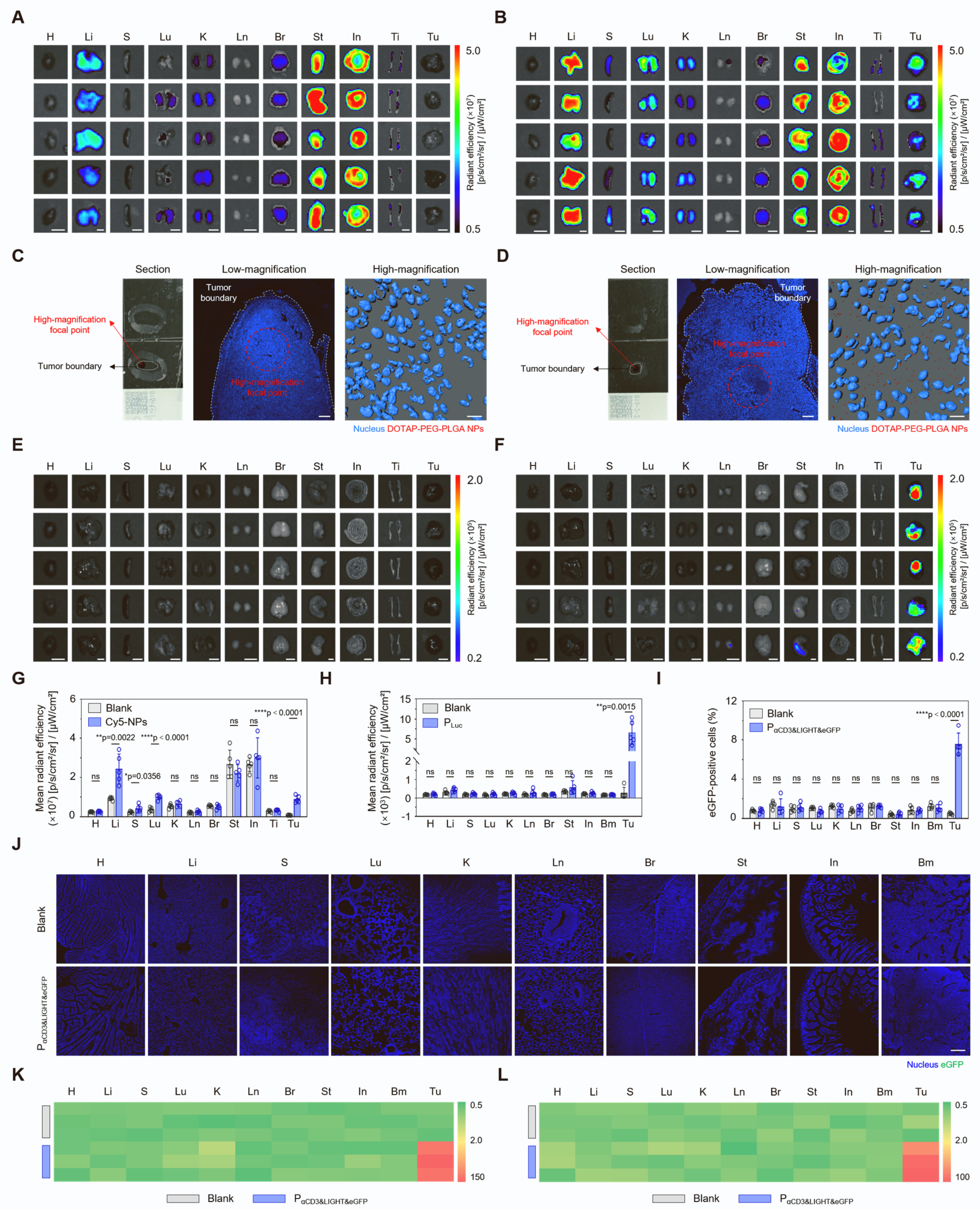

Figure S2. Tumor-specificity evaluation of $P_{αCD3\&LIGHT}$ *in vivo*

(A and B) IVIS spectrum images of Cy5 signal in tumors tissues (Tu), heart (H), liver (Li), spleen (S), lung (Lu), kidney (K), lymph node (Ln), brain (Br), stomach (St), intestine (In), and tibia (Ti) of melanoma-bearing mice after intravenous injection of PBS (A) or Cy5-labeled DOTAP-PEG-PLGA nanoparticles (B). Scale bars: 5 mm.

(C and D) Representative CLMS and three-dimensional reconstitution images of DOTAP-PEG-PLGA nanoparticles in tumors of melanoma-bearing mice after intravenous injection of PBS (C) or Cy5-labeled DOTAP-PEG-PLGA nanoparticles (D). Nucleus (Blue) and DOTAP-PEG-PLGA NPs (Red). Scale bars: 300 μm (Original images) or 15 μm (Enlarged images).

(E and F) Bioluminescence images of tumors tissues (Tu), heart (H), liver (Li), spleen (S), lung (Lu), kidney (K), lymph node (Ln), brain (Br), stomach (St), intestine (In), and tibia (Ti) of melanoma-bearing mice after intravenous injection of PBS (E) or P_{Luc} (F). Scale bars: 5 mm.

(G) Quantitative analysis the mean radiant efficiency of Cy5 signal in tumors tissues (Tu), heart (H), liver (Li), spleen (S), lung (Lu), kidney (K), lymph node (Ln), brain (Br), stomach (St), intestine (In), and tibia (Ti) of melanoma-bearing mice after intravenous injection of PBS or Cy5-labeled DOTAP-PEG-PLGA nanoparticles. *n=5*.

(H) Quantitative analysis the mean radiant efficiency of bioluminescence in tumors tissues (Tu), heart (H), liver (Li), spleen (S), lung (Lu), kidney (K), lymph node (Ln), brain (Br), stomach (St), intestine (In), and tibia (Ti) of melanoma-bearing mice after intravenous injection of PBS or P_{Luc}. *n=5*.

(I) Flow cytometry analysis of eGFP-positive cells in tumor tissues (Tu), heart (H), liver (Li), spleen (S), lung (Lu), kidney (K), lymph node (Ln), brain (Br), stomach (St), intestine (In), and bone marrow (Bm) of melanoma-bearing mice after intravenous injection of $P_{αCD3\&LIGHT\&eGFP}$ or PBS. *n = 5*.

(J) Representative immunofluorescence images of eGFP-positive cells in heart (H), liver (Li), spleen (S), lung (Lu), kidney (K), lymph node (Ln), brain (Br), stomach (St), intestine (In), and bone marrow (Bm) of melanoma-bearing mice after intravenous injection of $P_{αCD3\&LIGHT\&eGFP}$ or PBS. Scale bar: 150 μm.

(K and L) Heat maps of mRNA levels of *αCD3* (K) and *LIGHT* (L) in tumor tissues (Tu), heart (H), liver (Li), spleen (S), lung (Lu), kidney (K), lymph node (Ln), brain (Br), stomach (St), intestine (In), and bone marrow (Bm) of melanoma-bearing mice after intravenous injection of $P_{αCD3\&LIGHT\&eGFP}$ or PBS. *n = 3*.

Data are represented as mean ± SD (error bars) from biological replicates. p values were determined by unpaired two-tailed Student t-test for (G-I). n.s., not significant; *p < 0.05; **p < 0.01; ****p < 0.0001. Related to Figure 1.

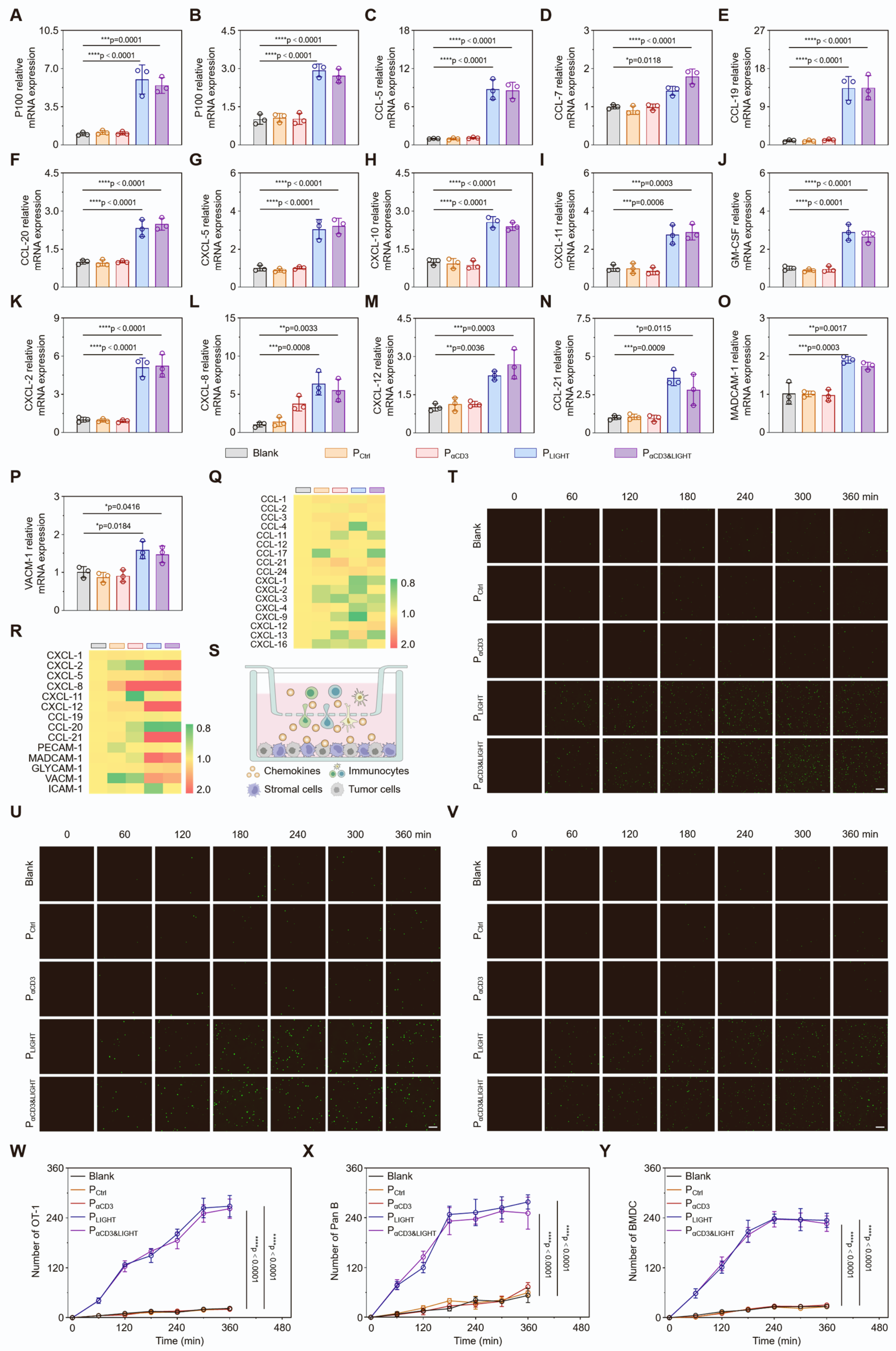
A
B
C
D
E
F
G
H
I
J
K
L
M
N
O
P
Q
R
S
T
U
V
W
X
Y
P100 relative mRNA expression
CCL-5 relative mRNA expression
CCL-7 relative mRNA expression
CCL-19 relative mRNA expression
CCL-20 relative mRNA expression
CXCL-5 relative mRNA expression
CXCL-10 relative mRNA expression
CXCL-11 relative mRNA expression
GM-CSF relative mRNA expression
CXCL-2 relative mRNA expression
CXCL-8 relative mRNA expression
CXCL-12 relative mRNA expression
CCL-21 relative mRNA expression
MADCAM-1 relative mRNA expression
VACM-1 relative mRNA expression
Blank
PCtrl
PαCD3
PLIGHT
PαCD3&LIGHT
Chemokines
Immunocytes
Stromal cells
Tumor cells
0 60 120 180 240 300 360 min
Number of OT-1
Number of Pan B
Number of BMDC
Time (min)
****p < 0.0001

Figure S3. $P_{αCD3\&LIGHT}$ induced migration of immunocytes *in vitro*

(A and B) Relative mRNA expression level of *p100* in cancer-associated fibroblasts (CAFs) (A) or C166 cells (B) following co-incubation with B16-OVA cells transfected with $P_{αCD3\&LIGHT}$ or other controls. *n = 3*.

(C-J) Relative mRNA expression levels of *CCL-5* (C), *CCL-7* (D), *CCL-19* (E), *CCL-20* (F), *CXCL-5* (G), *CXCL-10* (H), *CXCL-11* (I), and *GM-CSF* (J) in cancer-associated fibroblasts (CAFs) following co-incubation with B16-OVA cells transfected with $P_{αCD3\&LIGHT}$ or other controls. *n = 3*.

(K-P) Relative mRNA expression levels of *CXCL-2* (K), *CXCL-8* (L), *CXCL-12* (M), *CCL-21* (N), *MADCAM-1* (O), and *VACM-1* (P) in C166 cells following co-incubation with B16-OVA cells transfected with $P_{αCD3\&LIGHT}$ or other controls. *n = 3*.

(Q and R) Heat maps of the mRNA levels of chemokines and adhesion molecules secreted by cancer-associated fibroblasts (CAFs) (Q) and C166 cells (R) following co-incubation with B16-OVA cells transfected with $P_{αCD3\&LIGHT}$ or other controls.

(S) Sematic illustration of $P_{αCD3\&LIGHT}$-mediated recruitment of immunocytes.

(T-V) Representative CLSM images of CFDA-SE-labeled OT-1 cells (T), Pan B cells (U), and bone marrow-derived dendritic cells (BMDCs) (V) recruited into the lower chamber of the transwell plates in the $P_{αCD3\&LIGHT}$ or other control groups. Scale bar: 100 μm.

(W-Y) Quantitative analysis of the numbers of CFDA-SE-labeled OT-1 cells (W), Pan B cells (X), and BMDCs (Y). *n = 3*.

Data are represented as mean ± SD (error bars) from biological replicates. p values were determined by one-way ANOVA with tukey test for (A-N) and (U-W). *p < 0.05; **p < 0.01; ***p < 0.001; ****p < 0.0001. Related to Figure 2.

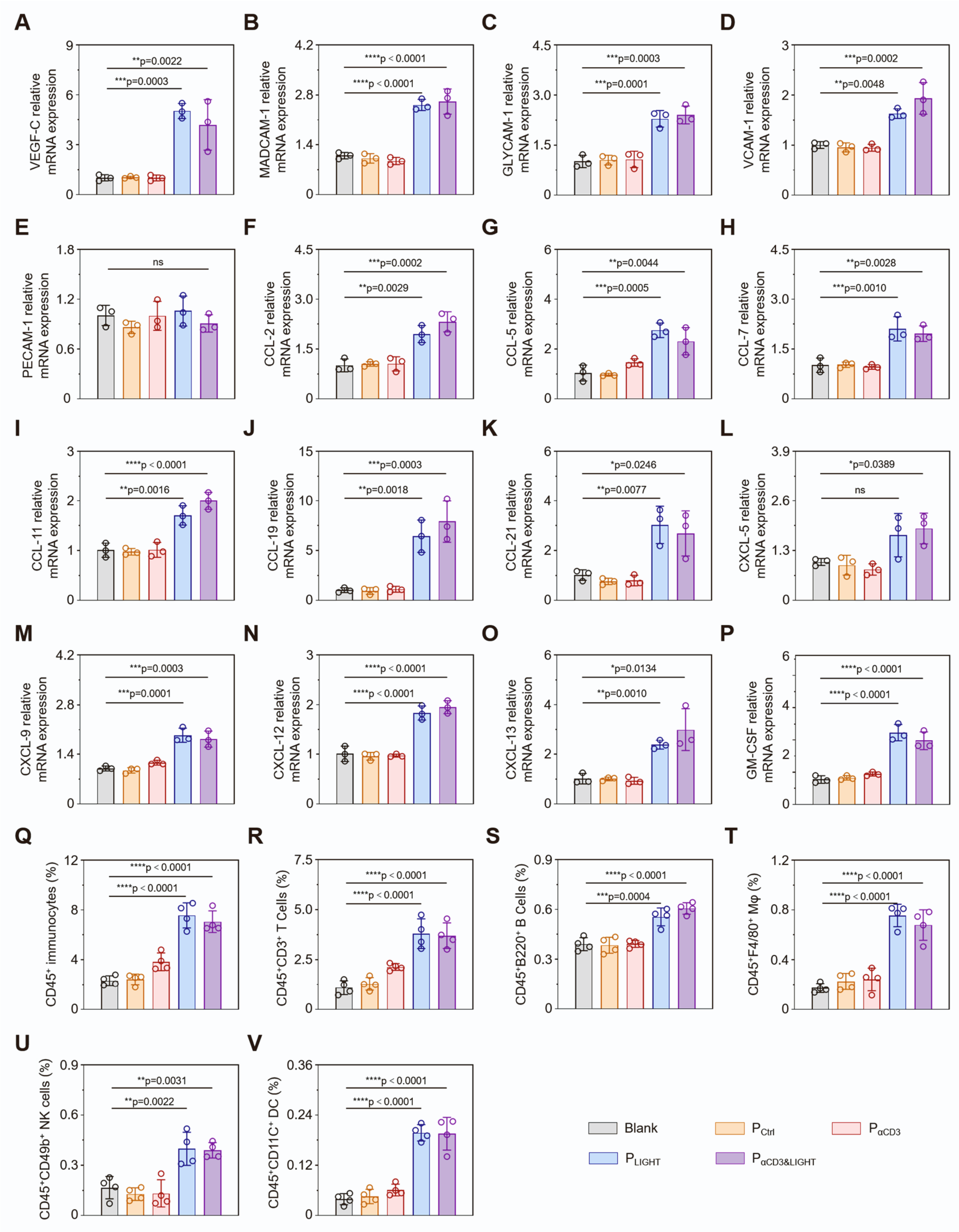

Figure S4. The directional migration of immune cells into solid tumors

(A-P) Relative mRNA expression levels of *VEGF-C* (A), *MADCAM-1* (B), *GLYCAM-1* (C), *VCAM-1* (D), *PECAM-1* (E), *CCL-2* (F), *CCL-5* (G), *CCL-7* (H), *CCL-11* (I), *CCL-19* (J), *CCL-21* (K), *CXCL-5* (L), *CXCL-9* (M), *CXCL-12* (N), *CXCL-13* (O), and *GM-CSF* (P) in tumor tissues of melanoma-bearing mice treated with $P_{αCD3\&LIGHT}$ or other controls. *n = 3*.

(Q-V) Flow cytometry analysis of $CD45^+$ immunocytes (Q), $CD45^+CD3^+$ T cells (R), $CD45^+B220^+$ B cells (S), $CD45^+F4/80^+$ macrophages (T), $CD45^+CD49b^+$ NK cells (U), and $CD45^+CD11C^+$ DCs (V) infiltrating into the tumor tissues of melanoma-bearing mice treated with $P_{αCD3\&LIGHT}$ or other controls. *n = 4*.

Data are represented as mean ± SD (error bars) from biological replicates. p values were determined by one-way ANOVA with tukey test for (A-V). n.s., not significant; *p < 0.05; **p < 0.01; ***p < 0.001; ****p < 0.0001. Related to Figure 2.

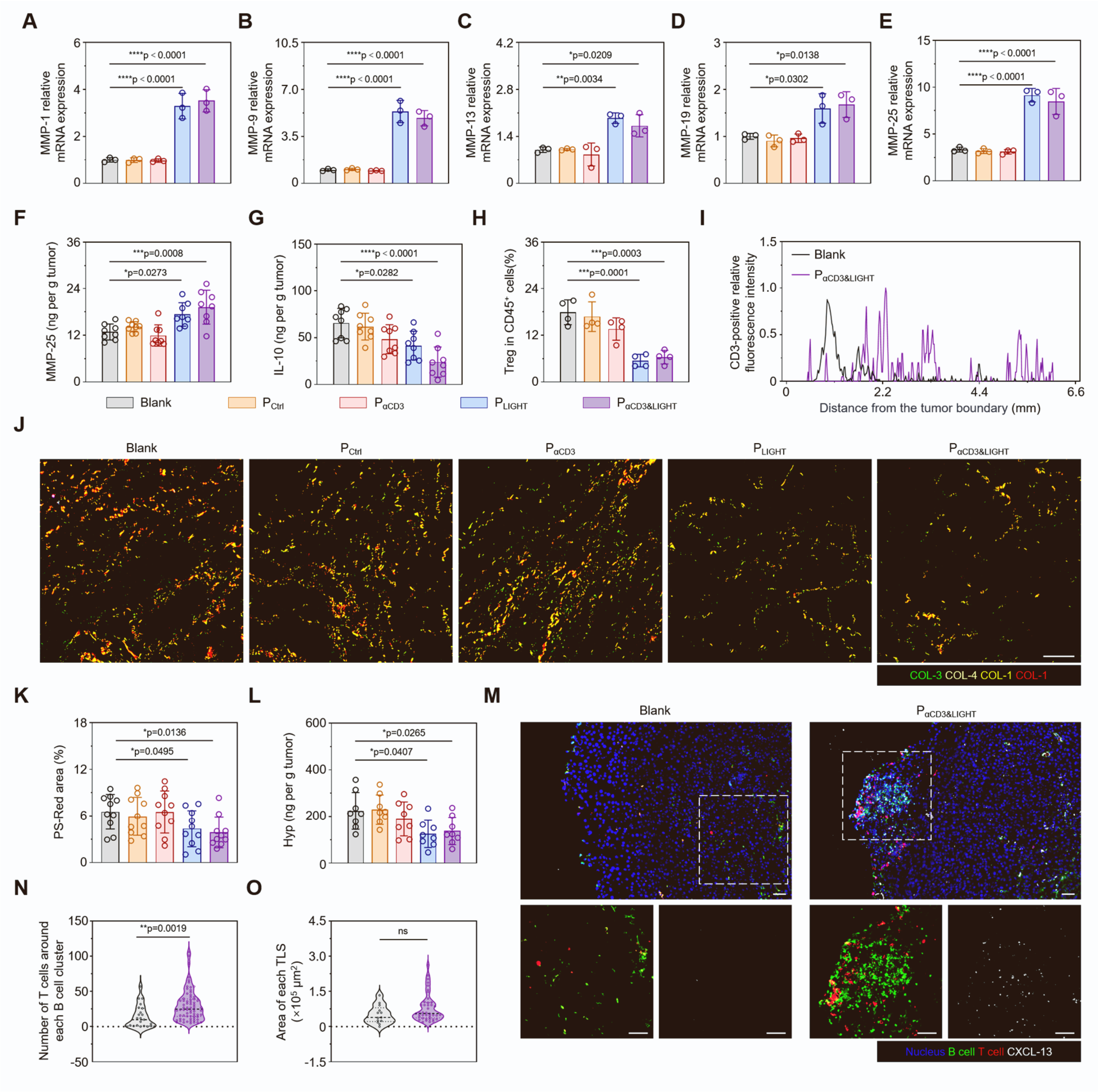

Figure S5. The collagen degradation and TLS formation

(A-E) Relative mRNA expression levels of *MMP-1* (A), *MMP-9* (B), *MMP-13* (C), *MMP-19* (D), and *MMP-25* (E) in tumor tissues of melanoma-bearing mice treated with $P_{\alpha CD3\&LIGHT}$ or other controls. *n = 3*.

(F and G) ELISA of MMP-25 (F) and IL-10 (G) in tumor tissues of melanoma-bearing mice treated with $P_{\alpha CD3\&LIGHT}$ or other controls. *n = 8*.

(H) Flow cytometry analysis of Treg cells in tumor tissues of melanoma-bearing mice treated with $P_{\alpha CD3\&LIGHT}$ or other control. *n = 4*.

(I) Relative fluorescence intensity of CD3-positive signal at varying distances from the tumor boundary in the PBS and $P_{\alpha CD3\&LIGHT}$ groups.

(J) Representative polarization microscopy images of Picro-Sirius Red (PS-Red)-positive collagen fiber inside the tumors in the $P_{\alpha CD3\&LIGHT}$ and other control groups. COL-3 (Green), COL-4 (Light yellow) and COL-1 (Yellow and red). Scale bar: 100 μm.

(K) Quantitative analysis of the ratio of the PS-Red-positive collagen area. *n = 10*.

(L) ELISA of hydroxyproline (Hyp) (L) in tumor tissues of melanoma-bearing mice treated with $P_{αCD3\&LIGHT}$ or other controls. *n = 8*.

(M) Representative immunofluorescence images of TLSs in tumor tissues of the melanoma-bearing mice after treatment with $P_{αCD3\&LIGHT}$ or PBS. Nucleus (Blue), $B220^{+}$ B cells (Green), $CD3^{+}$ T cells (Red) and CXCL-13 (White). Scale bars: 50 μm.

(N) The number of CD3-positive T cells around each B cell cluster in the PBS and $P_{αCD3\&LIGHT}$ groups. *n = 23 or 69*.

(O) The area of each TLS in the PBS and $P_{αCD3\&LIGHT}$ groups. *n = 13 or 41*.

Data are represented as mean ± SD (error bars) from biological replicates. p values were determined by one-way ANOVA with tukey test for (A-H), (K) and (L) and unpaired two-tailed Student t-test for (N) and (O). n.s., not significant; *$p < 0.05$; **$p < 0.01$; ***$p < 0.001$; ****$p < 0.0001$. Related to Figure 3.

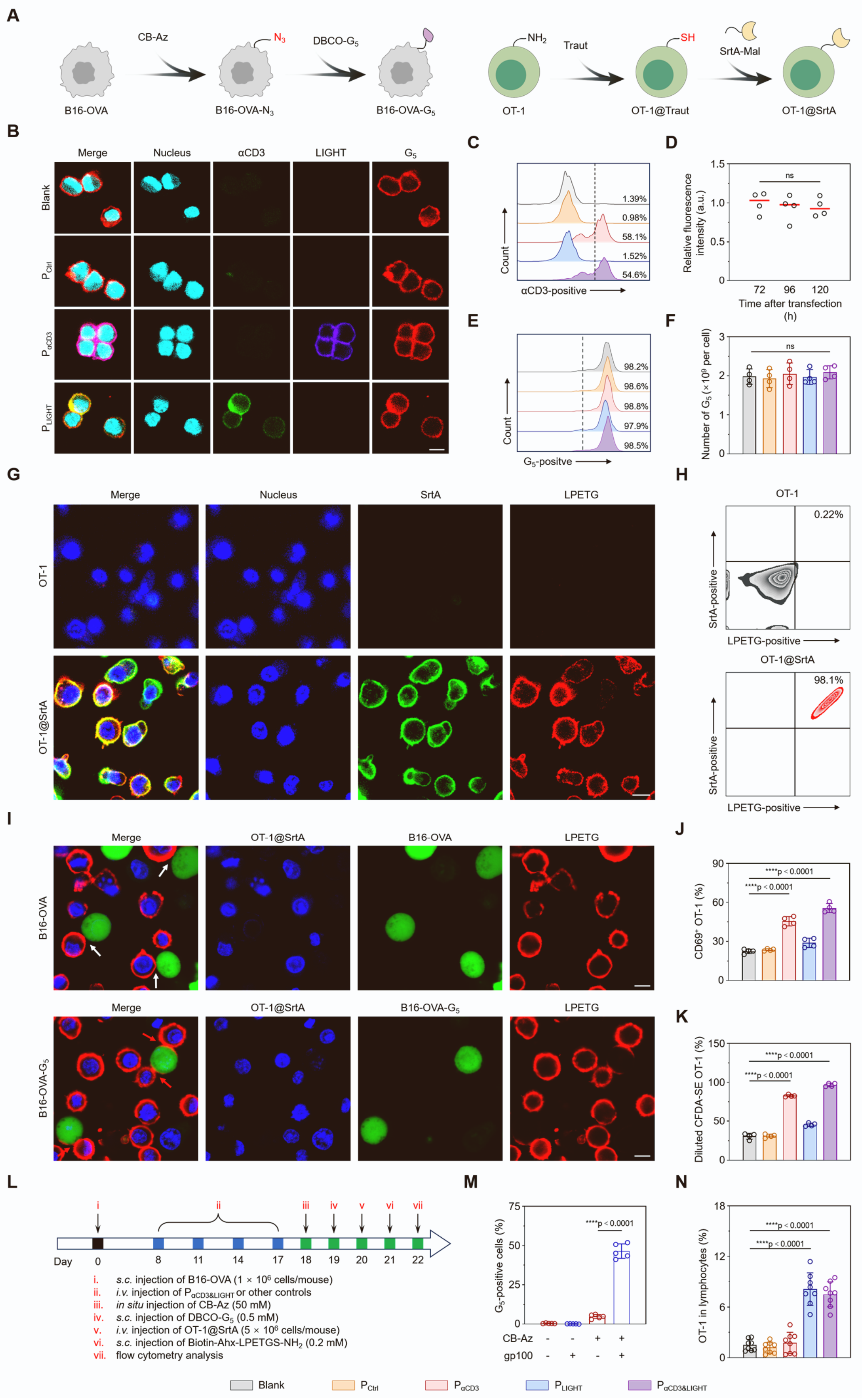
A
CB-Az
DBCO-G5
B16-OVA
B16-OVA-N3
B16-OVA-G5
NH2
Traut
SH
SrtA-Mal
OT-1
OT-1@Traut
OT-1@SrtA
B
Merge
Nucleus
αCD3
LIGHT
G5
Blank
PCtrl
PαCD3
PLIGHT
C
Count
αCD3-positive
1.39%
0.98%
58.1%
1.52%
54.6%
D
Relative fluorescence intensity (a.u.)
ns
Time after transfection (h)
E
Count
G5-positve
98.2%
98.6%
98.8%
97.9%
98.5%
F
Number of G5 (× 10⁶ per cell)
ns
G
Merge
Nucleus
SrtA
LPETG
OT-1
OT-1@SrtA
H
OT-1
0.22%
SrtA-positive
LPETG-positive
OT-1@SrtA
98.1%
I
Merge
OT-1@SrtA
B16-OVA
LPETG
B16-OVA-G5
J
CD69⁺ OT-1 (%)
****p < 0.0001
K
Diluted CFDA-SE OT-1 (%)
****p < 0.0001
L
Day
i. s.c. injection of B16-OVA (1 × 10⁶ cells/mouse)
ii. i.v. injection of PαCD3&LIGHT or other controls
iii. in situ injection of CB-Az (50 mM)
iv. s.c. injection of DBCO-G5 (0.5 mM)
v. i.v. injection of OT-1@SrtA (5 × 10⁶ cells/mouse)
vi. s.c. injection of Biotin-Ahx-LPETGS-NH2 (0.2 mM)
vii. flow cytometry analysis
M
G5-positive cells (%)
****p < 0.0001
CB-Az
gp100
N
OT-1 in lymphocytes (%)
****p < 0.0001
Blank
PCtrl
PαCD3
PLIGHT
PαCD3&LIGHT

Figure S6. System preparation of B16-OVA-G_5 cells and OT-1@SrtA cells, and monitoring their interaction

(A) Schematic illustration of the modifications of B16-OVA-G_5 cells and OT-1@SrtA cells.

(B) Representative CLMS images of G_5 peptide on the surface of B16-OVA cells transfected with $P_{αCD3\&LIGHT}$ or other controls. Nucleus (Cyan), αCD3 (Green), LIGHT (Purple) and G_5 peptide (Red). Scale bar: 10 μm. *n = 4*.

(C) Flow cytometric analysis of αCD3-positive B16-OVA cells transfected with $P_{αCD3\&LIGHT}$ or other controls.

(D) Relative fluorescence intensity in B16-OVA cells transfected with $P_{αCD3\&LIGHT}$ at 72-, 96-, and 120-hours post-transfection. *n = 4*.

(E and F) Flow cytometric analysis (E), and quantitative analysis of the number (F) of G_5 peptide on the surface of B16-OVA cells transfected with $P_{αCD3\&LIGHT}$ or other controls. *n = 4*.

(G and H) Representative CLMS images (G) and flow cytometric analysis (H) of SrtA loaded on the surface of OT-1 cells. Nucleus (Blue), SrtA (Green) and LPETG peptide (Red). Scale bar: 10 μm.

(I) Representative CLMS images of the interaction of OT-1@SrtA cells and B16-OVA or B16-OVA-G_5 cells. The white and red arrows indicated the contact surfaces between OT-1@SrtA cells with B16-OVA or B16-OVA-G_5 cells, respectively. Nucleus (Blue), B16-OVA and B16-OVA-G_5 cells (Green), and LPETG peptide (Red). Scale bar: 10 μm.

(J and K) Flow cytometric analysis of CD69-positive activated OT-1 cells (J) and diluted CFDA-SE-labeled OT-1 cells (K) and in the $P_{αCD3\&LIGHT}$ or other control groups. *n = 4*.

(L) Schematic illustration of detecting *in vivo* interaction of tumor cells and OT-1 cells using SrtA-mediated proximity labeling approach.

(M) Flow cytometric analysis of G_5-positive non B16-OVA cells and B16-OVA cells in tumor tissues after intratumoral injection of CB-AC_3ManNAz. *n = 5*.

(N) Flow cytometric analysis of OT-1 cells in lymphocytes in the $P_{αCD3\&LIGHT}$ or other control groups. *n = 8*.

Data are represented as mean ± SD (error bars) from biological replicates. p values were determined by one-way ANOVA with tukey test for (C-F), (J), (K), (M) and (N) and unpaired two-tailed Student t-test for (M). n.s., not significant; ****$p < 0.0001$. Related to Figures 4 and 5.

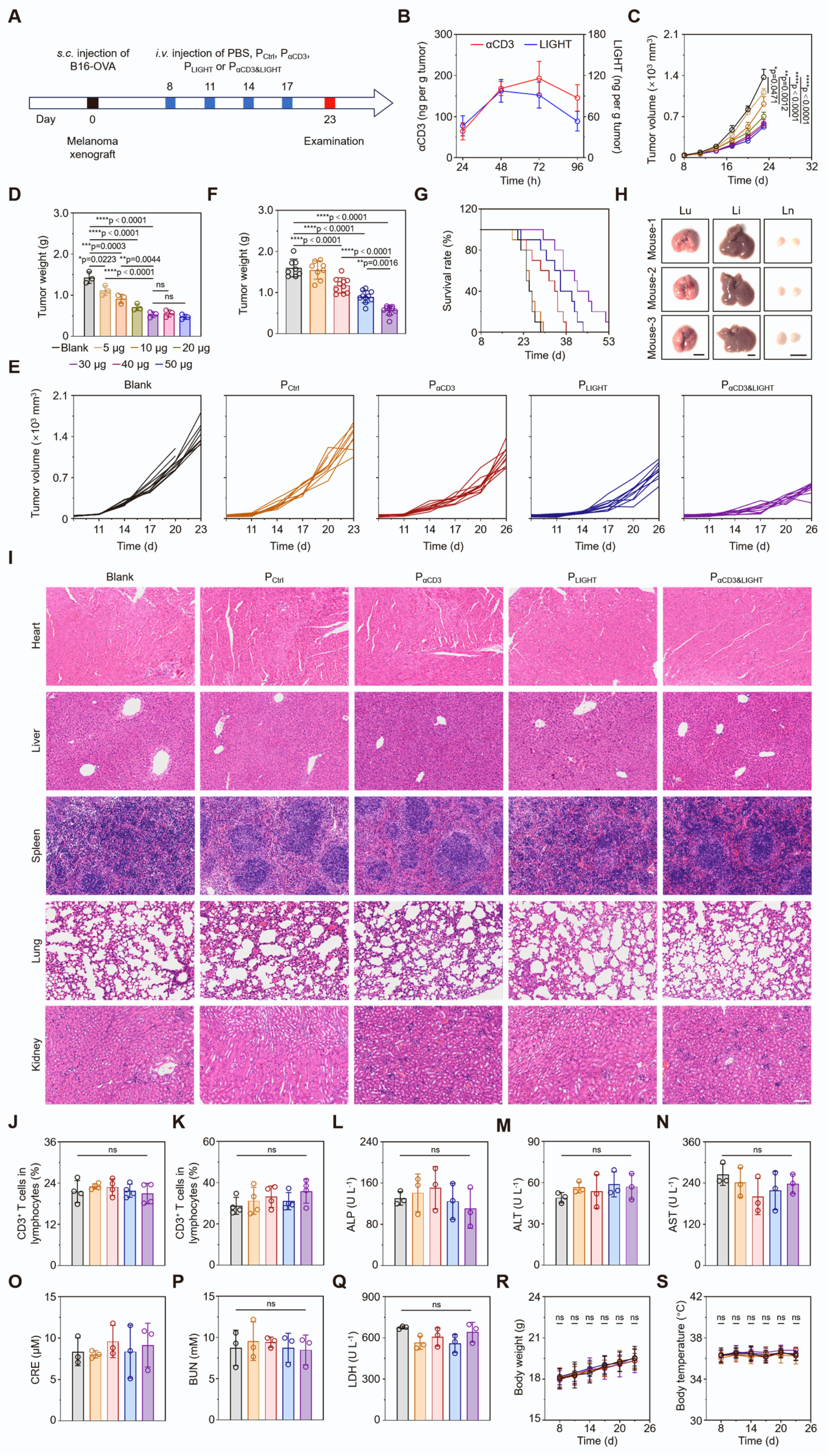

A
s.c. injection of B16-OVA
i.v. injection of PBS, PCtrl, PαCD3, PLIGHT or PαCD3&LIGHT
Day 0 8 11 14 17 23
Melanoma xenograft
Examination
B
αCD3 LIGHT
αCD3 (ng per g tumor)
LIGHT (ng per g tumor)
Time (h)
C
Tumor volume (×10³ mm³)
Time (d)
D
Tumor weight (g)
Blank 5 μg 10 μg 20 μg 30 μg 40 μg 50 μg
E
Blank PCtrl PαCD3 PLIGHT PαCD3&LIGHT
Time (d)
F
Tumor weight (g)
G
Survival rate (%)
Time (d)
H
Lu Li Ln
Mouse-1 Mouse-2 Mouse-3
I
Blank PCtrl PαCD3 PLIGHT PαCD3&LIGHT
Heart Liver Spleen Lung Kidney
J
CD3⁺ T cells in lymphocytes (%)
K
CD3⁺ T cells in lymphocytes (%)
L
ALP (U L⁻¹)
M
ALT (U L⁻¹)
N
AST (U L⁻¹)
O
CRE (μM)
P
BUN (mM)
Q
LDH (U L⁻¹)
R
Body weight (g)
Time (d)
S
Body temperature (°C)
Time (d)

Figure S7. Anti-tumor efficacy and biosafety of $P_{αCD3\&LIGHT}$ in inhibiting the B16 melanoma
(A) Therapeutic scheme of different plasmids. Melanoma-bearing mice were treated with four intravenous injections of $P_{αCD3\&LIGHT}$ or other controls.
(B) ELISA of αCD3 and LIGHT in tumor tissues of the melanoma-bearing mice treated with $P_{αCD3\&LIGHT}$. *n=3*.
(C and D) Tumor growth curves (C) and tumor weights (D) of the melanoma-bearing mice treated with $P_{αCD3\&LIGHT}$ at doses of 0-50 μg per mouse. *n=3*.
(E-G) Individual tumor growth curves (E), tumor weights (F), and survival curves (G) of the melanoma-bearing mice treated with $P_{αCD3\&LIGHT}$ or other controls. *n = 8-10*.
(H) Representative images of lung (Lu), liver (Li), and lymph node (Ln) of melanoma-bearing mice treated with $P_{αCD3\&LIGHT}$. Scale bars: 5 mm.
(I) Representative immunohistochemistry images of H&E staining of heart, liver, spleen, lung, and kidney tissues from melanoma-bearing mice treated with $P_{αCD3\&LIGHT}$ or other controls. Scale bar: 100 μm.
(J and K) Flow cytometric analysis of $CD3^+$ T cells in lymphocytes of peripheral blood (J) and spleen (K) from melanoma-bearing mice treated with $P_{αCD3\&LIGHT}$ or other controls. *n=4*.
(L-Q) The serum levels of alkaline phosphatase (ALP) (L), alanine aminotransferase (ALT) (M), aspartate aminotransferase (AST) (N), creatinine (CRE) (O), blood urea nitrogen (BUN) (P), and lactate dehydrogenase (LDH) (Q) from melanoma-bearing mice treated with $P_{αCD3\&LIGHT}$ or other controls. *n = 3*.
(R and S) The body weight (R) and body temperature (S) of melanoma-bearing mice treated with $P_{αCD3\&LIGHT}$ or other controls. *n = 8 or 10*.
Data are represented as mean ± SD (error bars) from biological replicates. p values were determined by one-way ANOVA with tukey test for (C), (D), (F) and (J-S). n.s., not significant; *p < 0.05; **p < 0.01; ***p < 0.001; ****p < 0.0001. Related to Figure 5.

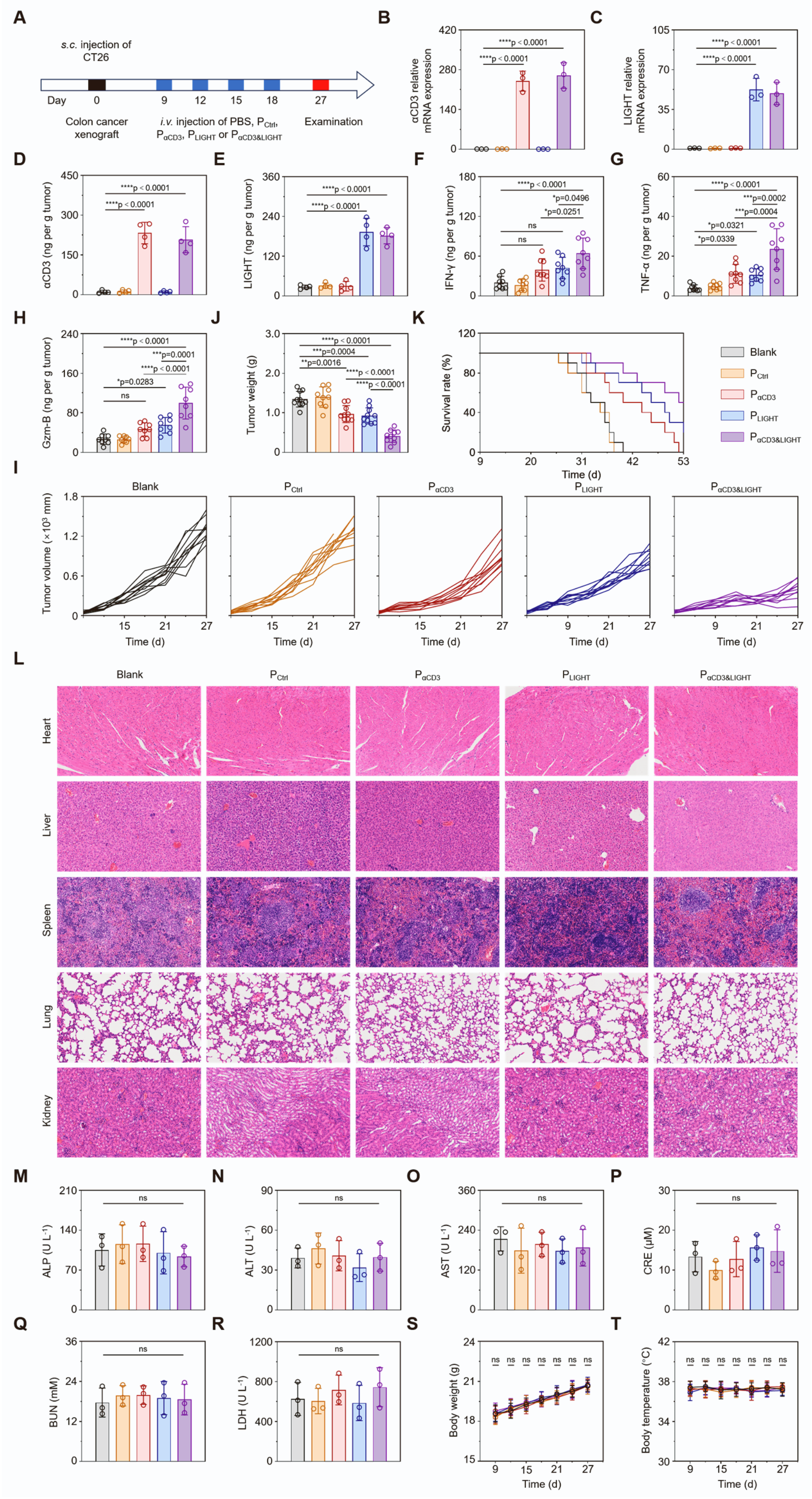
A
s.c. injection of CT26
Day 0 9 12 15 18 27
Colon cancer xenograft
i.v. injection of PBS, PCtrl, PαCD3, PLIGHT or PαCD3&LIGHT
Examination
B
αCD3 relative mRNA expression
****p < 0.0001
C
LIGHT relative mRNA expression
D
αCD3 (ng per g tumor)
E
LIGHT (ng per g tumor)
F
IFN-γ (ng per g tumor)
*p=0.0496
*p=0.0251
ns
G
TNF-α (ng per g tumor)
***p=0.0002
***p=0.0004
*p=0.0321
*p=0.0339
H
Gzm-B (ng per g tumor)
***p=0.0001
*p=0.0283
J
Tumor weight (g)
***p=0.0004
**p=0.0016
K
Survival rate (%)
Time (d)
Blank
PCtrl
PαCD3
PLIGHT
PαCD3&LIGHT
I
Tumor volume (×10³ mm)
Time (d)
L
Heart
Liver
Spleen
Lung
Kidney
M
ALP (U L⁻¹)
N
ALT (U L⁻¹)
O
AST (U L⁻¹)
P
CRE (μM)
Q
BUN (mM)
R
LDH (U L⁻¹)
S
Body weight (g)
T
Body temperature (°C)

Figure S8. Anti-tumor efficacy and biosafety of $P_{αCD3\&LIGHT}$ in inhibiting the CT26 colon carcinoma

(A) Therapeutic scheme of different plasmids. Colon carcinoma-bearing mice were treated with four intravenous injections of $P_{αCD3\&LIGHT}$ or other controls.

(B and C) Relative mRNA expression levels of *αCD3* (B) and *LIGHT* (C) in tumor tissues of colon carcinoma-bearing mice treated with $P_{αCD3\&LIGHT}$ or other controls. *n = 3*.

(D and E) ELISA of αCD3 (D) and LIGHT (E) in tumor tissues of colon carcinoma-bearing mice treated with $P_{αCD3\&LIGHT}$ or other controls. *n = 4*.

(F-H) ELISA of IFN-γ (F), TNF-α (G), and Gzm-B (H) in tumor tissues of colon carcinoma-bearing mice treated with $P_{αCD3\&LIGHT}$ or other controls. *n = 8*.

(I-K) Individual tumor growth curves (I), tumor weights (J), and survival curves (K) of the colon carcinoma-bearing mice treated with $P_{αCD3\&LIGHT}$ or other controls. *n = 9-10*.

(L) Representative immunohistochemistry images of H&E staining of heart, liver, spleen, lung, and kidney tissues from colon carcinoma-bearing mice treated with $P_{αCD3\&LIGHT}$ or other controls. Scale bar: 100 μm.

(M-R) The serum levels of alkaline phosphatase (ALP) (M), alanine aminotransferase (ALT) (N), aspartate aminotransferase (AST) (O), creatinine (CRE) (P), blood urea nitrogen (BUN) (Q), and lactate dehydrogenase (LDH) (R) from colon carcinoma-bearing mice treated with $P_{αCD3\&LIGHT}$ or other controls. *n = 3*.

(S and T) The body weight (S) and body temperature (T) of colon carcinoma-bearing mice treated with $P_{αCD3\&LIGHT}$ or other controls. *n = 9 or 10*.

Data are represented as mean ± SD (error bars) from biological replicates. p values were determined by one-way ANOVA with tukey test for (B-H), (J) and (M-T). n.s., not significant; *p < 0.05; **p < 0.01; ***p < 0.001; ****p < 0.0001. Related to Figure 5.

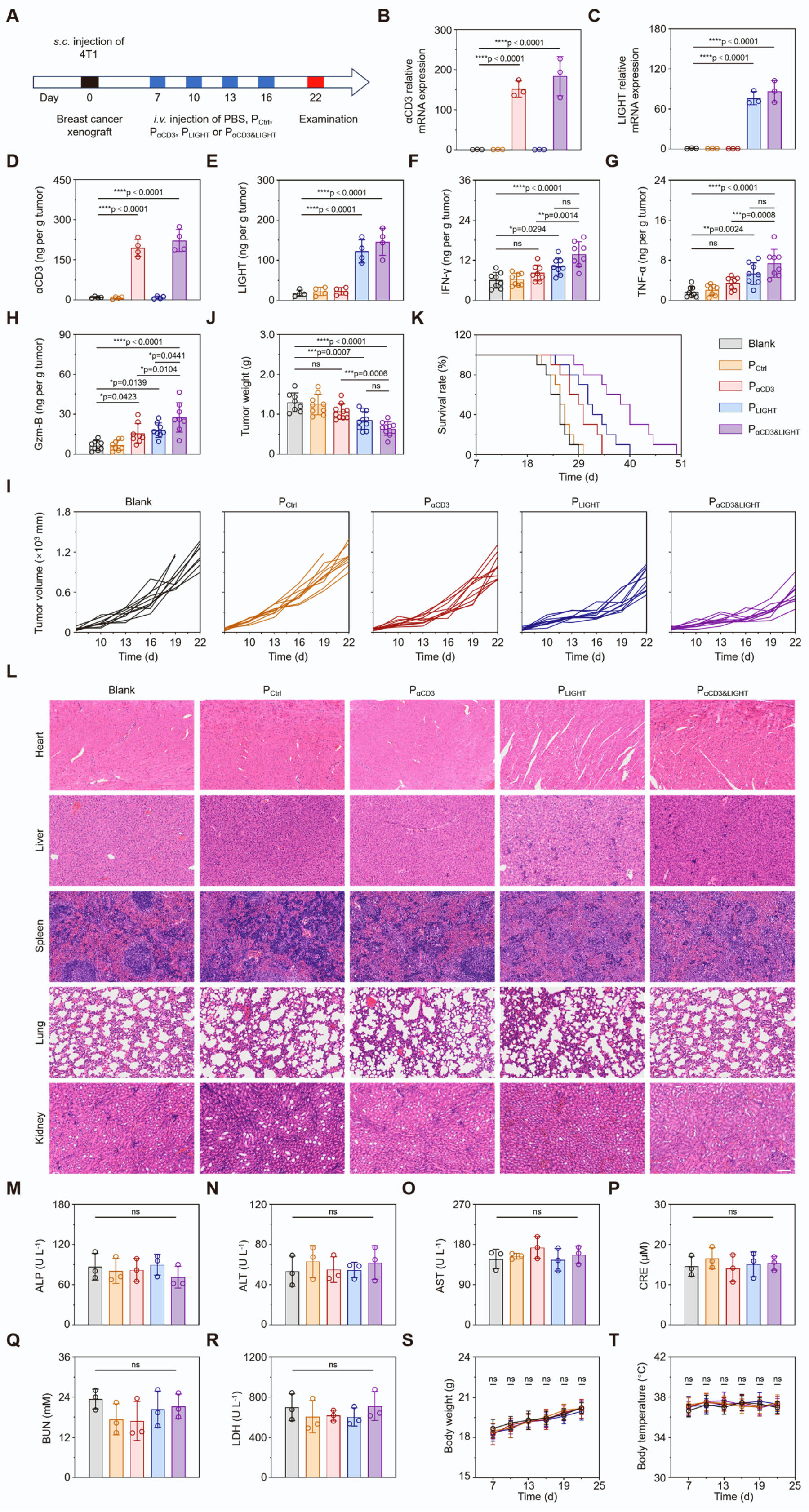

s.c. injection of 4T1
Day 0 7 10 13 16 22
Breast cancer xenograft
i.v. injection of PBS, PCtrl, PαCD3, PLIGHT or PαCD3&LIGHT
Examination
αCD3 relative mRNA expression
LIGHT relative mRNA expression
αCD3 (ng per g tumor)
LIGHT (ng per g tumor)
IFN-γ (ng per g tumor)
TNF-α (ng per g tumor)
Gzm-B (ng per g tumor)
Tumor weight (g)
Survival rate (%)
Time (d)
Blank
PCtrl
PαCD3
PLIGHT
PαCD3&LIGHT
Tumor volume (×10³ mm)
Heart
Liver
Spleen
Lung
Kidney
ALP (U L⁻¹)
ALT (U L⁻¹)
AST (U L⁻¹)
CRE (μM)
BUN (mM)
LDH (U L⁻¹)
Body weight (g)
Body temperature (°C)
ns

Figure S9. Anti-tumor efficacy and biosafety of $P_{\alpha CD3\&LIGHT}$ in inhibiting the 4T1 breast cancer

(A) Therapeutic scheme of different plasmids. Breast cancer-bearing mice were treated with four intravenous injections of $P_{\alpha CD3\&LIGHT}$ or other controls.

(B and C) Relative mRNA expression levels of *αCD3* (B) and *LIGHT* (C) in tumor tissues of breast cancer-bearing mice treated with $P_{\alpha CD3\&LIGHT}$ or other controls. *n = 3*.

(D and E) ELISA of αCD3 (D) and LIGHT (E) in tumor tissues of breast cancer-bearing mice treated with $P_{\alpha CD3\&LIGHT}$ or other controls. *n = 4*.

(F-H) ELISA of IFN-γ (F), TNF-α (G), and Gzm-B (H) in tumor tissues of breast cancer-bearing mice treated with $P_{\alpha CD3\&LIGHT}$ or other controls. *n = 8*.

(I-K) Individual tumor growth curves (I), tumor weights (J), and survival curves (K) of the breast cancer-bearing mice treated with $P_{\alpha CD3\&LIGHT}$ or other controls. *n = 8-10*.

(L) Representative immunohistochemistry images of H&E staining of heart, liver, spleen, lung, and kidney tissues from breast cancer-bearing mice treated with $P_{\alpha CD3\&LIGHT}$ or other controls. Scale bar: 100 μm.

(M-R) The serum levels of alkaline phosphatase (ALP) (M), alanine aminotransferase (ALT) (N), aspartate aminotransferase (AST) (O), creatinine (CRE) (P), blood urea nitrogen (BUN) (Q), and lactate dehydrogenase (LDH) (R) from breast cancer-bearing mice treated with $P_{\alpha CD3\&LIGHT}$ or other controls. *n = 3*.

(S and T) The body weight (S) and body temperature (T) of breast cancer-bearing mice treated with $P_{\alpha CD3\&LIGHT}$ or other controls. *n = 8-10*.

Data are represented as mean ± SD (error bars) from biological replicates. p values were determined by one-way ANOVA with tukey test for (B-H), (J) and (M-T). n.s., not significant; *$p < 0.05$; **$p < 0.01$; ***$p < 0.001$; ****$p < 0.0001$. Related to Figure 5.

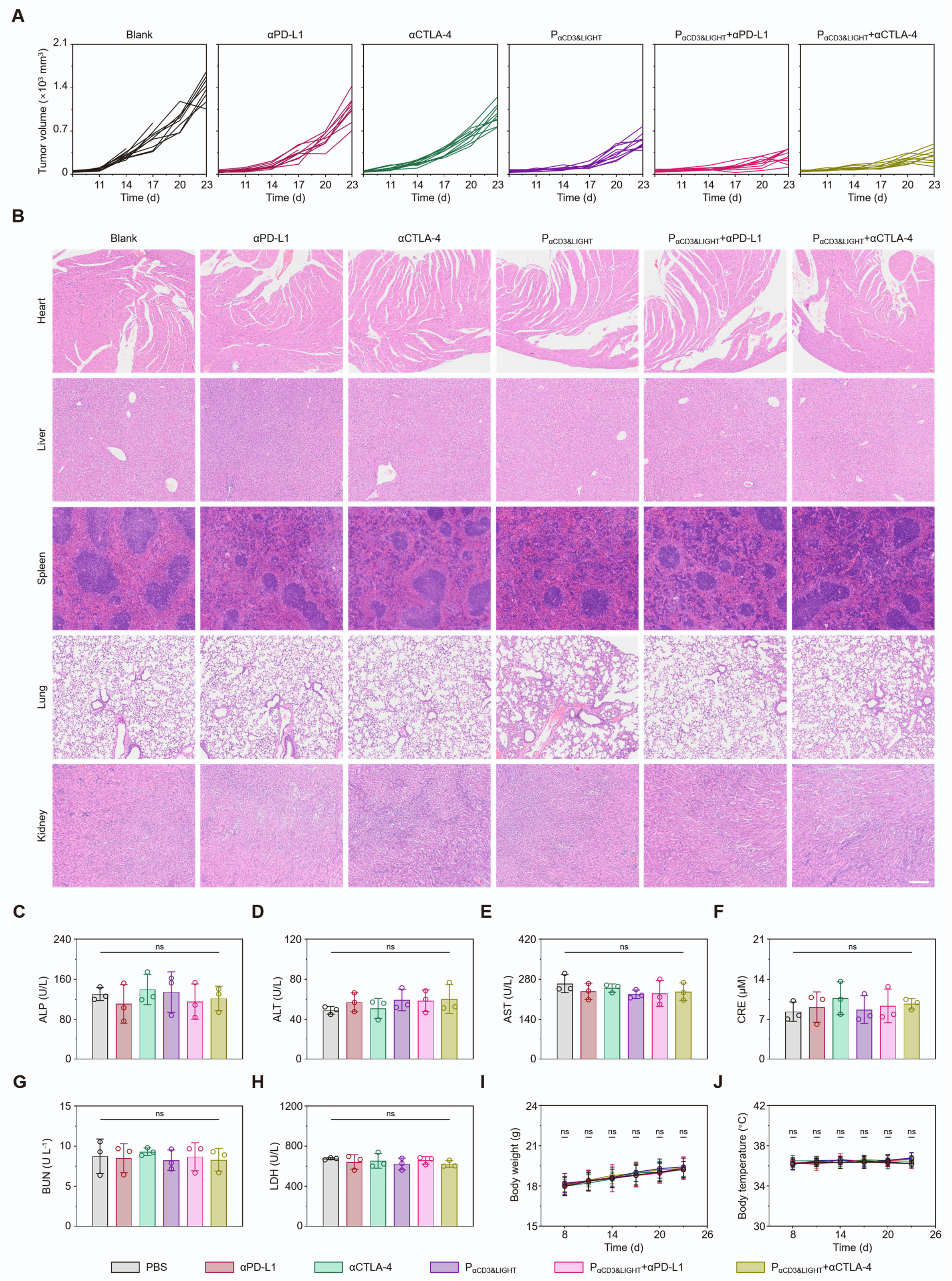
A
Blank
αPD-L1
αCTLA-4
PαCD3&LIGHT
PαCD3&LIGHT+αPD-L1
PαCD3&LIGHT+αCTLA-4
Tumor volume (×10³ mm³)
Time (d)
B
Heart
Liver
Spleen
Lung
Kidney
C
ALP (U/L)
D
ALT (U/L)
E
AST (U/L)
F
CRE (μM)
G
BUN (U L⁻¹)
H
LDH (U/L)
I
Body weight (g)
J
Body temperature (°C)
ns
PBS
αPD-L1
αCTLA-4
PαCD3&LIGHT
PαCD3&LIGHT+αPD-L1
PαCD3&LIGHT+αCTLA-4

Figure S10. The tumor suppressive effects and biosafety of $P_{αCD3\&LIGHT}$ in combination with immune checkpoint inhibitors

(A) Individual tumor growth curves of melanoma-bearing mice treated with $P_{αCD3\&LIGHT}$ + ICIs or other controls. *n = 10*.

(B) Representative immunohistochemistry images of H&E staining of heart, liver, spleen, lung, and kidney tissues from melanoma-bearing mice treated with $P_{αCD3\&LIGHT}$ + ICIs or other controls. Scale bar: 200 μm.

(C-H) The serum levels of alkaline phosphatase (ALP) (C), alanine aminotransferase (ALT) (D), aspartate aminotransferase (AST) (E), creatinine (CRE) (F), blood urea nitrogen (BUN) (G), and lactate dehydrogenase (LDH) (H) from melanoma-bearing mice treated with $P_{αCD3\&LIGHT}$ + ICIs or other controls. *n = 3*.

(I and J) The body weight (I) and body temperature (J) of melanoma-bearing mice treated with $P_{αCD3\&LIGHT}$ + ICIs or other controls. *n = 10*.

Data are represented as mean ± SD (error bars) from biological replicates. p values were determined by one-way ANOVA with tukey test for (C-J). n.s., not significant. Related to Figure 5.

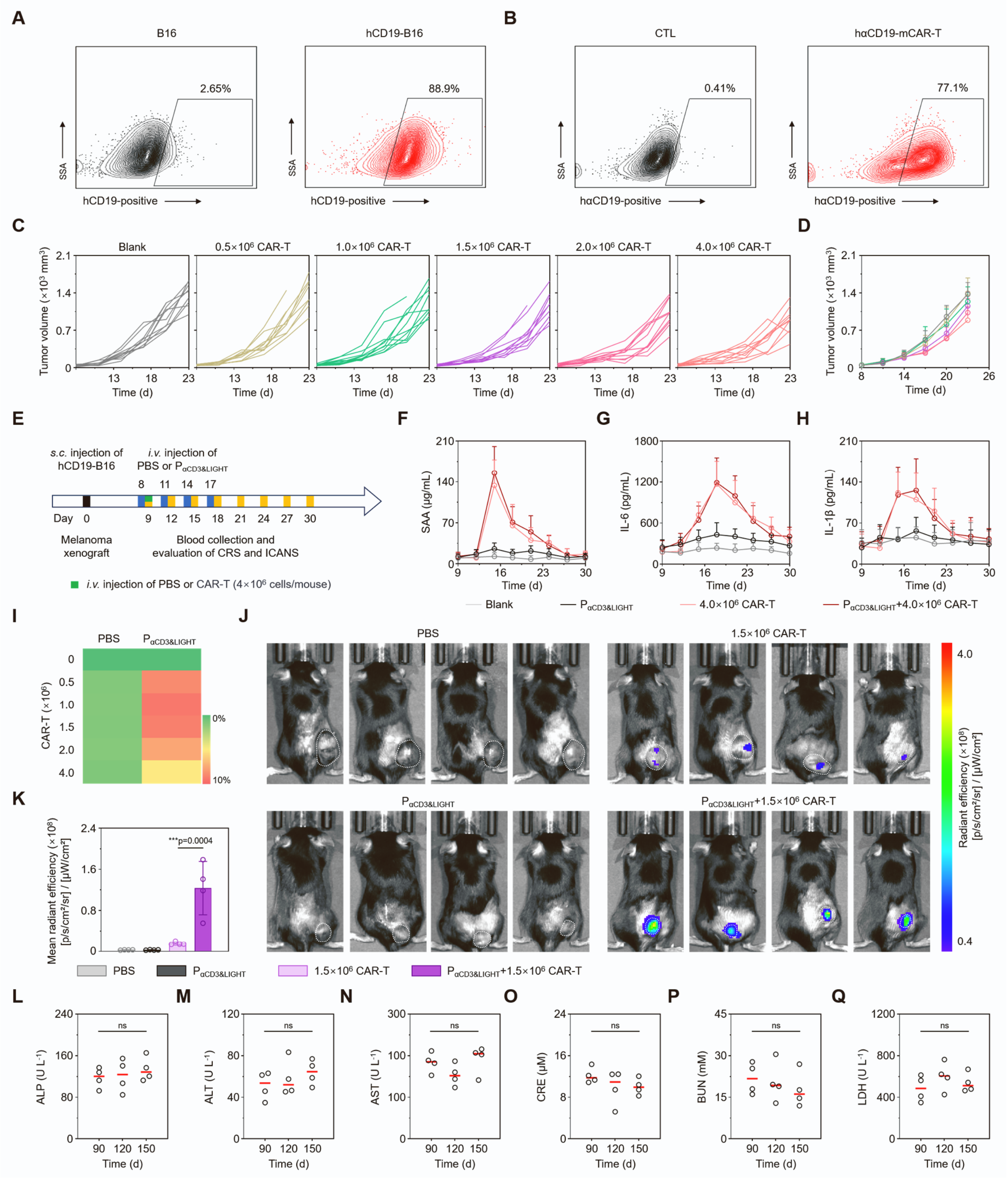

Figure S11. The performance against melanoma and side effect of $P_{αCD3\&LIGHT}$ in combination with CAR-T cells

(A) Flow cytometry plots of hCD19-positive cells in B16 cells and hCD19-B16 cells.

(B) Flow cytometry plots of hAnti-CD19-positive cells in CTLs and hαCD19-mCAR-T cells.

(C and D) Individual (C) and average (D) tumor growth curves of the hCD19-B16 melanoma-bearing mice treated with 0-4 × 10^6 CAR-T cells per mouse. *n = 9 or 10*.

(E) Schematic illustration of experimental timeline of blood serum collection for evaluating the severe cytokine release (CRS) and immune effector cell-associated neurotoxicity syndrome (ICANS).
(F-H) ELISA of SAA (F), IL-6 (G), and IL-1β (H) in blood serum from the hCD19-B16 melanoma-bearing mice treated with $P_{αCD3\&LIGHT}$ + 4.0 × 10^6 CAR-T cells or other controls. *n = 8.*
(I) The relative infiltration ratio of CAR-T cells after intravenous injection of 0-4 × 10^6 CAR-T cells.
(J) IVIS spectrum images of DiR-labeled CAR-T cells at tumor sites in hCD19-B16 melanoma-bearing mice treated with $P_{αCD3\&LIGHT}$ + 1.5 × 10^6 CAR-T cells or other controls.
(K) Quantitative analysis the mean radiant efficiency of DiR-labeled CAR-T cells at tumor sites in hCD19-B16 melanoma-bearing mice treated with $P_{αCD3\&LIGHT}$ + 1.5 × 10^6 CAR-T cells or other controls. *n = 4*.
(L-Q) The serum levels of alkaline phosphatase (ALP) (L), alanine aminotransferase (ALT) (M), aspartate aminotransferase (AST) (N), creatinine (CRE) (O), blood urea nitrogen (BUN) (P), and lactate dehydrogenase (LDH) (Q) from complete cured melanoma-bearing mice treated with $P_{αCD3\&LIGHT}$ + 1.5 × 10^6 CAR-T cells. *n = 4*.
Data are represented as mean ± SD (error bars) from biological replicates. p values were determined by one-way ANOVA with tukey test for (L-Q). n.s., not significant. Related to Figure 6.

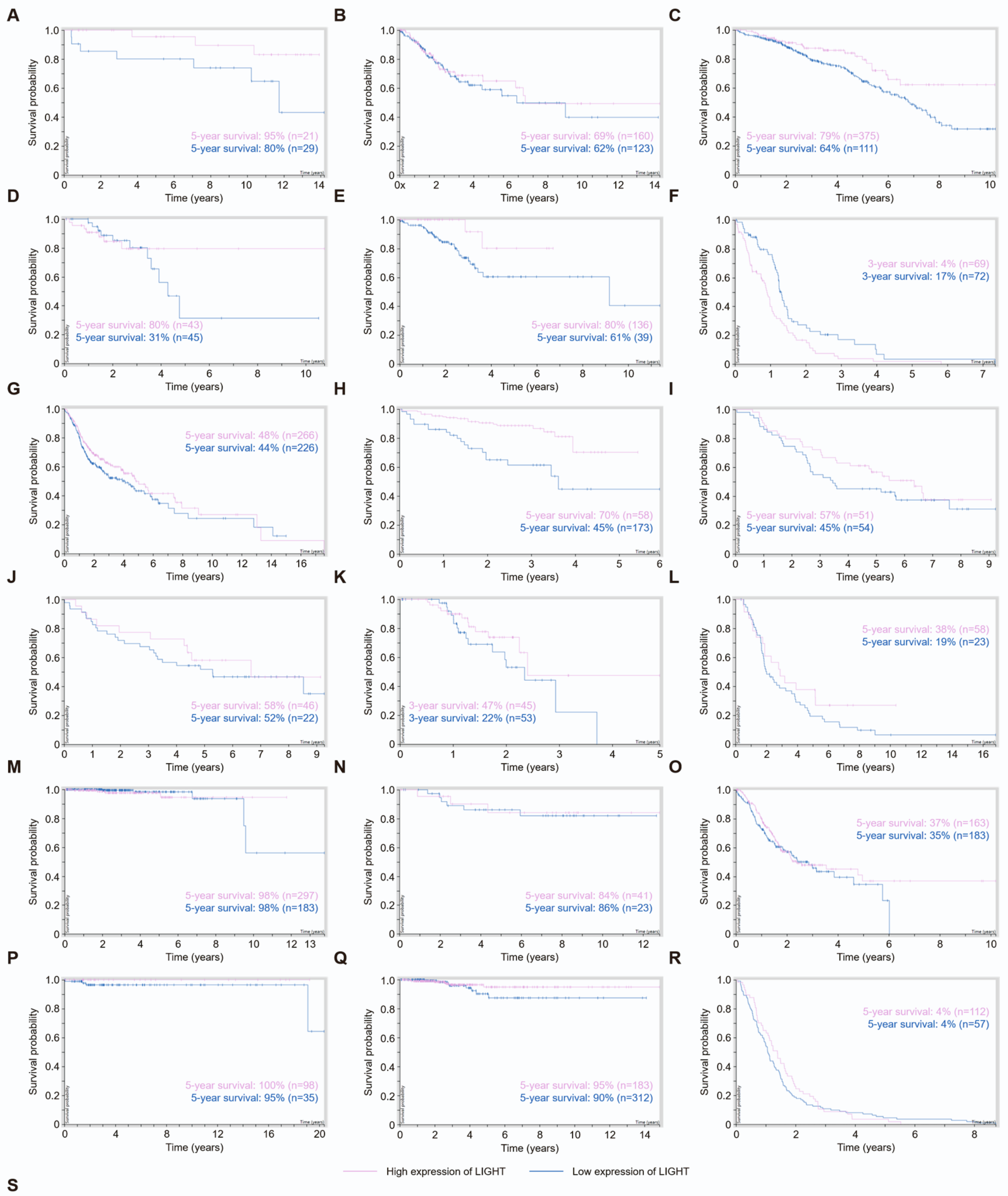

S

Blinatumomab (van der Sluis, I.M. [1])		
Cancer type	Lymphoblastic Leukemia	
Target sites	CD3 CD19	
Group	Blinatumomab	Chemotherapy
Age	Infant	Infant
2-year overall survival (95% CI)	93.3% (75.9 to 98.3)	54.5% (49.9 to 58.9)
2-year disease-free survival (95% CI)	81.6% (60.8 to 92.0)	42.4% (37.9 to 46.8)

Blinatumomab (Litzow, M.R. [2])		
Cancer type	Lymphoblastic Leukemia	
Target sites	CD3 CD19	
Group	Blinatumomab	Chemotherapy
Age	51.5 (30 to 69)	50 (30 to 70)
3-year overall survival	85%	68%
3-year relapse-free survival	80%	64%

Blinatumomab (Kantarjian, H. [3])		
Cancer type	Lymphoblastic Leukemia	
Target sites	CD3 CD19	
Group	Blinatumomab	Chemotherapy
Age	40.8 (18 to 80)	41.1 (18 to 78)
Overall survival (95% CI)	7.7 months (5.6 to 9.6)	4.0 months (2.9 to 5.3)
Event-free survival	31%	12%

Glofitamab (Dickinson, M.J. [4])	
Cancer type	Diffuse Large B-Cell Lymphoma
Target sites	CD3 CD20
Group	Glofitamab
Age	66 (21-90)
6-month progression-free survival (95% CI)	46% (37 to 54)
progression-free survival (95% CI)	4.9 months (3.4 to 8.1)

Tebentafusp (Nathan, P. [5])		
Cancer type	Metastatic Uveal Melanoma	
Target sites	CD3 gp100	
Group	Tebentafusp	Control
Age	64 (23 to 92)	66 (25 to 88)
Overall survival (95% CI)	15.3 months (12 to not reached)	6.5 months (4.9 to 13.4)
6-month progression-free survival	31%	19%

Tebentafusp (Hassel, J.C. [6])		
Cancer type	Metastatic Uveal Melanoma	
Target sites	CD3 gp100	
Group	Tebentafusp	Control
Age	≥18	≥18
Overall survival (95% CI)	21.6 months (19 to 24.3)	16.9 months (12.9 to 19.5)
Progression-free survival (95% CI)	3.4 months (3.0 to 5.4)	2.9 months (2.8 to 3.0)

Talquetamab (Chari, A. [7])		
Cancer type	Multiple Myeloma	
Target sites	CD3 GPRC5D	
Group	Talquetamab	History
Age	63 (41–80)	N/A
12-month progression-free survival (95% CI)	74% (57 to 84)	53% (28 to 73)
18-month progression-free survival (95% CI)	70% (52 to 82)	53% (28 to 73)

Teclistamab (Moreau, P. [8])	
Cancer type	Multiple Myeloma
Target sites	CD3 BCMA
Group	Teclistamab
Age	64.0 (33.0–84.0)
Overall survival (95% CI)	18.3 months (15.1 to not reached)
Progression-free survival (95% CI)	11.3 months (8.8 to 17.1)

Figure S12. Clinical relevance study of LIGHT and αCD3

(A-R) The survival probability of patients with breast invasive carcinoma (A), cervical squamous cell carcinoma and endocervical adenocarcinoma (B), colon adenocarcinoma (C), rectum adenocarcinoma (D),

uterine corpus endometrial carcinoma (E), glioblastoma multiforme (F), head and neck squamous cell carcinoma (G), liver hepatocellular carcinoma (H), lung adenocarcinoma (I), lung squamous cell carcinoma (J), skin melanoma (K), ovary serous cystadenocarcinoma (L), prostate adenocarcinoma (M), kidney chromophobe (N), stomach adenocarcinoma (O), testicular germ cell tumor (P), thyroid carcinoma (Q), and bladder urothelial carcinoma (R) in the low or high expression of LIGHT. The data derived from The Cancer Genome Atlas (TCGA) database.
(S) The clinical trials of anti-CD3 related bispecific T cell engager, including blinatumomab, glofitamab, tebentafusp, talquetamab, and teclistamab. The data derived from the References S1-S8.
Related to Figure 7.

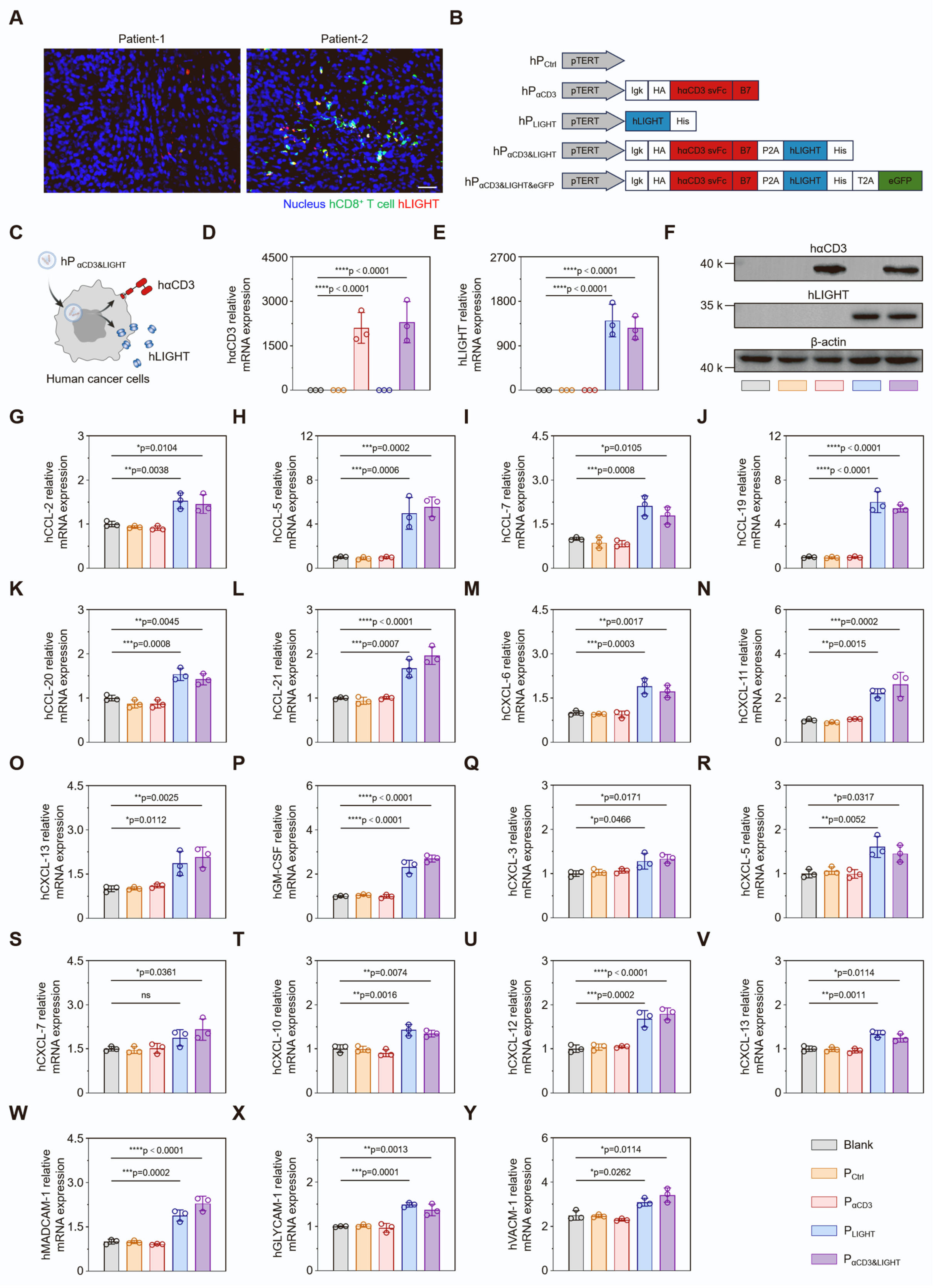

Patient-1
Patient-2
Nucleus hCD8+ T cell hLIGHT
hPCtrl pTERT
hPαCD3 pTERT Igk HA hαCD3 svFc B7
hPLIGHT pTERT hLIGHT His
hPαCD3&LIGHT pTERT Igk HA hαCD3 svFc B7 P2A hLIGHT His
hPαCD3&LIGHT&eGFP pTERT Igk HA hαCD3 svFc B7 P2A hLIGHT His T2A eGFP
hP αCD3&LIGHT
hαCD3
hLIGHT
Human cancer cells
hαCD3 relative mRNA expression
****p < 0.0001
hLIGHT relative mRNA expression
hαCD3
hLIGHT
β-actin
40 k
35 k
hCCL-2 relative mRNA expression
*p=0.0104
**p=0.0038
hCCL-5 relative mRNA expression
***p=0.0002
***p=0.0006
hCCL-7 relative mRNA expression
*p=0.0105
***p=0.0008
hCCL-19 relative mRNA expression
hCCL-20 relative mRNA expression
**p=0.0045
***p=0.0008
hCCL-21 relative mRNA expression
***p=0.0007
hCXCL-6 relative mRNA expression
**p=0.0017
***p=0.0003
hCXCL-11 relative mRNA expression
***p=0.0002
**p=0.0015
hCXCL-13 relative mRNA expression
**p=0.0025
*p=0.0112
hGM-CSF relative mRNA expression
hCXCL-3 relative mRNA expression
*p=0.0171
*p=0.0466
hCXCL-5 relative mRNA expression
*p=0.0317
**p=0.0052
hCXCL-7 relative mRNA expression
*p=0.0361
ns
hCXCL-10 relative mRNA expression
**p=0.0074
**p=0.0016
hCXCL-12 relative mRNA expression
***p=0.0002
hCXCL-13 relative mRNA expression
*p=0.0114
**p=0.0011
hMADCAM-1 relative mRNA expression
hGLYCAM-1 relative mRNA expression
**p=0.0013
***p=0.0001
hVACM-1 relative mRNA expression
*p=0.0262
Blank
PCtrl
PαCD3
PLIGHT
PαCD3&LIGHT

Figure S13. Clinical relevance study of LIGHT and αCD3 and $hP_{\alpha CD3\&LIGHT}$-enhanced performance of human CAR-T cells

(A) Representative immunofluorescence images of hLIGHT and $hCD8^+$ T cells in human cervical cancer tissues. Nucleus (Blue), $hCD8^+$ T cell (Green) and hLIGHT (Red). Scale bar: 25 μm.
(B) Schematic illustration of the structures of $hP_{\alpha CD3\&LIGHT}$, $hP_{\alpha CD3\&LIGHT\&eGFP}$, and other control plasmids.
(C) Schematic illustration of hαCD3 and hLIGHT expression in human tumor cells transfected with $hP_{\alpha CD3\&LIGHT}$.
(D-F) Relative mRNA expression levels (D and E) and western blot analysis (F) of the *hαCD3* and *hLIGHT* in Raji cells transfected with $hP_{\alpha CD3\&LIGHT}$ or other controls. $n = 3$.
(G-P) Relative mRNA expression levels of *hCCL-2* (G), *hCCL-5* (H), *hCCL-7* (I), *hCCL-19* (J), *hCCL-20* (K), *hCCL-21* (L), *hCXCL-6* (M), *hCXCL-11* (N), *hCXCL-13* (O), and *hGM-CSF* (P) in hCAFs following co-incubation with Raji cells transfected with $hP_{\alpha CD3\&LIGHT}$ or other controls. $n = 3$.
(Q-Y) Relative mRNA expression of *hCXCL-3* (Q), *hCXCL-5* (R), *hCXCL-7* (S), *hCXCL-10* (T), *hCXCL-12* (U), *hCXCL-13* (V), *hMADCAM-1* (W), *hGLYCAM-1* (X), and *hVACM-1* (Y) in HUVECs following co-incubation with Raji cells transfected with $hP_{\alpha CD3\&LIGHT}$ or other controls. $n = 3$.
Data are represented as mean ± SD (error bars) from biological replicates. p values were determined by one-way ANOVA with tukey test for (D), (E) and (G-Y). n.s., not significant; $^*p < 0.05$; $^{**}p < 0.01$; $^{***}p < 0.001$; $^{****}p < 0.0001$. Related to Figure 7.

Table S1. Primer sequences. Related to STAR Methods.

Gene Name	Forward Sequence	Reverse Sequence
Plasmid expression		
Mouse αCD3	CCCTGGGAGACAGAGTCAC	ATCTCTCCCAGAACCACTGC
Mouse LIGHT	AGATCAACGATCTCACCAGG	GCTGCACTTTGGAGTACACA
Mouse GAPDH	CATCACTGCCACCCAGAAGACTG	ATGCCAGTGAGCTTCCCGTTCAG
Human αCD3	GAGGTGCAGCTCGTGGAAAGC	TTCTAGCCACCCACTCCAGGC
Human LIGHT	GGCAGAAGCCACAGAAGACAGAG	GGGTTGACCTCATGGCTTCTTCT
Human β-actin	CACCATTGGCAATGAGCGGTTC	AGGTCTTTGCGGATGTCCACGT
Mouse chemokine and adhesion molecules		
P100	TGCTGATGGCACAGGACGAGAA	GTTGATGACGCCGAGGTACTGA
Mouse CCL-1	TTCCCCTGAAGTTTATCCAGTGTT	TGAACCCACGTTTTGTTAGTTGAG
Mouse CCL-2	GCTACAAGAGGATCACCAGCAG	GTCTGGACCCATTCCTTCTTGG
Mouse CCL-3	ACTGCCTGCTGCTTCTCCTACA	ATGACACCTGGCTGGGAGCAAA
Mouse CCL-4	ACCCTCCCACTTCCTGCTGTTT	CTGTCTGCCTCTTTTGGTCAGG
Mouse CCL-5	AGATCTCTGCAGCTGCCCTCA	GGAGCACTTGCTGCTGGTGTAG
Mouse CCL-6	CACCAGTGGTGGGTGCATCAAG	GTGCTTAGGCACCTCTGAACTC
Mouse CCL-7	AAGATCCCCAAGAGGAATCTCAAG	CAGACTTCCATGCCCTTCTTTG
Mouse CCL-8	GGGTGCTGAAAAGCTACGAGAG	GGATCTCCATGTACTCACTGACC
Mouse CCL-9	TCCAGAGCAGTCTGAAGGCACA	CCGTGAGTTATAGGACAGGCAG
Mouse CCL-11	TCCATCCCAACTTCCTGCTGCT	CTCTTTGCCCAACCTGGTCTTG
Mouse CCL-12	GCTACAGGAGAATCACAAGCAGC	ACGTCTTATCCAAGTGGTTTATGG
Mouse CCL-17	TGCTTCTGGGGACTTTTCTG	GAATGGCCCCTTTGAAGTA
Mouse CCL-19	TCGTGAAAGCCTTCCGCTACCT	CAGTCTTCGGATGATGCGATCC
Mouse CCL-20	GTGGGTTTCACAAGACAGATGGC	CCAGTTCTGCTTTGGATCAGCG
Mouse CCL-21	GGGTCAGGACTGCTGCCTTAAG	AGCTCAGGCTTAGAGTGCTTCC
Mouse CCL-22	GTGGAAGACAGTATCTGCTGCC	AGGCTTGCGGCAGGATTTTGAG
Mouse CCL-24	ATTCTGTGACCATCCCCTCAT	TGTATGTGCCTCTGAACCCAC
Mouse CXCL-1	TCCAGAGCTTGAAGGTGTTGCC	AACCAAGGGAGCTTCAGGGTCA
Mouse CXCL-2	CATCCAGAGCTTGAGTGTGACG	GGCTTCAGGGTCAAGGCAAACT
Mouse CXCL-3	TGAGACCATCCAGAGCTTGACG	CCTTGGGGGTTGAGGCAAACTT
Mouse CXCL-4	GTTGTTTCTGCCAGCGGTGGTT	ACAGTGGCGTCCTGCCTTGATC
Mouse CXCL-5	CCGCTGGCATTTCTGTTGCTGT	CAGGGATCACCTCCAAATTAGCG
Mouse CXCL-9	CCTAGTGATAAGGAATGCACGATG	CTAGGCAGGTTTGATCTCCGTTC
Mouse CXCL-10	ATCATCCCTGCGAGCCTATCCT	GACCTTTTTTGGCTAAACGCTTTC
Mouse CXCL-11	CCGAGTAACGGCTGCGACAAAG	CCTGCATTATGAGGCGAGCTTG
Mouse CXCL-12	GGAGGATAGATGTGCTCTGGAAC	AGTGAGGATGGAGACCGTGGTG
Mouse CXCL-13	CATAGATCGGATTCAAGTTACGCC	GTAACCATTTGGCACGAGGATTC
Mouse CXCL-16	GCAGGGTACTTTGGATCACATCC	AGTTCACGGACCCACTGGTCTT
Mouse CX3CL-1	CAGTGGCTTTGCTCATCCGCTA	AGCCTGGTGATCCAGATGCTTC
Mouse GM-CSF	ATGCCTGTCACGTTGAATGAAG	GCGGGTCTGCACACATGTTA
Mouse PECAM-1	CCAAAGCCAGTAGCATCATGGTC	GGATGGTGAAGTTGGCTACAGG
Mouse MADCAM-1	TGTCAGACACAGGCACTCCTGT	CTGTCCAGGTACAAGGAACTCC
Mouse GLYCAM-1	AAGACTCAGCCCACAGATGCCA	CTCTGAAGATGGAAGGCTCCTTG
Mouse VCAM-1	GCTATGAGGATGGAAGACTCTGG	ACTTGTGCAGCCACCTGAGATC
Mouse ICAM-1	AAACCAGACCCTGGAACTGCAC	GCCTGGCATTTCAGAGTCTGCT
Mouse VEGF-C	CCTGAATCCTGGGAAATGTGCC	CGATTCGCACACGGTCTTCTGT

Mouse matrix metalloproteinase (MMPs)		
Mouse MMP-1	AGGAAGGCGATATTGTGCTCTCC	TGGCTGGAAAGTGTGAGCAAGC
Mouse MMP-2	CAAGGATGGACTCCTGGCACAT	TACTCGCCATCAGCGTTCCCAT
Mouse MMP-3	CTCTGGAACCTGAGACATCACC	AGGAGTCCTGAGAGATTTGCGC
Mouse MMP-7	AGGTGTGGAGTGCCAGATGTTG	CCACTACGATCCGAGGTAAGTC
Mouse MMP-8	GATGCTACTACCACACTCCGTG	TAAGCAGCCTGAAGACCGTTGG
Mouse MMP-9	TGCTGCCTATGAGGCTCACAAC	GGAGGAAAACCGAGAGTGTGGA
Mouse MMP-10	TGCTGCCTATGAGGCTCACAAC	GGAGGAAAACCGAGAGTGTGGA
Mouse MMP-11	GATTGATGCTGCCTTCCAGGATG	CAGCGGAAAGTATTGGCAGGCT
Mouse MMP-12	CACACTTCCCAGGAATCAAGCC	TTTGGTGACACGACGGAACAGG
Mouse MMP-13	GATGACCTGTCTGAGGAAGACC	GCATTTCTCGGAGCCTGTCAAC
Mouse MMP-14	GGATGGACACAGAGAACTTCGTG	CGAGAGGTAGTTCTGGGTTGAG
Mouse MMP-15	CTGAGCAGCTATGGCACAGACA	TGCTGTGTCTCCTCGTTGAAGC
Mouse MMP-16	GAGACCATGCAGTCAGCTCTAG	TAGAGCTGCCTCTTGTCTGGTC
Mouse MMP-17	TGGACTTCCCTATGAGGACAGG	GGAGTGCTAGACCGATTGTTGG
Mouse MMP-19	AGGCACTCATGGCTCCTGTCTA	TGAGCATCTCGGTCTCTTCCTC
Mouse MMP-20	GAAGTGGCTGAACGAGGCATTG	TTGTCCGTGGAGGACCTTGCAT
Mouse MMP-21	TGAGGTGACACCACTGGACTTC	AGGCATGTGCAAACTCCTGTCC
Mouse MMP-23	CCACTGTGGACAGAAGATCCTAC	TGTAGGTGCCTTCGTTGACTGC
Mouse MMP-24	TCAAGCTACCGCAGGACGATCT	TGCTTCCTCTCAGATGGCGAGT
Mouse MMP-25	CGTCGCTATTCTCTGAGTGGCA	GCCTGATTCAGTAGCCCAGACA
Mouse MMP-27	CACACTCGGATTTCCAAGACGTG	TCCTCTGTCCATTGCTTGTGCC
Mouse MMP-28	CTTGCTGGACACCGAGCCAAAA	CAGTTCACCAGGCGGTAGGAAA
Human chemokine and adhesion molecules		
Human CCL-1	ACCAGCTCCATCTGCTCCAATG	TGTGCCTCTGAACCCATCCAAC
Human CCL-2	AGAATCACCAGCAGCAAGTGTCC	TCCTGAACCCACTTCTGCTTGG
Human CCL-3	ACTTTGAGACGAGCAGCCAGTG	TTTCTGGACCCACTCCTCACTG
Human CCL-4	GCTTCCTCGCAACTTTGTGGTAG	GGTCATACACGTACTCCTGGAC
Human CCL-5	CCTGCTGCTTTGCCTACATTGC	ACACACTTGGCGGTTCTTTCGG
Human CCL-7	ACAGAAGGACCACCAGTAGCCA	GGTGCTTCATAAAGTCCTGGACC
Human CCL-8	CCAATCACCTGCTGCTTTAACGT	TCCCTGACCCATCTCTCCTTGG
Human CCL-11	GCTACAGGAGAATCACCAGTGG	GGAATCCTGCACCCACTTCTTC
Human CCL-17	TTCTCTGCAGCACATCCACGCA	CTGGAGCAGTCCTCAGATGTCT
Human CCL-19	CGTGAGGAACTTCCACTACCTTC	GTCTCTGGATGATGCGTTCTACC
Human CCL-20	AAGTTGTCTGTGTGCGCAAATCC	CCATTCCAGAAAAGCCACAGTTTT
Human CCL-21	AGCAGGAACCAAGCTTAGGCTG	GGTGTCTTGTCCAGATGCTGCA
Human CCL-22	TCCTGGGTTCAAGCGATTCTCC	GTCAGGAGTTCAAGACCAGCCT
Human CCL-23	CCGTGTTCACTCCTGGAGAGTT	GCTTCAGCATTCTCACGCAAACC
Human CCL-24	TTCCTTGGTGTCTGTGCCCAC	CACAGAACTGCTGGCCCTTCT
Human CXCL-1	AGCTTGCCTCAATCCTGCATCC	TCCTTCAGGAACAGCCACCAGT
Human CXCL-2	GGCAGAAAGCTTGTCTCAACCC	CTCCTTCAGGAACAGCCACCAA
Human CXCL-3	TTCACCTCAAGAACATCCAAAGTG	TTCTTCCCATTCTTGAGTGTGGC
Human CXCL-4	TCCTGCCACTTGTGGTCGCCT	CCTTGATCACCTCCAGGCTGG
Human CXCL-5	CAGACCACGCAAGGAGTTCATC	TTCCTTCCCGTTCTTCAGGGAG
Human CXCL-6	GGGAAGCAAGTTTGTCTGGACC	AAACTGCTCCGCTGAAGACTGG
Human CXCL-7	TGCTCTGGCTTCCTCCACCAAA	ACACATGCAGCGGAGTTCAGCA
Human CXCL-8	GAGAGTGATTGAGAGTGGACCAC	CACAACCCTCTGCACCCAGTTT
Human CXCL-9	CTGTTCCTGCATCAGCACCAAC	TGAACTCCATTCTTCAGTGTAGCA

Human CXCL-10	GGTGAGAAGAGATGTCTGAATCC	GTCCATCCTTGGAAGCACTGCA
Human CXCL-11	AAGGACAACGATGCCTAAATCCC	CAGATGCCCTTTTCCAGGACTTC
Human CXCL-12	CTCAACACTCCAAACTGTGCCC	CTCCAGGTACTCCTGAATCCAC
Human CXCL-13	TATCCCTAGACGCTTCATTGATCG	CCATTCAGCTTGAGGGTCCACA
Human CXCL-14	AGATCCGCTACAGCGACGTGAA	GCAGTGCTCCTGACCTCGGTA
Human CXCL-16	CCTATGTGCTGTGCAAGAGGAG	CTGGGCAACATAGAGTCCGTCT
Human GM-CSF	GGAGCATGTGAATGCCATCCAG	CTGGAGGTCAAACATTTCTGAGAT
Human PECAM-1	AAGTGGAGTCCAGCCGCATATC	ATGGAGCAGGACAGGTTCAGTC
Human MADCAM-1	AGGCTCCACCAGGACTCGCC	ACCGCACTGCTGGTCCACAGA
Human VCAM-1	GATTCTGTGCCCACAGTAAGGC	TGGTCACAGAGCCACCTTCTTG
Human ICAM-1	AGCGGCTGACGTGTGCAGTAAT	TCTGAGACCTCTGGCTTCGTCA
Human VEGF-C	GCCAATCACACTTCCTGCCGAT	AGGTCTTGTTCGCTGCCTGACA
T cell activation and exhaustion		
CD69	GGGCTGTGTTAATAGTGGTCCTC	CTTGCAGGTAGCAACATGGTGG
CD44	CGGAACCACAGCCTCCTTTCAA	TGCCATCCGTTCTGAAACCACG
CD25	GCGTTGCTTAGGAAACTCCTGG	GCATAGACTGTGTTGGCTTCTGC
PD-1	CGGTTTCAAGGCATGGTCATTGG	TCAGAGTGTCGTCCTTGCTTCC
TIM-3	ACAGACACTGGTGACCCTCCAT	CAGCAGAGACTCCCACTCCAAT
LAG-3	CTCCATCACGTACAACCTCAAGG	GGAGTCCACTTGGCAATGAGCA
CD39	CTGGACAAGAGGAAGGTGCCTA	GACTGTCTGAGATGAGGCTTAGC

Table S2. The information of tumor samples included in this study. Related to STAR Methods.

Sample	Malignancy	Gender	Age	Treatment	Sample retrieval	Influence of gender
Sample 1	Colorectal cancer	Male	74	No	Primary tumor	The expression of hLIGHT: P value = 0.4946<br><br>The number of $hCD8^+$ T cells: P value = 0.6849
Sample 2	Colorectal cancer	Male	72	No	Primary tumor	
Sample 3	Colorectal cancer	Female	69	No	Primary tumor	
Sample 4	Colorectal cancer	Male	53	No	Primary tumor	
Sample 5	Colorectal cancer	Male	48	Unknown	Primary tumor	
Sample 6	Colorectal cancer	Female	60	No	Primary tumor	
Sample 7	Colorectal cancer	Female	50	No	Primary tumor	
Sample 8	Colorectal cancer	Male	61	No	Primary tumor	
Sample 9	Colorectal cancer	Male	46	No	Primary tumor	
Sample 10	Colorectal cancer	Female	55	No	Primary tumor	
Sample 11	Colorectal cancer	Male	37	No	Primary tumor	
Sample 12	Colorectal cancer	Female	79	No	Primary tumor	
Sample 13	Nasopharyngeal carcinoma	Male	69	No	Primary tumor	The expression of hLIGHT: P value = 0.2903<br><br>The number of $hCD8^+$ T cells: P value = 0.2682
Sample 14	Nasopharyngeal carcinoma	Male	47	No	Primary tumor	
Sample 15	Nasopharyngeal carcinoma	Male	68	No	Primary tumor	
Sample 16	Nasopharyngeal carcinoma	Female	59	Unknown	Primary tumor	
Sample 17	Nasopharyngeal carcinoma	Male	67	No	Primary tumor	
Sample 18	Nasopharyngeal carcinoma	Female	64	No	Primary tumor	
Sample 19	Nasopharyngeal carcinoma	Male	71	No	Primary tumor	
Sample 20	Nasopharyngeal carcinoma	Male	58	No	Primary tumor	
Sample 21	Nasopharyngeal carcinoma	Male	73	No	Primary tumor	
Sample 22	Nasopharyngeal carcinoma	Female	37	No	Primary tumor	
Sample 23	Cervical cancer	Female	47	Unknown	Primary tumor	N/A
Sample 24	Cervical cancer	Female	52	No	Primary tumor	
Sample 25	Cervical cancer	Female	43	No	Primary tumor	
Sample 26	Cervical cancer	Female	39	No	Primary tumor	
Sample 27	Cervical cancer	Female	60	No	Primary tumor	
Sample 28	Cervical cancer	Female	46	No	Primary tumor	
Sample 29	Cervical cancer	Female	58	No	Primary tumor	
Sample 30	Cervical cancer	Female	69	No	Primary tumor	

N/A = Not Applicable (cervical cancer occurs exclusively in female)

REFERENCES

S1. van der Sluis, I.M., de Lorenzo, P., Kotecha, R.S., Attarbaschi, A., Escherich, G., Nysom, K., Stary, J., Ferster, A., Brethon, B., Locatelli, F., et al. (2023). Blinatumomab Added to Chemotherapy in Infant Lymphoblastic Leukemia. N. Engl. J. Med. *388*, 1572-1581. https://doi.org/10.1056/NEJMoa2214171.

S2. Litzow, M.R., Sun, Z., Mattison, R.J., Paietta, E.M., Roberts, K.G., Zhang, Y., Racevskis, J., Lazarus, H.M., Rowe, J.M., Arber, D.A., et al. (2024). Blinatumomab for MRD-Negative Acute Lymphoblastic Leukemia in Adults. N. Engl. J. Med. *391*, 320-333. https://doi.org/10.1056/NEJMoa2312948.

S3. Kantarjian, H., Stein, A., Gökbuget, N., Fielding, A.K., Schuh, A.C., Ribera, J.-M., Wei, A., Dombret, H., Foà, R., Bassan, R., et al. (2017). Blinatumomab versus Chemotherapy for Advanced Acute Lymphoblastic Leukemia. N. Engl. J. Med. *376*, 836-847. https://doi.org/10.1056/NEJMoa1609783.

S4. Dickinson, M.J., Carlo-Stella, C., Morschhauser, F., Bachy, E., Corradini, P., Iacoboni, G., Khan, C., Wróbel, T., Offner, F., Trněný, M., et al. (2022). Glofitamab for Relapsed or Refractory Diffuse Large B-Cell Lymphoma. N. Engl. J. Med. *387*, 2220-2231. https://doi.org/10.1056/NEJMoa2206913.

S5. Nathan, P., Hassel, J.C., Rutkowski, P., Baurain, J.-F., Butler, M.O., Schlaak, M., Sullivan, R.J., Ochsenreither, S., Dummer, R., Kirkwood, J.M., et al. (2021). Overall Survival Benefit with Tebentafusp in Metastatic Uveal Melanoma. N. Engl. J. Med. *385*, 1196-1206. https://doi.org/10.1056/NEJMoa2103485.

S6. Hassel, J.C., Piperno-Neumann, S., Rutkowski, P., Baurain, J.-F., Schlaak, M., Butler, M.O., Sullivan, R.J., Dummer, R., Kirkwood, J.M., Orloff, M., et al. (2023). Three-Year Overall Survival with Tebentafusp in Metastatic Uveal Melanoma. N. Engl. J. Med. *389*, 2256-2266. https://doi.org/10.1056/NEJMoa2304753.

S7. Chari, A., Minnema, M.C., Berdeja, J.G., Oriol, A., van de Donk, N.W.C.J., Rodríguez-Otero, P., Askari, E., Mateos, M.-V., Costa, L.J., Caers, J., et al. (2022). Talquetamab, a T-Cell–Redirecting GPRC5D Bispecific Antibody for Multiple Myeloma. N. Engl. J. Med. *387*, 2232-2244. https://doi.org/10.1056/NEJMoa2204591.

S8. Moreau, P., Garfall, A.L., van de Donk, N.W.C.J., Nahi, H., San-Miguel, J.F., Oriol, A., Nooka, A.K., Martin, T., Rosinol, L., Chari, A., et al. (2022). Teclistamab in Relapsed or Refractory Multiple Myeloma. N. Engl. J. Med. *387*, 495-505. https://doi.org/10.1056/NEJMoa2203478.
